# Synthesis of chiral malonates by α-alkylation of 2,2-diphenylethyl *tert*-butyl malonates via enantioselective phase-transfer catalysis

**DOI:** 10.3389/fchem.2023.1205661

**Published:** 2023-06-08

**Authors:** Zhibin Guo, Daehyun Oh, Min Sagong, Jewon Yang, Geumwoo Lee, Hyeung-geun Park

**Affiliations:** Research Institute of Pharmaceutical Sciences, College of Pharmacy, Seoul National University, Seoul, Republic of Korea

**Keywords:** asymmetric synthesis, phase-transfer catalysis, enantioselective, alkylation, organocatalysis

## Abstract

An efficient synthetic approach for chiral malonates was established via enantioselective phase transfer catalysis. The α-alkylation of 2,2-diphenylethyl tert-butyl α-methylmalonates with (S,S)-3,4,5-trifluorophenyl-NAS bromide as a phase-transfer catalyst under phase-transfer catalytic conditions successfully produced corresponding α-methyl-α-alkylmalonates; these compounds are versatile chiral building blocks containing a quaternary carbon center in high chemical yields (up to 99%) with excellent enantioselectivities (up to 98% ee). α,α-Dialkylmalonates were selectively hydrolyzed to the corresponding chiral malonic monoacids under basic (KOH/MeOH) and acidic conditions (TFA/CH_2_Cl_2_), showing the practicality of the method.

## 1 Introduction

The carbon skeleton is important in organic molecules regarding their molecular characterization and biological activity. As a highly essential synthetic starting materials, 1,3-dicarbonyl type compounds have been used to form C-C bonds for the construction of carbon skeletons of organic molecules including natural products and pharmaceuticals, by coupling with carbonic electrophiles, such as alkyl halides, imines, and carbonyl compounds including α,β-unsaturated carbonyls (Carruthers and Coldham, 2004). To construct chiral carbon centers of organic molecules, chiral malonates have been widely utilized among the 1,3-dicarbonyl molecules (Pàmies et al., 2021; Wright and Evans, 2021) because the two esters of the chiral malonates are selectively convertible (Behenna et al., 2012; Zhang et al., 2019). The most common methods to obtain chiral α,α-dialkylmalonates are the enzymatic resolution of (±)-α,α-dialkylmalonic acids or (±)-α,α-dialkylmalonates and chrial high performance liquid chromatography (HPLC) resolution (Faber, 2011). However, in the case of chemical synthesis, only a few methods have been reported via enantioselective α-alkylation using chiral auxiliary or α-fluorination using organometallic catalysis (Reddy et al., 2008; Bixa et al., 2015; Gokada et al., 2017). In 2011, Itoh group successfully reported organocatalytic method by employing enantioselective α-alkylation using *cinchona* derived ammonium salt (Kanemitsu et al., 2011).

**SCHEME 1:**
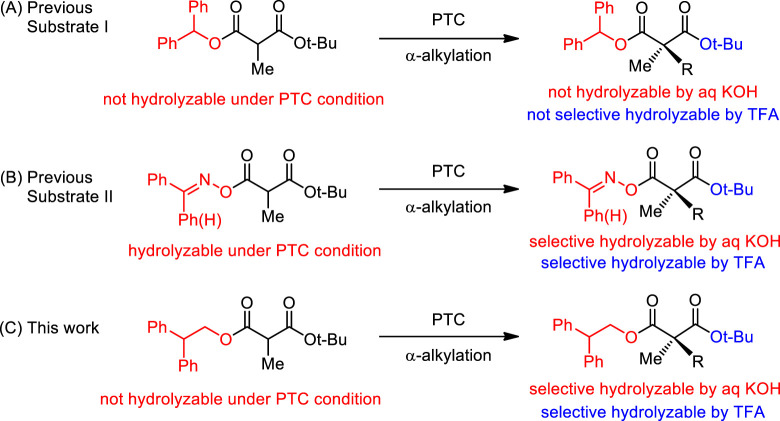
Synthetic strategy for chiral quaternary α-methylmalonates.

Recently, our research group reported the first enantioselective catalytic direct α-alkylation of malonates with high efficiencies in chemical yields and enantioselectivities through phase-transfer catalytic (PTC) α-alkylation of diphenylmethyl *tert*-butyl α-alkylmalonate (**1**) (Scheme 1A) (Hong et al., 2011). The resulting chiral α,α-dialkylmalonates could be selectively modified to chiral malonic monoacids by catalytic hydrogenation. The monoacids were converted to versatile building blocks and successfully applied to the total synthesis of (−)-horsfiline and (+)-coerulescine (Hong et al., 2013; Lee et al., 2020). However, selective conversion to monoacids was not successful under acidic and basic conditions. Both diphenylmethyl ester and *tert*-butyl ester groups could not be hydrolyzed in alkaline basic conditions due to their steric hindrance. In the case of acidic conditions, *tert*-butyl ester was hydrolyzed, however, diphenylmethyl ester was also partially hydrolyzed. For the selective conversion to monoacids in both acidic and basic conditions, we modified the malonate substrate structure by the displacement of the diphenylmethyl ester to a benzylideneamino ester. The newly developed substrate showed high enantioselectivity in PTC α-alkylations and selective hydrolysis under both acidic and basic conditions (Scheme 1B) (Park et al., 2015). However, the benzylideneamino group was partially hydrolyzed during the reaction under alkaline basic PTC conditions when the reaction time was relatively long resulting low chemical yields. In this paper, we report new malonate substrates for highly enantioselective phase-transfer catalytic α-alkylation whose two ester groups can be selectively hydrolyzed by either acidic or basic conditions with no hydrolysis under PTC basic conditions (Jew and Park, 2009; Shirakawa and Maruoka, 2013).

## 2 Results and discussion

**SCHEME 2:**
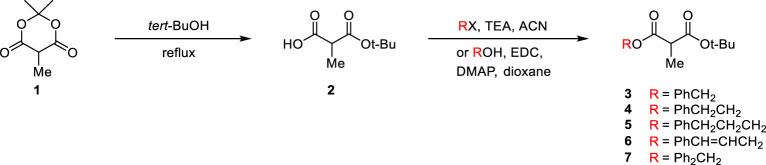
Preparation of α-methylmalonates.

Since the *tert*-butyl ester group is generally known to be essential for high enantioselectivity in PTC alkylation, we designed new malonate substrates by replacing the benzylidenamino ester group of the malonate substrate (Scheme 1C). The partial hydrolysis of the benzylidenamino ester group under alkaline basic PTC reaction conditions was potentially due to the high leaving ability of benzylidenoxime in alkaline basic hydrolysis. Therefore, we selected alcoholic esters that were more resistant to hydrolysis in alkaline-base environments. A series of malonates, containing phenyl group with a space between it and carbonyl group, were prepared in two steps from α-methyl Meldrum’s acid (**1**) as shown in Scheme 2. The transesterification of α-methyl Meldrum’s acid with *tert*-butanol followed by decarboxylation produced *tert*-butyl α-methylmalonic acid (**2**). The coupling of 2 with various alcohols using 1-ethyl-3-(dimethylaminopropyl)carbodiimide (EDC) in the presence of 4-dimethylaminopyridine (DMAP) successfully afforded the corresponding α-methylmalonates (**3**–**7**).

**FIGURE 1:**
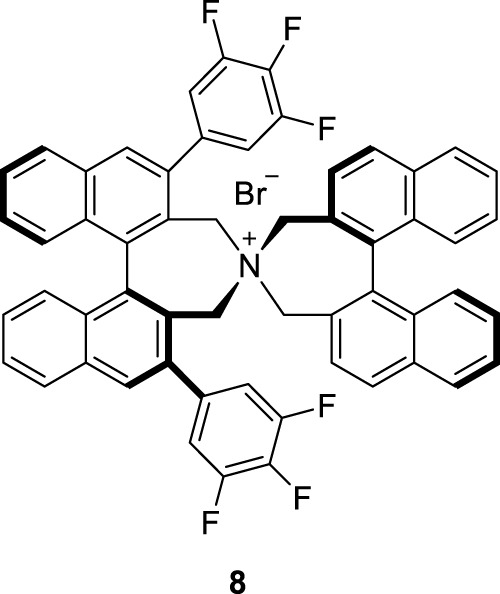
Chiral phase-transfer catalyst.

To optimize the substrate among **3**–**7**, we examined the chemical yields and enantioselectivity of PTC α-benzylation under previously reported PTC conditions (Hong et al., 2013; Lee et al., 2020). PTC α-benzylation was carried out with benzyl bromide (5.0 equiv.) and 50% KOH (aq., 5.0 equiv.) at 0 °C in toluene with catalyst **8** (Figure 1), which was already optimized by previous studies (Hong et al., 2011; Park et al., 2015). As shown in Table 1, all substrates successfully produced the corresponding α-benzylated products. However, the enantioselectivity varied depending on the alkyl groups in the ester. Generally, a longer linker resulted in lower enantioselectivities (entries 1–3). Cinnamyl substrate (**6**) showed a high chemical yield with moderate enantioselectivity (entry 4). The highest enantioselectivity was observed in the diphenylmethyl substrate (**7**, entry 5). We expected that the π–π stacking interactions between PTC catalyst **8** and the diphenyl group in substrate **7** could cause tighter ionic binding compared to one phenyl group in substrate **3**, which afforded higher enantioselectivities (entries 2 and 5).

**TABLE 1:** Enantioselective PTC α-benzylation of alkyl *tert*-butyl α-methylmalonates (**3**–**7**).^a^

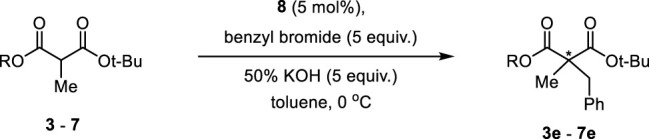					
No	R	Substrate	Time (h)	Yield (%)^b^	*Ee* (%)^c^
1	PhCH_2_	**3**	3	81	77
2	PhCH_2_CH_2_	**4**	5	86	60
3	PhCH_2_CH_2_CH_2_	**5**	2	41	53
4	PhCH = CHCH_2_	**6**	4	96	56
5	Ph_2_CHCH_2_	**7**	3	80	88

^a^ Reactions were performed with 5.0 equiv. of benzyl bromide and 5.0 equiv. of 50% KOH (*aq*.) under the given conditions.

^b^ Isolated yields.

^c^ Enantioselectivity was determined by HPLC, analysis using a chiral column (DAICEL, Chiralpak AD-H, and AS-H, Chiralcel OD-H, and OJ-H).

Next, the solvent, temperature and base conditions were optimized with the optimal substrate **7** and PTC catalyst **8**. As shown in Table 2, generally, the chemical yield and enantioselectivity did not have a significant dependence on the base at 0 °C. However, K_2_CO_3_ showed a low chemical yield (entry 6), and CsOH caused no reaction (entry 5). In CH_2_Cl_2_ and THF, a significant decrease in enantioselectivity was observed (entries 7 and 8). Regarding the temperature, a lower reaction temperature generally caused higher enantioselectivities (entries 9–11). However, a low chemical yield and longer reaction time were observed at −60°C (entry 11). According to the enantioselectivity, chemical yield and reaction time, we finally selected the reaction conditions (50% KOH, toluene, −40°C) of entry 10 in Table 2 as the optimized PTC condition (entry 10; 75%, 95% ee, 30 h).

**TABLE 2:** Optimization of the reaction conditions.^a^

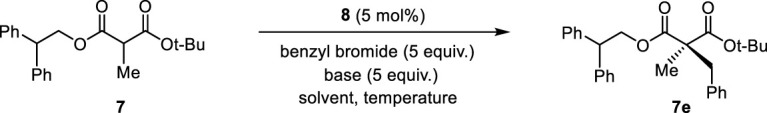						
No	Base	T (°C)	Solvent	Time (h)	Yield (%)^b^	*Ee* (%)^c^
1	50% NaOH	0	toluene	23	30	90
2	50% KOH	0	toluene	17	80	88
3	50% CsOH	0	toluene	96	82	93
4	KOH	0	toluene	96	84	90
5	CsOH	0	toluene	-	-	-
6	K_2_CO_3_	0	toluene	28	13	91
7	50% KOH	0	CH_2_Cl_2_	72	48	40
8	50% KOH	0	THF	72	21	42
9	50% KOH	−20	toluene	24	91	93
10	50% KOH	−40	toluene	30	95	95
11	50% KOH	−60	toluene	96	13	99

^a^ Reactions were performed with 5.0 equiv. of benzyl bromide and 5.0 equiv. of base under the given conditions.

^b^ Isolated yields.

^c^ Enantioselectivity was determined by HPLC, analysis of the corresponding benzylated products **7e** using a chiral column (DAICEL, Chiralcel OJ-H).

**FIGURE 2:**
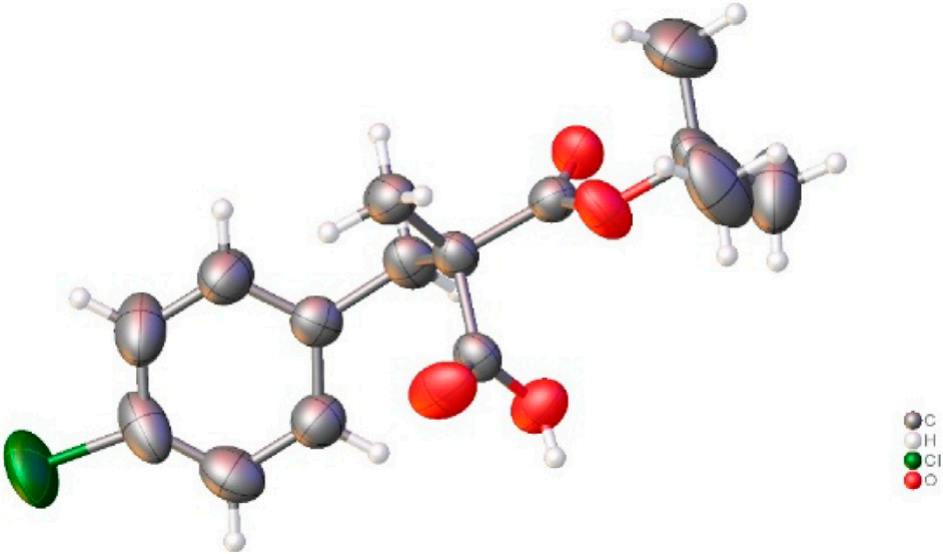
X-ray crystallographic structure of monoacid **10i** prepared from **7i**.

**SCHEME 3:**
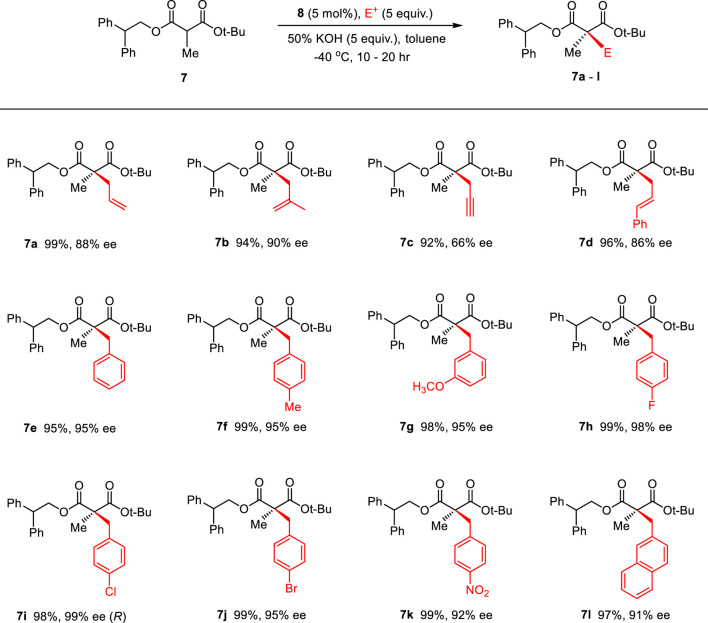
Enantioselective synthesis of α-methyl-α-alkylmalonates via the PTC α-alkylation.

**SCHEME 4:**
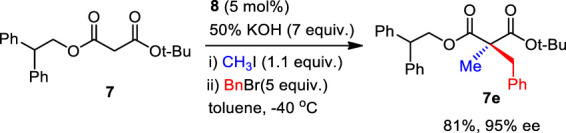
Successive PTC α,α-dialkylations.

Then, the scope and limitations of alkylating agents were investigated under the optimal reaction conditions (Scheme 3). Allylic halides (**7a**, **7b**, **7d**, 70%–99%, 86%–90% ee) and benzylic halides (**7e**–**7i**, 90%–99%, 91%–99% ee) showed very high enantioselectivities, with the exception of propargylic halide (**7c**, 70%, 66% ee). The successive addition of methyl iodide and benzyl bromide into reaction also successfully provided α-methyl-α-benzylmalonate (**7e**) without loss of chemical yield and enantioselectivity (Scheme 4). The broad scope, high chemical yields (up to 99%) and enantioselectivities (up to 98% ee) in Scheme 3 demonstrated that this reaction was an efficient methodology for the synthesis of chiral α,α-dialkylmalonates. The absolute stereochemistry of **7i** was determined by X-ray crystallographic analysis of mono-acid **10i** prepared from the alkali base hydrolysis of **7i** (Figure 2).

**FIGURE 3:**
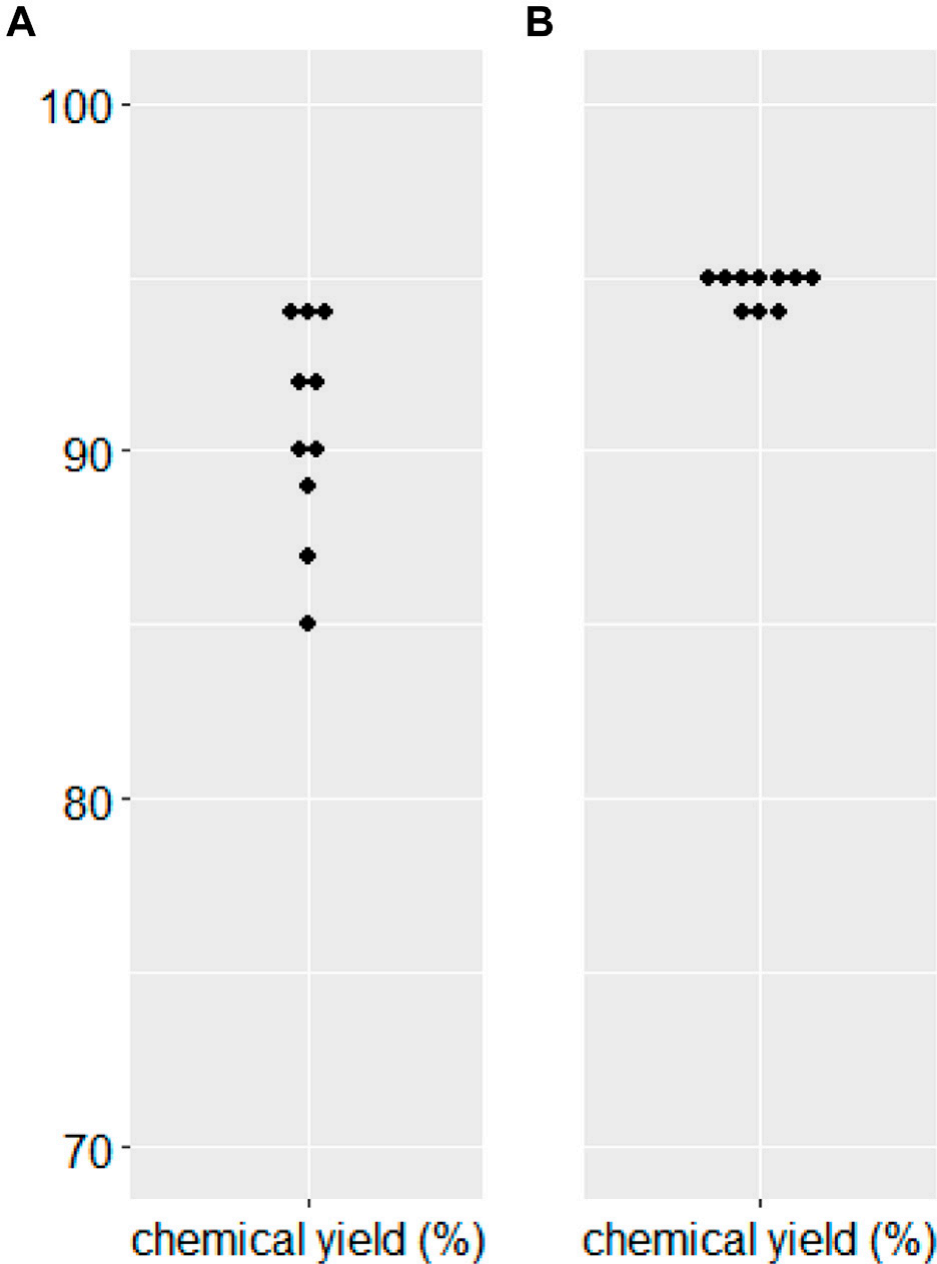
Reproducibility of chemical yields in PTC benzylation. **(A)** Benzylideneamino *tert*-butyl α-methylmalonate **(B)** 2,2-Diphenylethyl *tert*-butyl malonate (**7**).

**SCHEME 5:**
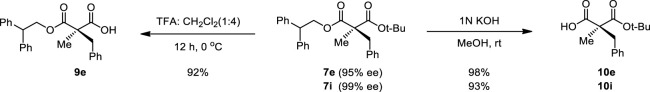
Selective hydrolysis of **7e** and **7i** under basic and acidic conditions.

We needed to confirm the stability of substrate **7** and the corresponding α-alkylated product **7e**. There was no significant hydrolysis of either substrate **7** or product **7e** under the two phase alkaline basic PTC reaction conditions without a catalyst for 24 h at −40°C. Further, the validatation of the reproducibility of chemical yield in the α-benzylation of diphenylethyl ester substrate (**7**) along with benzylideneamino ester substrate (Park et al., 2015) 10 times each showed that diphenylethyl ester substrate (**7**) did afford more reproducable chemical yields (Figure 3). To demonstrate the efficiency for synthetic applications, selective hydrolysis in alkaline basic and acidic conditions was performed (Scheme 5). The selective hydrolysis of *tert*-butyl ester of α-methyl-α-benzylmalonate (**7e**) was successfully accomplished to produce the corresponding acid **9e** in the presence of trifluoroacetic acid under methylene chloride at 0°C (93%). Diphenylethyl ester was also selectively hydrolyzed to the corresponding acid **10e** and **10i** by 1N KOH (94%–98%).

## 3 Conclusion

We successfully developed an efficient methodology for the asymmetric synthesis of chiral α,α-dialkylmalonates from diphenylmethyl-*tert*-butyl α-alkylmalonates via enantioselective PTC α-alkylation promoted by using binaphthyl-modified chiral quaternary ammonium salt (**8**). Furthermore, the reaction products were selectively converted to their corresponding two monoacids; these monoacids are versatile intermediates to synthetize chiral molecules containing chiral quaternary carbon centers. There are valuable examples of highly selective catalytic asymmetric synthesis of α,α-dialkylmalonates (up to 99%, up to 98% ee).

## 4 Materials and Methods

### 4.1 Experimental section

#### 4.1.1 General information

All reagents purchased from commercial sources were used without further purification. Commercially available KOH pellet (99%) was grinded to prepare Solid KOH as powder form. 50% w/v aqueous KOH was used as stock solution. The phase-transfer catalyst, (*S*,*S*)-3,4,5-trifluorophenyl-NAS bromide **(8)**, was purchased from the commercial sources. TLC analyses were performed using pre-coated TLC plate (silica gel 60 GF_254_, 0.25 mm). Flash column chromatography was performed on flash silica gel 230–400 mesh size. The values of enantiomeric excess (ee) of chiral products were determined by Hitachi (UV detector L-2400, Pump L-2130 and software LaChrome 890-8800-12) and Waters (UV/Vis detector 2,489, Binary HPLC Pump 1,525 and software Breeze 2 HPLC System 6.20) HPLC, using 4.6 mm × 250 mm Daicel Chiralpak AD-H, AS-H and Chiralcel OD-H, OJ-H. Infrared analyses (KBr pellet) were performed by FT-IR. Nuclear magnetic resonance (^1^H-NMR and ^13^C-NMR) spectra were measured on JEOL JNM-ECZ 400s [400 MHz (^1^H)] spectrometer and 800 MHz Bruker Avance 3 HD Spectrometer. ^1^H-NMR spectra was recorded at 400 MHz with reference to CDCl_3_ (δ 7.24), CD_3_OD (δ 3.31), CD_2_Cl_2_ (δ 5.32) or (CD_3_)_2_SO (δ 2.50). ^13^C-NMR spectra was obtained by 200 MHz spectrometer relative to the central CDCl_3_ (δ 77.0), CD_3_OD (δ 49.0), CD_2_Cl_2_ (δ 54.00) or (CD_3_)_2_SO (δ 39.51) resonance. Coupling constants (*J*) in ^1^H-NMR are in Hz. Low-resolution mass spectra (LRMS) and high-resolution mass spectra (HRMS) were measured on positive-ion FAB by JEOL JMS-700-2 spectrometer. Melting points were measured on Büchi B-540 melting point apparatus and were uncorrected. Optical rotations were measured on JASCO P-2000 polarimeter and calibrated with pure solvent as blank.

##### 4.1.1.1 Procedure for preparation of PTC substrates (A)

α-Methyl meldrum’s acid (**2**, 2 g, 12.6 mmol) was added to stirred tert-BuOH (30 mL). After reflux for 12 h, the reaction mixture was evaporated to afford α-methyl-malonate mono-tert-butyl ester (**3**, 2.2 g, 99% yield) as a colorless oil. Triethylamine (0.88 mL, 6.31 mmol) was added to a stirred solution of α-methyl-malonate mono-tert-butyl ester (**3**, 1 g, 5.74 mmol) in acetonitrile (20 mL). 3-bromo-1-phenyl-1-propene (1.24 g, 6.32 mmol) was added to the reaction mixture. After reflux average 15 hours, the reaction mixture was diluted with dichloromethane (100 mL), quenched with ammonium chloride (150 mL), washed with brine (150 mL), dried over anhydrous MgSO_4_, filtered, and concentrated in vacuo. The residue was purified by column chromatography (silica gel, hexane: EtOAc = 30:1 ~ 40:1) to afford 1-(tert-butyl) 3-cinnamyl 2-methylmalonate (**6**, 1.50 g, 89% yield) as a colorless oil.

##### 4.1.1.2 Typical experimental procedure for enantioselective phase-transfer catalytic alkylation (B)

p-Chlorobenzyl bromide (62.1 mg, 0.324 mmol) was added to a solution of 1-(tert-butyl) 3-(2,2-diphenylethyl) 2-methylmalonate (**7**, 23 mg, 0.065 mmol) and (*S,S*)-3,4,5-trifluorophenyl-NAS bromide (**8**, 3 mg, 0.0033 mmol) in toluene (216 μL) at room temperature. At the designated low temperature, 50% w/v aqueous KOH (36.4 μL, 0.324 mmol) was added to the reaction mixture and stirred until the starting material disappeared. After completion of the reaction, the reaction mixture was diluted with ethyl acetate (20 mL), washed with brine (10 mL x 2), dried over anhydrous MgSO_4_, filtered, and concentrated *in vacuo*. The residue was purified by flash column chromatography on silica gel eluting with Hexane-EtOAc solution (19:1) to afford 1-(*tert*-butyl) 3-(2,2-diphenylethyl) 2-(4-chlorobenzyl)-2-methylmalonate (**7i**, 29.6 mg, 98% yield) as a colorless oil.

##### 4.1.1.3 Analytical data

###### 4.1.1.3.1 1-Benzyl 3-(*tert*-butyl) 2-methylmalonate (3)

Following the general procedure (**A**) from the compound **2** (500 mg, 2.870 mmol), the molecule **3** was obtained as a colorless oil (591 mg, 78% yield). ^1^H-NMR (400 MHz, CDCl_3_) δ 7.38–7.26 (m, 5H), 5.22–5.10 (m, 2H), 3.37 (q, *J* = 7.3 Hz, 1H), 1.38 (d, *J* = 7.4 Hz, 12H) ppm; ^13^C-NMR (200 MHz, CD_3_OD) δ 172.55, 171.71, 138.47, 130.33, 130.21, 130.14, 83.65, 68.64, 28.83, 14.63 ppm; IR (KBr) 3,902, 3,725, 2,985, 2,348, 2,310, 1731, 1,508, 1,456, 1,371, 1,230, 1,149, 1,097, 1,027, 850, 700, 670, 649, 616 cm^−1^; HRMS (FAB) m/z: [M + H]^+^ Calcd for [C_15_H_21_O_4_]^+^([M + H]^+^) 265.1440; found 265.1443.

###### 4.1.1.3.2 1-(*tert*-Butyl) 3-phenethyl 2-methylmalonate (4)

Following the general procedure (**A**) from the compound **2** (500 mg, 2.870 mmol), the molecule **4** was obtained as a colorless oil (615 mg, 77% yield). ^1^H-NMR (400 MHz, CDCl_3_) δ 7.35–7.15 (m, 5H), 4.40–4.26 (m, 2H), 3.32 (q, *J* = 7.3 Hz, 1H), 2.94 (t, *J* = 7.1 Hz, 2H), 1.42 (s, 9H), 1.34 (d, *J* = 7.3 Hz, 3H) ppm; ^13^C-NMR (200 MHz, CD_3_OD) δ 172.77, 171.69, 139.97, 130.75, 130.32, 128.38, 83.59, 67.67, 36.71, 28.90, 14.69 ppm; IR (KBr) 3,734, 2,921, 2,348, 2,309, 1749, 1730, 1,498, 1,455, 1,369, 1,338, 1,231, 1,148, 1,095, 1,028, 849, 700, 677, 648 cm^−1^; HRMS (FAB) m/z: [M + H]^+^ Calcd for [C_16_H_23_O_4_]^+^([M + H]^+^) 279.1596; found 279.1592.

###### 4.1.1.3.3 1-(*tert*-Butyl) 3-(3-phenylpropyl) 2-methylmalonate (5)

Following the general procedure (**A**) from the compound **2** (500 mg, 2.870 mmol), the molecule **5** was obtained as a colorless oil (520 mg, 62% yield). ^1^H-NMR (400 MHz, CDCl_3_) δ 7.32–7.13 (m, 5H), 4.22–4.06 (m, 2H), 3.39–3.29 (m, 1H), 2.68 (dd, *J* = 8.6, 6.8 Hz, 2H), 2.02–1.90 (m, 2H), 1.51–1.42 (m, 9H), 1.37 (dq, *J* = 7.2, 1.3, 0.8 Hz, 3H) ppm; ^13^C-NMR (200 MHz, CD_3_OD) δ 172.84, 171.86, 143.30, 130.26, 127.85, 83.63, 66.33, 66.32, 33.80, 32.27, 28.96, 14.72, 14.62 ppm; IR (KBr) 3,901, 3,840, 3,734, 3,647, 3,565, 2,348, 2,310, 1748, 1,680, 1,646, 1,564, 1,543, 1,508, 1,488, 1,362, 1,218, 772, 689, 647 cm^−1^; HRMS (FAB) m/z: [M + H]^+^ Calcd for [C_17_H_25_O_4_]^+^([M + H]^+^) 293.1753; found 293.1748.

###### 4.1.1.3.4 1-(*tert*-Butyl) 3-cinnamyl 2-methylmalonate (6)

Following the general procedure (**A**) from the compound **2** (500 mg, 2.870 mmol), the molecule **6** was obtained as a colorless oil (742 mg, 89% yield). ^1^H-NMR (400 MHz, CDCl_3_) δ 7.41–7.20 (m, 5H), 6.66 (dd, *J* = 15.9, 1.5 Hz, 1H), 6.27 (dt, *J* = 15.9, 6.4 Hz, 1H), 4.78 (dt, *J* = 6.5, 1.3 Hz, 2H), 3.37 (q, *J* = 7.3 Hz, 1H), 1.43 (s, 9H), 1.39 (d, *J* = 7.3 Hz, 3H) ppm; ^13^C-NMR (200 MHz, CD_3_OD) δ 172.55, 171.71, 138.47, 136.32, 130.47, 129.94, 128.43, 124.73, 83.65, 67.50, 28.91, 14.66 ppm; IR (KBr) 3,902, 3,734, 3,596, 3,566, 2,970, 2,348, 2,309, 1748, 1,543, 1,508, 1,456, 1,370, 1,216, 1,148, 968, 720, 691, 677, 648, 615 cm^−1^; HRMS (FAB) m/z: [M + H]^+^ Calcd for [C_17_H_23_O_4_]^+^([M + H]^+^) 291.1596; found 291.1597.

###### 4.1.1.3.5 1-(*tert*-Butyl) 3-(2,2-diphenylethyl) 2-methylmalonate (7)

α-methyl-malonate mono-*tert*-butyl ester (2, 500 mg, 2.87 mmol) was dissolved with 2,2-diphenylethanol (626 mg, 3.16 mmol) in 1,4-dioxane (10.13 mL) under argon air. 4-Dimethylaminopyridine (41.3 mg, 0.338 mmol) and 1-(3-dimethyl aminopropyl)-3-ethylcarbodiimide hydrochloride (1,100 mg, 5.74 mmol) was added to a stirred solution. After stirring for 15 h, water (15 mL) was added to the reaction mixture. The reaction mixture was extracted with dichlromethane (2 × 100 mL), washed with brine, dried over anhydrous MgSO_4_, filtered, and concentrated *in vacuo*. The residue was purified by column chromatography (silica gel, hexane: EtOAc = 40:1) to afford **7** (897 mg, 88% yield) as a colorless oil. ^1^H-NMR (400 MHz, CDCl_3_) δ 7.33–7.15 (m, 10H), 4.70–4.62 (m, 2H), 4.36 (td, *J* = 7.6, 2.2 Hz, 1H), 3.29–3.19 (m, 1H), 1.33 (s, 9H), 1.23 (dd, *J* = 7.2, 2.4 Hz, 3H) ppm; ^13^C-NMR (200 MHz, CD_3_OD) δ 172.67, 171.52, 143.37, 143.34, 130.42, 130.41, 130.08, 130.06, 128.64, 83.57, 69.21, 51.97, 28.83, 14.67 ppm; IR (KBr) 3,901, 3,734, 3,566, 2,970, 2,348, 2,309, 1747, 1,680, 1,543, 1,508, 1,489, 1,455, 1,370, 1,230, 1,145, 801, 700, 670, 648, 616 cm^−1^; HRMS (FAB) m/z: [M + H]^+^ Calcd for [C_22_H_27_O_4_]^+^([M + H]^+^) 355.1909; found 355.1911.

###### 4.1.1.3.6 1-Benzyl 3-(*tert*-butyl) (*S*)-sec-benzyl-2-methylmalonate (3e)

Following the procedure **(B)** from the substrate **3** (23 mg, 0.087 mmol) with benzyl bromide at 0°C, the compound **3e** was obtained as a white solid (mp 59°C, 25.0 mg, 81% yield). ^1^H-NMR (400 MHz, CDCl_3_) δ 7.44–6.99 (m, 10H), 5.18–5.12 (m, 2H), 3.28–3.11 (m, 2H), 1.32 (dt, *J* = 9.9, 3.2 Hz, 12H) ppm; ^13^C-NMR (200 MHz, CD_3_OD) δ 174.05, 173.08, 138.42, 137.92, 132.21, 130.66, 130.40, 130.30, 129.95, 128.71, 83.81, 68.81, 57.48, 42.90, 28.79, 21.10 ppm; IR (KBr) 3,902, 3,840, 3,734, 3,595, 3,566, 2,359, 2,348, 2,309, 1747, 1,646, 1,564, 1,543, 1,508, 1,488, 1,363, 1,230, 1,205, 803, 670, 648 cm^−1^; HRMS (FAB) m/z: [M + H]^+^ Calcd for [C_22_H_27_O_4_]^+^([M + H]^+^) 355.1909; found 355.1904. The enantioselectivity was determined by chiral HPLC analysis (DAICEL Chiralcel OJ-H, hexane: 2-propanol = 99.8 : 0.2, flow rate = 1.0 mL/min, 23°C, λ = 256 nm) retention time: minor isomer 27.93 min, major isomer 31.79 min, 77% ee, [α]^20^_D_ = +7.90 (*c* 1.0, CHCl_3_).

###### 4.1.1.3.7 1-(*tert*-Butyl) 3-phenethyl (*S*)-2-benzyl-2-methylmalonate (4e)

Following the procedure **(B)** from the substrate **4** (23 mg, 0.083 mmol) with benzyl bromide at 0°C, the compound **4e** was obtained as a colorless oil (26.4 mg, 86% yield). ^1^H-NMR (400 MHz, CDCl_3_) δ 7.33–7.14 (m, 8H), 7.07–7.00 (m, 2H), 4.39–4.23 (m, 2H), 3.18 (dd, *J* = 13.6, 1.8 Hz, 1H), 3.11 (dd, *J* = 13.7, 1.9 Hz, 1H), 2.93 (t, *J* = 7.2 Hz, 2H), 1.40 (s, 9H), 1.26 (d, *J* = 1.7 Hz, 3H) ppm; ^13^C-NMR (200 MHz, CD_3_OD) δ 174.28, 173.20, 140.12, 138.41, 132.17, 130.83, 130.36, 129.91, 128.68, 128.45, 83.78, 67.90, 57.56, 42.80, 36.69, 28.89, 21.04 ppm; IR (KBr) 3,902, 3,840, 3,734, 3,595, 3,566, 2,348, 2,309, 1747, 1,646, 1,564, 1,543, 1,508, 1,488, 1,373, 1,217, 772, 672, 649, 616 cm^−1^; HRMS (FAB) m/z: [M + H]^+^ Calcd for [C_23_H_29_O_4_]^+^([M + H]^+^) 369.2066; found 369.2069. The enantioselectivity was determined by chiral HPLC analysis (DAICEL Chiralcel OJ-H, hexane: 2-propanol = 99.5: 0.5, flow rate = 1.0 mL/min, 23°C, λ = 256 nm) retention time: major isomer 10.60 min, minor isomer 14.96 min, 60% ee, [α]^20^_D_ = +15.63 (*c* 1.0, CHCl_3_).

###### 4.1.1.3.8 1-(*tert*-Butyl) 3-(3-phenylpropyl) (*S*)-2-benzyl-2-methylmalonate (5e)

Following the procedure **(B)** from the substrate **5** (23 mg, 0.079 mmol) with benzyl bromide at 0°C, the compound **5e** was obtained as a colorless oil (12.4 mg, 41% yield). ^1^H-NMR (400 MHz, CDCl_3_) δ 7.32–7.09 (m, 10H), 4.21–4.04 (m, 2H), 3.19 (qd, *J* = 13.7, 2.3 Hz, 2H), 2.67 (td, *J* = 7.8, 2.2 Hz, 2H), 2.06–1.87 (m, 2H), 1.44 (d, *J* = 2.7 Hz, 9H), 1.31 (d, *J* = 2.4 Hz, 3H) ppm; ^13^C-NMR (200 MHz, CD_3_OD) δ 174.42, 173.27, 143.23, 138.50, 132.23, 130.29, 130.25, 129.96, 128.75, 127.88, 83.83, 66.42, 57.65, 42.95, 33.86, 32.22, 28.96, 21.19 ppm; IR (KBr) 3,902, 3,840, 3,757, 3,734, 3,566, 2,348, 2,309, 1747, 1,680, 1,646, 1,564, 1,543, 1,508, 1,488, 1,363, 1,218, 772, 671, 649 cm^−1^; HRMS (FAB) m/z: [M + H]^+^ Calcd for [C_24_H_31_O_4_]^+^([M + H]^+^) 383.2222; found 383.2229. The enantioselectivity was determined by chiral HPLC analysis (DAICEL Chiralcel OJ-H, hexane: 2-propanol = 99.5: 0.5, flow rate = 1.0 mL/min, 23°C, λ = 256 nm) retention time: minor isomer 14.350 min, minor isomer 26.143 min, 53% ee, [α]^20^_D_ = +19.62 (*c* 1.0, CHCl_3_).

###### 4.1.1.3.9 1-(*tert*-Butyl) 3-cinnamyl (*S*)-2-benzyl-2-methylmalonate (6e)

Following the procedure **(B)** from the substrate **6** (23 mg, 0.079 mmol) with benzyl bromide at 0°C, the compound **6e** was obtained as a colorless oil (28.8 mg, 96% yield). ^1^H-NMR (400 MHz, CDCl_3_) δ 7.42–7.09 (m, 10H), 6.65 (d, *J* = 15.8 Hz, 1H), 6.26 (dtd, *J* = 15.5, 6.5, 1.9 Hz, 1H), 4.76 (dt, *J* = 6.5, 1.6 Hz, 2H), 3.28–3.20 (m, 1H), 3.16 (d, *J* = 13.7 Hz, 1H), 1.41 (d, *J* = 1.9 Hz, 9H), 1.31 (d, *J* = 1.9 Hz, 3H) ppm; ^13^C-NMR (200 MHz, CD_3_OD) δ 174.07, 173.17, 138.44, 138.41, 136.80, 132.25, 130.50, 130.02, 129.95, 128.73, 128.48, 124.64, 83.84, 67.64, 57.53, 42.88, 28.89, 21.08 ppm; IR (KBr) 3,902, 3,840, 3,734, 3,595, 3,566, 2,348, 2,309, 1747, 1,680, 1,646, 1,564, 1,543, 1,508, 1,488, 1,363, 1,216, 677, 649 cm^−1^; HRMS (FAB) m/z: [M + H]^+^ Calcd for [C_24_H_29_O_4_]^+^([M + H]^+^) 381.2066; found 381.2063. The enantioselectivity was determined by chiral HPLC analysis (DAICEL Chiralpak AD-H, hexane: 2-propanol = 99.5: 0.5, flow rate = 1.0 mL/min, 23°C, λ = 240 nm) retention time: minor isomer 17.68 min, major isomer 23.51 min, 56% ee, [α]^20^_D_ = +7.02 (*c* 1.0, CHCl_3_).

###### 4.1.1.3.10 1-(*tert*-Butyl) 3-(2,2-diphenylethyl) (*S*)-2-allyl-2-methylmalonate (7a)

Following the procedure **(B)** from the substrate **7** (23 mg, 0.065 mmol) with allyl bromide at −40°C, the compound **7a** was obtained as a colorless oil (25.4 mg, 99% yield). ^1^H-NMR (400 MHz, CDCl_3_) δ 7.32–7.16 (m, 10H), 5.58–5.43 (m, 1H), 5.01–4.89 (m, 2H), 4.64 (dd, *J* = 7.5, 4.3 Hz, 2H), 4.35 (t, *J* = 7.5 Hz, 1H), 2.43 (d, *J* = 6.9 Hz, 2H), 1.30 (s, 9H), 1.19 (s, 3H) ppm; ^13^C-NMR (200 MHz, CD_3_OD) δ 172.20, 170.66, 140.95, 140.93, 132.70, 128.58, 128.53, 128.20, 128.19, 126.83, 126.75, 118.79, 81.50, 67.40, 54.01, 49.78, 39.99, 29.10, 27.72, 19.63, 14.04 ppm; IR (KBr) 3,902, 3,840, 3,734, 3,566, 2,969, 2,348, 2,309, 1745, 1,646, 1,564, 1,543, 1,508, 1,488, 1,364, 1,218, 772, 673, 648 cm^−1^; HRMS (FAB) m/z: [M + H]^+^ Calcd for [C_25_H_31_O_4_]^+^([M + H]^+^) 395.2222; found 395.2230. The enantioselectivity was determined by chiral HPLC analysis (DAICEL Chiralpak AD-H, hexane: 2-propanol = 800: 1, flow rate = 1.0 mL/min, 23 °C, λ = 220 nm) retention time: minor isomer 31.67 min, major isomer 33.74 min, 88% ee, [α]^20^_D_ = +7.73 (*c* 1.0, CHCl_3_).

###### 4.1.1.3.11 1-(*tert*-Butyl) 3-(2,2-diphenylethyl) (*S*)-2-methyl-2-(2-methylallyl)malonate (7b)

Following the procedure **(B)** from the substrate **7** (23 mg, 0.065 mmol) with 2-methylallyl bromide at −40°C, the compound **7b** was obtained as a colorless oil (25 mg, 94% yield). ^1^H-NMR (400 MHz, CDCl_3_) δ 7.32–7.21 (m, 10H), 4.75 (p, *J* = 1.6 Hz, 1H), 4.68 (dd, *J* = 11.1, 7.5 Hz, 1H), 4.62–4.53 (m, 2H), 4.34 (t, *J* = 7.5 Hz, 1H), 2.52 (d, *J* = 1.0 Hz, 2H), 1.30 (s, 9H), 1.24 (s, 2H), 1.21 (s, 3H) ppm; ^13^C-NMR (200 MHz, CDCl_3_) δ 172.64, 171.06, 141.00, 140.99, 140.95, 128.72, 128.58, 128.20, 128.19, 126.83, 115.18, 81.55, 67.58, 53.77, 49.74, 42.79, 29.10, 27.66, 23.32, 19.71 ppm; IR (KBr) 3,902, 3,840, 3,734, 3,566, 2,969, 2,348, 2,309, 1745, 1,646, 1,543, 1,508, 1,488, 1,364, 1,218, 772, 720, 672, 648, 617 cm^−1^; HRMS (FAB) m/z: [M + H]^+^ Calcd for [C_26_H_33_O_4_]^+^([M + H]^+^) 409.2379; found 409.2380. The enantioselectivity was determined by chiral HPLC analysis (DAICEL Chiralpak AD-H, hexane: 2-propanol = 800: 1, flow rate = 1.0 mL/min, 23°C, λ = 220 nm) retention time: minor isomer 34.71 min, major isomer 36.12 min, 90% ee, [α]^20^_D_ = +16.73 (*c* 1.0, CHCl_3_).

###### 4.1.1.3.12 1-(*tert*-Butyl) 3-(2,2-diphenylethyl) (*S*)-2-methyl-2-(prop-2-yn-1-yl)malonate (7c)

Following the procedure **(B)** from the substrate **7** (23 mg, 0.065 mmol) with progargyl bromide at −40°C, the compound **7c** was obtained as a colorless oil (23.5 mg, 92% yield). ^1^H-NMR (400 MHz, CDCl_3_) δ 7.34–7.12 (m, 10H), 4.70–4.57 (m, 2H), 4.35 (t, *J* = 7.5 Hz, 1H), 2.65–2.44 (m, 2H), 2.30 (t, *J* = 2.7 Hz, 1H), 1.28 (d, *J* = 2.3 Hz, 12H) ppm; ^13^C-NMR (200 MHz, CD_3_OD) δ 173.28, 171.72, 143.33, 143.27, 130.44, 130.10, 130.09, 128.68, 84.00, 80.84, 73.39, 69.54, 55.72, 51.93, 28.73, 27.35, 20.80 ppm; IR (KBr) 3,901, 3,840, 3,734, 3,566, 2,969, 2,348, 2,309, 1744, 1,646, 1,543, 1,508, 1,488, 1,364, 1,218, 772, 672, 649, 616 cm^−1^; HRMS (FAB) m/z: [M + H]^+^ Calcd for [C_25_H_29_O_4_]^+^([M + H]^+^) 393.2066; found 393.2063. The enantioselectivity was determined by chiral HPLC analysis (DAICEL Chiralpak AD-H, hexane: 2-propanol = 99: 1, flow rate = 1.0 mL/min, 23°C, λ = 256 nm) retention time: minor isomer 8.23 min, major isomer 8.87 min, 66% ee, [α]^20^_D_ = −3.32 (*c* 1.0, CHCl_3_).

###### 4.1.1.3.12 1-(*tert*-Butyl) 3-(2,2-diphenylethyl) (*S*)-2-cinnamyl-2-methylmalonate (7d)

Following the procedure **(B)** from the substrate **7** (23 mg, 0.065 mmol) with cinnamyl bromide at −40°C, the compound **7d** was obtained as a colorless oil (29.3 mg, 96% yield). ^1^H-NMR (400 MHz, CDCl_3_) δ 7.35–7.15 (m, 15H), 6.28 (d, *J* = 15.7 Hz, 1H), 5.92 (dt, *J* = 15.4, 7.5 Hz, 1H), 4.71–4.59 (m, 2H), 4.35 (t, *J* = 7.4 Hz, 1H), 2.62–2.55 (m, 2H), 1.31 (s, 9H), 1.25 (s, 3H) ppm; ^13^C-NMR (200 MHz, CD_3_OD) δ 172.18, 170.67, 140.98, 140.92, 137.15, 133.71, 128.58, 128.43, 128.20, 128.18, 127.25, 126.84, 126.19, 124.48, 81.60, 67.42, 54.46, 49.77, 39.32, 27.74, 19.94 ppm; IR (KBr) 3,902, 3,840, 3,734, 3,566, 2,969, 2,359, 2,348, 2,309, 1745, 1,646, 1,543, 1,508, 1,488, 1,363, 1,218, 772, 672, 649 cm^−1^; HRMS (FAB) m/z: [M + H]^+^ Calcd for [C_31_H_35_O_4_]^+^([M + H]^+^) 471.2535; found 471.2534. The enantioselectivity was determined by chiral HPLC analysis (DAICEL Chiralcel OD-H, hexane: 2-propanol = 99: 1, flow rate = 1.0 mL/min, 23°C, λ = 220 nm) retention time: minor isomer 8.09 min, major isomer 13.87 min, 86% ee, [α]^20^_D_ = +17.40 (*c* 1.0, CHCl_3_).

###### 4.1.1.3.13 1-(*tert*-Butyl) 3-(2,2-diphenylethyl) (*S*)-2-benzyl-2-methylmalonate (7e)

Following the procedure **(B)** from the substrate **7** (23 mg, 0.065 mmol) with benzyl bromide at −40°C, the compound **7e** was obtained as a colorless oil (21.7 mg, 75% yield). ^1^H-NMR (400 MHz, CDCl_3_) δ 7.34–7.11 (m, 13H), 6.97–6.90 (m, 2H), 4.71–4.55 (m, 2H), 4.33 (td, *J* = 7.5, 2.8 Hz, 1H), 3.13–3.00 (m, 2H), 1.31 (s, 9H), 1.15 (d, *J* = 3.2 Hz, 3H) ppm; ^13^C-NMR (200 MHz, CD_3_OD) δ 172.12, 170.79, 141.09, 140.94, 136.31, 130.24, 128.61, 128.59, 128.24, 128.17, 128.04, 126.88, 126.85, 126.70, 81.69, 67.62, 55.44, 49.72, 40.88, 31.92, 29.69, 27.71, 19.71, 14.12 ppm; IR (KBr) 3,902, 3,840, 3,734, 3,586, 3,566, 2,969, 2,348, 2,309, 1746, 1,680, 1,646, 1,564, 1,543, 1,508, 1,488, 1,363, 1,218, 772, 672, 649 cm^−1^; HRMS (FAB) m/z: [M + H]^+^ Calcd for [C_29_H_33_O_4_]^+^([M + H]^+^) 445.2379; found 445.2387. The enantioselectivity was determined by chiral HPLC analysis (DAICEL Chiralcel OJ-H, hexane: 2-propanol = 95 : 5, flow rate = 1.0 mL/min, 23°C, λ = 256 nm) retention time: minor isomer 10.32 min, major isomer 13.24 min, 95% ee, [α]^20^_D_ = +22.36 (*c* 1.0, CHCl_3_).

###### 4.1.1.3.14 1-(*tert*-Butyl) 3-(2,2-diphenylethyl) (*S*)-2-methyl-2-(4-methylbenzyl)malonate (7f)

Following the procedure **(B)** from the substrate **7** (23 mg, 0.065 mmol) with *para*-methyl benzyl bromide at −40°C, the compound **7f** was obtained as a white solid (mp 75 °C, 29.5 mg, 99% yield). ^1^H-NMR (400 MHz, CDCl_3_) δ 7.34–7.15 (m, 10H), 6.97 (d, *J* = 7.8 Hz, 2H), 6.80 (d, *J* = 8.0 Hz, 2H), 4.70–4.55 (m, 2H), 4.33 (t, *J* = 7.4 Hz, 1H), 3.07–2.94 (m, 2H), 2.27 (s, 3H), 1.31 (s, 9H), 1.14 (s, 3H) ppm; ^13^C-NMR (200 MHz, CDCl_3_) δ 172.17, 170.88, 141.12, 140.97, 136.20, 133.12, 130.09, 128.83, 128.75, 128.59, 128.24, 128.17, 126.87, 126.83, 81.62, 67.59, 55.45, 49.72, 44.18, 40.45, 27.72, 21.02, 19.66 ppm; IR (KBr) 3,902, 3,840, 3,734, 3,566, 2,969, 2,359, 2,348, 2,309, 1745, 1,646, 1,543, 1,508, 1,488, 1,363, 1,218, 772, 672, 648 cm^−1^; HRMS (FAB) m/z: [M + H]^+^ Calcd for [C_30_H_35_O_4_]^+^([M + H]^+^) 459.2535; found 459.2532. The enantioselectivity was determined by chiral HPLC analysis (DAICEL Chiralcel OD-H, hexane: 2-propanol = 200: 1, flow rate = 1.0 mL/min, 23°C, λ = 220 nm) retention time: minor isomer 20.44 min, major isomer 21.41 min, 95% ee, [α]^20^_D_ = +20.69 (*c* 1.0, CHCl_3_).

###### 4.1.1.3.15 1-(*tert*-Butyl) 3-(2,2-diphenylethyl) (*S*)-2-(3-methoxybenzyl)-2-methylmalonate (7g)

Following the procedure **(B)** from the substrate **7** (23 mg, 0.065 mmol) with 3-methoxybenzyl bromide at −40°C, the compound **7g** was obtained as a colorless oil (30.2 mg, 98% yield). ^1^H-NMR (400 MHz, CDCl_3_) δ 7.32–7.15 (m, 10H), 7.09 (t, *J* = 7.9 Hz, 1H), 6.76–6.69 (m, 1H), 6.62–6.56 (m, 1H), 6.55–6.49 (m, 1H), 4.68 (dd, *J* = 11.1, 7.4 Hz, 1H), 4.58 (dd, *J* = 11.1, 7.5 Hz, 1H), 4.32 (t, *J* = 7.5 Hz, 1H), 3.73 (s, 3H), 3.04 (t, *J* = 2.4 Hz, 2H), 1.30 (s, 9H), 1.15 (s, 3H) ppm; ^13^C-NMR (200 MHz, CDCl_3_) δ 172.15, 170.74, 159.27, 141.03, 140.94, 137.88, 128.98, 128.59, 128.58, 128.21, 128.18, 126.86, 126.83, 122.64, 116.17, 111.98, 81.69, 67.58, 55.40, 55.09, 49.72, 40.93, 27.70, 19.75 ppm; IR (KBr) 3,902, 3,840, 3,734, 3,566, 2,969, 2,359, 2,348, 2,309, 1744, 1,602, 1,543, 1,508, 1,488, 1,456, 1,365, 1,218, 1,112, 772, 672, 648 cm^−1^; HRMS (FAB) m/z: [M + H]^+^ Calcd for [C_30_H_34_O_5_]^+^([M + H]^+^) 474.2406; found 474.2403. The enantioselectivity was determined by chiral HPLC analysis (DAICEL Chiralcel OJ-H, hexane: 2-propanol = 99: 1, flow rate = 1.0 mL/min, 23°C, λ = 256 nm) retention time: minor isomer 41.17 min, major isomer 53.38 min, 95% ee, [α]^20^_D_ = +11.41 (*c* 1.0, CHCl_3_).

###### 4.1.1.3.16 1-(*tert*-Butyl) 3-(2,2-diphenylethyl) (*S*)-2-(4-fluorobenzyl)-2-methylmalonate (7h)

Following the procedure **(B)** from the substrate **7** (23 mg, 0.065 mmol) with *para*-fluoro benzyl bromide at −40°C, the compound **7h** was obtained as a colorless oil (29.8 mg, 99% yield). ^1^H-NMR (400 MHz, CD_3_OD) δ 7.32–7.08 (m, 10H), 7.00–6.83 (m, 4H), 4.61 (qd, *J* = 11.1, 7.4 Hz, 2H), 4.33 (t, *J* = 7.4 Hz, 1H), 3.04–2.92 (m, 2H), 1.30 (s, 9H), 1.09 (s, 3H) ppm; ^13^C-NMR (200 MHz, CD_3_OD) δ 174.10, 172.94, 164.74, 163.53, 143.49, 143.34, 134.30, 134.29, 133.89, 133.85, 131.72, 130.47, 130.16, 130.09, 128.74, 128.72, 116.57, 116.46, 83.89, 69.63, 57.58, 51.95, 41.89, 28.84, 21.03 ppm; IR (KBr) 3,901, 3,840, 3,734, 3,647, 3,565, 2,979, 2,348, 2,319, 1729, 1,604, 1,509, 1,455, 1,369, 1,279, 1,222, 1,159, 1,112, 985, 844, 772, 700 cm^−1^; HRMS (FAB) m/z: [M + H]^+^ Calcd for [C_29_H_32_FO_4_]^+^([M + H]^+^) 463.2285; found 463.2280. The enantioselectivity was determined by chiral HPLC analysis (DAICEL Chiralcel OJ-H, hexane: 2-propanol = 99: 1, flow rate = 1.0 mL/min, 23°C, λ = 220 nm) retention time: minor isomer 20.92 min, major isomer 30.37 min, 98% ee, [α]^20^_D_ = +6.47 (*c* 1.0, CHCl_3_).

###### 4.1.1.3.17 1-(*tert*-Butyl) 3-(2,2-diphenylethyl) (*S*)-2-(4-chlorobenzyl)-2-methylmalonate (7i)

Following the procedure **(B)** from the substrate **7** (23 mg, 0.065 mmol) with *para*-chloro benzyl bromide at −40°C, the compound **7i** was obtained as a colorless oil (29.6 mg, 98% yield). ^1^H-NMR (400 MHz, CD_3_OD) δ 7.31–7.19 (m, 8H), 7.22–7.11 (m, 4H), 6.93–6.85 (m, 2H), 4.60 (qd, *J* = 11.1, 7.4 Hz, 2H), 4.31 (t, *J* = 7.4 Hz, 1H), 3.04–2.91 (m, 2H), 1.30 (s, 9H), 1.09 (s, 3H) ppm; ^13^C-NMR (200 MHz, CD_3_OD) δ 173.99, 172.85, 143.47, 143.32, 137.18, 134.61, 133.74, 130.46, 130.15, 130.08, 129.96, 128.74, 128.71, 83.95, 69.65, 57.50, 42.05, 28.83, 21.08 ppm; IR (KBr) 3,901, 3,841, 3,734, 3,565, 2,978, 2,348, 2,310, 1729, 1,599, 1,508, 1,490, 1,455, 1,369, 1,250, 1,156, 1,111, 1,016, 846, 772, 700 cm^-1^; HRMS (FAB) m/z: [M + H]^+^ Calcd for [C_29_H_32_ClO_4_]^+^([M + H]^+^) 479.1989; found 479.1987. The enantioselectivity was determined by chiral HPLC analysis (DAICEL Chiralpak AD-H, hexane: 2-propanol = 200: 1, flow rate = 1.0 mL/min, 23°C, λ = 220 nm) retention time: minor isomer 22.42 min, major isomer 31.40 min, 99% ee, [α]^20^_D_ = +20.18 (*c* 1.0, CHCl_3_).

###### 4.1.1.3.18 1-(*tert*-Butyl) 3-(2,2-diphenylethyl) (*S*)-2-(4-bromobenzyl)-2-methylmalonate (7j)

Following the procedure **(B)** from the substrate **7** (23 mg, 0.065 mmol) with *para*-bromo benzyl bromide at −40°C, the compound **7j** was obtained as a colorless oil (33.3 mg, 98% yield). ^1^H-NMR (400 MHz, CD_3_OD) δ 7.32–7.20 (m, 10H), 7.23–7.14 (m, 2H), 6.88–6.80 (m, 2H), 4.68–4.53 (m, 2H), 4.31 (t, *J* = 7.4 Hz, 1H), 2.96 (td, *J* = 13.8, 13.6, 9.6 Hz, 2H), 1.30 (s, 9H), 1.10 (s, 3H) ppm; ^13^C-NMR (200 MHz, CD_3_OD) δ 173.98, 172.85, 143.48, 143.32, 137.68, 134.11, 132.99, 130.47, 130.15, 130.09, 128.74, 128.72, 122.59, 83.97, 69.65, 57.46, 51.93, 42.13, 28.83, 21.09 ppm; IR (KBr) 3,901, 3,840, 3,734, 3,647, 3,565, 2,978, 2,348, 2,310, 1729, 1,601, 1,508, 1,488, 1,456, 1,369, 1,251, 1,156, 1,112, 1,012, 845, 772, 700 cm^−1^; HRMS (FAB) m/z: [M + H]^+^ Calcd for [C_29_H_32_BrO_4_]^+^([M + H]^+^) 523.1484; found 523.1479. The enantioselectivity was determined by chiral HPLC analysis (DAICEL Chiralcel OD-H, hexane: 2-propanol = 200: 1, flow rate = 1.0 mL/min, 23°C, λ = 220 nm) retention time: minor isomer 21.52 min, major isomer 23.18 min, 95% ee, [α]^20^_D_ = +15.94 (*c* 1.0, CHCl_3_).

###### 4.1.1.3.19 1-(*tert*-Butyl) 3-(2,2-diphenylethyl) (*S*)-2-methyl-2-(4-nitrobenzyl)malonate (7k)

Following the procedure **(B)** from the substrate **7** (23 mg, 0.065 mmol) with *para*-nitro benzyl bromide at −40°C, the compound **7k** was obtained as a colorless oil (31.5 mg, 99% yield). ^1^H-NMR (400 MHz, CD_3_OD) δ 8.05–7.97 (m, 2H), 7.30–7.10 (m, 12H), 4.69–4.56 (m, 2H), 4.33 (t, *J* = 7.3 Hz, 1H), 3.18–3.04 (m, 2H), 1.31 (s, 9H), 1.13 (s, 3H) ppm; ^13^C-NMR (200 MHz, CD_3_OD) δ 173.64, 172.50, 149.17, 146.45, 143.42, 143.28, 133.28, 130.47, 130.46, 130.15, 130.08, 128.76, 128.73, 124.88, 84.21, 69.79, 57.40, 51.90, 42.39, 28.83, 21.15 ppm; IR (KBr) 3,901, 3,841, 3,734, 3,647, 3,565, 2,930, 2,348, 2,310, 1747, 1729, 1,680, 1,646, 1,605, 1,518, 1,489, 1,348, 1,217, 1,115, 772, 700 cm^−1^; HRMS (FAB) m/z: [M + H]^+^ Calcd for [C_29_H_32_NO_6_]^+^([M + H]^+^) 490.2230; found 490.2226. The enantioselectivity was determined by chiral HPLC analysis (DAICEL Chiralpak AD-H, hexane: 2-propanol = 99: 1, flow rate = 1.0 mL/min, 23°C, λ = 256 nm) retention time: minor isomer 25.02 min, major isomer 27.35 min, 92% ee, [α]^20^_D_ = +26.22 (*c* 1.0, CHCl_3_).

###### 4.1.1.3.20 1-(*tert*-Butyl) 3-(2,2-dipsecenylethyl) (*S*)-2-methyl-2-(naphthalen-2-ylmethyl)malonate (7L)

Following the procedure **(B)** from the substrate **7** (23 mg, 0.065 mmol) with 2-naphthylmethyl bromide at −40°C, the compound **7L** was obtained as a colorless oil (31.4 mg, 97% yield). ^1^H-NMR (400 MHz, CDCl_3_) δ 7.77 (t, *J* = 4.8 Hz, 1H), 7.69 (t, *J* = 4.8 Hz, 1H), 7.66 (d, *J* = 8.7 Hz, 1H), 7.42 (t, *J* = 4.8 Hz, 3H), 7.28–7.26 (m, 3H), 7.19 (dd, *J* = 7.8, 3.2 Hz, 6H), 7.08 (d, *J* = 6.9 Hz, 1H), 4.64 (ddd, *J* = 33.3, 11.1, 7.4 Hz, 2H), 4.30 (t, *J* = 7.5 Hz, 1H), 3.24 (d, *J* = 3.2 Hz, 2H), 1.32 (s, 9H), 1.19 (s, 3H) ppm; ^13^C-NMR (100 MHz, CDCl_3_) δ 172.25, 170.89, 141.16, 140.99, 134.05, 133.32, 132.42, 129.02, 128.68, 128.64, 128.32, 128.27, 127.69, 127.64, 127.61, 126.94, 125.98, 125.62, 81.86, 67.73, 55.74, 49.79, 41.14, 27.82, 19.94 ppm; IR (KBr) 3,902, 3,840, 3,734, 3,566, 2,969, 2,348, 2,309, 1745, 1,646, 1,543, 1,508, 1,488, 1,364, 1,218, 772, 673, 648, 617 cm^−1^; HRMS (FAB) m/z: [M + H]^+^ Calcd for [C_33_H_34_O_4_]^+^([M + H]^+^) 494.2457; found 464.2462. The enantioselectivity was determined by chiral HPLC analysis (DAICEL Chiralcel OD-H, hexane: 2-propanol = 99: 1, flow rate = 1.0 mL/min, 23°C, λ = 220 nm) retention time: minor isomer 9.38 min, major isomer 11.83 min, 91% ee, [α]^20^_D_ = +16.20 (*c* 1.0, CHCl_3_).

###### 4.1.1.3.21 Successive PTC α,α-alkylations of *tert*-butyl (2,2-diphenylethyl) malonate

Iodomethane (9 μL, 0.11 mmol) was added to a solution of tert-butyl (2,2-diphenylethyl) malonate, (34 mg, 0.1 mmol) and (*S*,*S*)-3,4,5-trifluorophenyl-NAS bromide (**8**, 4.7 mg, 0.005 mmol) in toluene (1 mL). At −40°C, aq. 50% KOH (78 μL, 0.7 mmol) was added to the reaction mixture. After stirring for 8 h, benzyl bromide (60 μL, 0.50 mmol) was added to the reaction mixture. After completion of the reaction, the reaction mixture was diluted with ethyl acetate (10 mL), washed with brine (5 mL x 2), dried over anhydrous MgSO_4_, filtered, and concentrated *in vacuo*. The residue was purified by column chromatography (silica gel 230–400 mesh, hexane: EtOAc = 20: 1) to afford **7e** (39 mg, 85% yield). The spectral data were exactly same as **7e**. The enantioselectivity was determined by chiral HPLC analysis (DAICEL Chiralcel OJ-H, hexane: 2-propanol = 95: 5, flow rate = 1.0 mL/min, 23°C, λ = 256 nm) retention time: minor isomer 10.23 min, major isomer 13.15 min, 95% ee.

###### 4.1.1.3.22 (*R*)-2-Benzyl-3-(2,2-diphenylethoxy)-2-methyl-3-oxopropanoic acid (9e)

To the solution of **7e** (30 mg, 0.067 mmol) in dichloromethane (0.8 mL) was added trifluoroacetic acid (0.2 mL) at 0°C. After stirring for 12 h, the reaction mixture was concentrated *in vacuo* to give **9e** (24.2 mg, 93% yield) as a pale yellow oil. ^1^H-NMR (400 MHz, CD_3_OD) δ 7.34–7.08 (m, 13H), 6.94–6.85 (m, 2H), 4.69–4.54 (m, 2H), 4.33 (t, *J* = 7.3 Hz, 1H), 3.08–2.96 (m, 2H), 1.09 (s, 3H) ppm; ^13^C-NMR (200 MHz, CD_3_OD) δ 175.79, 174.23, 143.55, 143.41, 143.38, 143.33, 138.38, 132.07, 130.38, 130.36, 130.21, 130.13, 130.08, 130.04, 129.92, 128.65, 128.61, 69.67, 56.86, 51.80, 42.80, 20.78 ppm; IR (KBr) 3,840, 3,647, 3,062, 3,029, 2,927, 2,348, 2,320, 1713, 1,647, 1,601, 1,495, 1,453, 1,379, 1,217, 1,116, 1,030, 981, 754, 738, 700, 635 cm^−1^; HRMS (FAB) m/z: [M + H]^+^ Calcd for [C_25_H_25_O_4_]^+^([M + H]^+^) 389.1753; found 389.1747. [α]^20^_D_ = +2.47 (*c* 1.0, CHCl_3_).

###### 4.1.1.3.23 (*S*)-2-Benzyl-3-(*tert*-butoxy)-2-methyl-3-oxopropanoic acid (10e)

Compound **7e** (30 mg, 0.067 mmol) was dissolved in 1N KOH methanol solution (1 mL). After stirring for 12 h at room temperature, the reaction mixture was concentrated *in vacuo*, diluted with Ethyl acetate, extracted with water and acidified with aqueous 1N HCl. Then aqueous layer was extracted with dichloromethane (10 mL x 5). Organic extract was dried over anhydrous MgSO_4_, filtered, and concentrated *in vacuo* to give **10e** (16.8 mg, 94% yield) as a white solid (mp 73.4 °C). ^1^H-NMR (400 MHz, (CD_3_)_2_SO) δ 7.36–7.06 (m, 5H), 3.07–2.94 (m, 2H), 1.35 (s, 9H), 1.10 (s, 3H) ppm; ^13^C-NMR (200 MHz, (CD_3_)_2_SO) δ 173.04, 170.56, 136.53, 130.15, 128.48, 127.96, 126.64, 80.79, 54.52, 40.34, 29.77, 27.46, 19.33 ppm; IR (KBr) 3,840, 3,031, 2,980, 2,929, 2,350, 1712, 1,605, 1,496, 1,455, 1,395, 1,370, 1,255, 1,219, 1,155, 1,119, 940, 847, 772, 700, 673, 635 cm^−1^; HRMS (FAB) m/z: [M + H]^+^ Calcd for [C_15_H_21_O_4_]^+^([M + H]^+^) 265.1440; found 265.1430. [α]^20^_D_ = +3.73 (*c* 1.0, CHCl_3_).

###### 4.1.1.3.24 (*S*)-3-(*tert*-Butoxy)-2-(4-chlorobenzyl)-2-methyl-3-oxopropanoic acid (10i)

Compound **7i** (90 mg, 0.188 mmol) was dissolved in 1N KOH methanol solution (3 mL). After stirring for 12 h at room temperature, the reaction mixture was concentrated *in vacuo*, diluted with Ethyl acetate, extracted with water and acidified with aqueous 1N HCl. Then aqueous layer was extracted with dichloromethane (20 mL x 5). Organic extract was dried over anhydrous MgSO_4_, filtered, and concentrated *in vacuo* to give **10i** (50.5 mg, 94% yield) as a white solid (mp 133°C). ^1^H-NMR (400 MHz, CD_2_Cl_2_) δ 7.27–7.19 (m, 2H), 7.14–7.06 (m, 2H), 3.22 (d, *J* = 13.7 Hz, 1H), 3.06 (d, *J* = 13.7 Hz, 1H), 1.43 (s, 9H), 1.39 (s, 3H) ppm; ^13^C-NMR (200 MHz, CD_2_Cl_2_) δ 173.08, 173.04, 135.32, 133.28, 131.75, 128.73, 84.20, 54.86, 41.99, 31.00, 27.91, 21.98 ppm; IR (KBr) 3,211, 3,000, 2,937, 1756, 1,697, 1,488, 1,374, 1,297, 1,253, 1,179, 1,159, 1,130, 1,109, 1,015, 842, 820, 773, 747, 682, 639 cm^−1^; HRMS (FAB) m/z: [M + H]^+^ Calcd for [C_15_H_20_ClO_4_]^+^([M + H]^+^) 299.1050; found 299.1056. [α]^20^_D_ = + 10.17 (*c* 1.0, CHCl_3_).

## Data Availability

The original contributions presented in the study are included in the article/Supplementary Material, CCDC 2243164 contains the supplementary crystallographic data for **10i** and the data can be obtained free of charge from The Cambridge Crystallographic Data Centre via www.ccdc.cam.ac.uk/data_request/cif, further inquiries can be directed to the corresponding authors.
