# Supplementary material for: Synthesis of chiral malonates by α-alkylation of 2,2-diphenylethyl tert-butyl malonates via enantioselective phase-transfer catalysis
Source: Front Chem. 2023 Jun 8;11:1205661. doi: 10.3389/fchem.2023.1205661 (PMC10285525; doi:10.3389/fchem.2023.1205661)
Supplement: Supplementary file 1 [file DataSheet1.pdf]

Supporting Information

Synthesis of Chiral Malonates by α-Alkylation of 2,2-Diphenylethyl tert-Butyl Malonates via Enantioselective Phase-Transfer Catalysis

Zhibin Guo,[a] Daehyun Oh,[a] Min Sagong, Jewon Yang, Geumwoo Lee,* and Hyeung-geun Park*

Research Institute of Pharmaceutical Sciences and College of Pharmacy, Seoul National University, Seoul 151-742, Korea.

hgpk@snu.ac.kr

List of Contents

(1) ^{1}H & ^{13}C NMR Spectra -- S2

(2) Chiral HPLC Chromatogram --- S52

(3) X-ray Crystallographic data-- S68

(1) ^{1}H & ^{13}C NMR Spectra

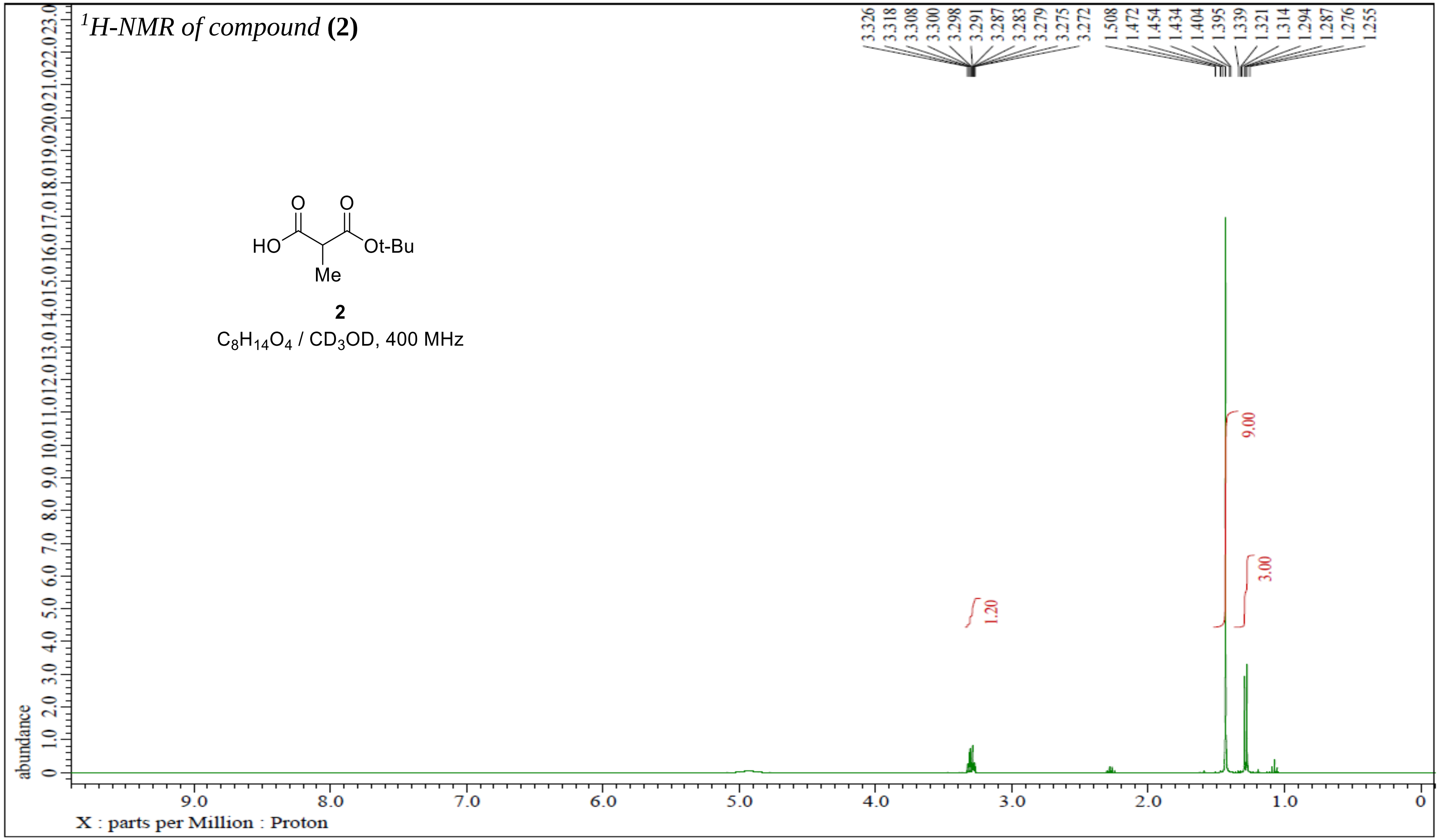

^{13}C-NMR of compound **(2)**

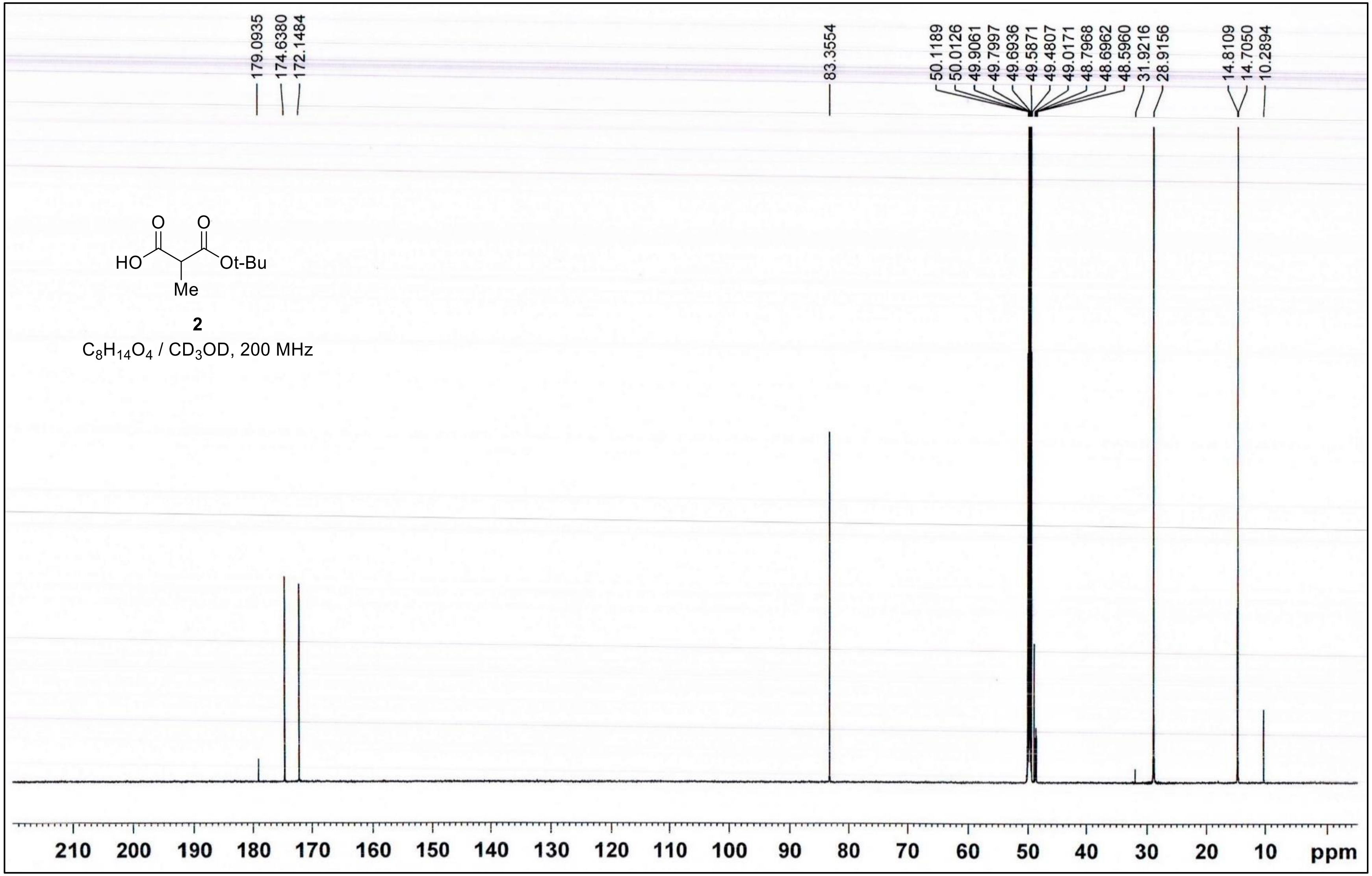

^{1}H-NMR of compound **(6)**

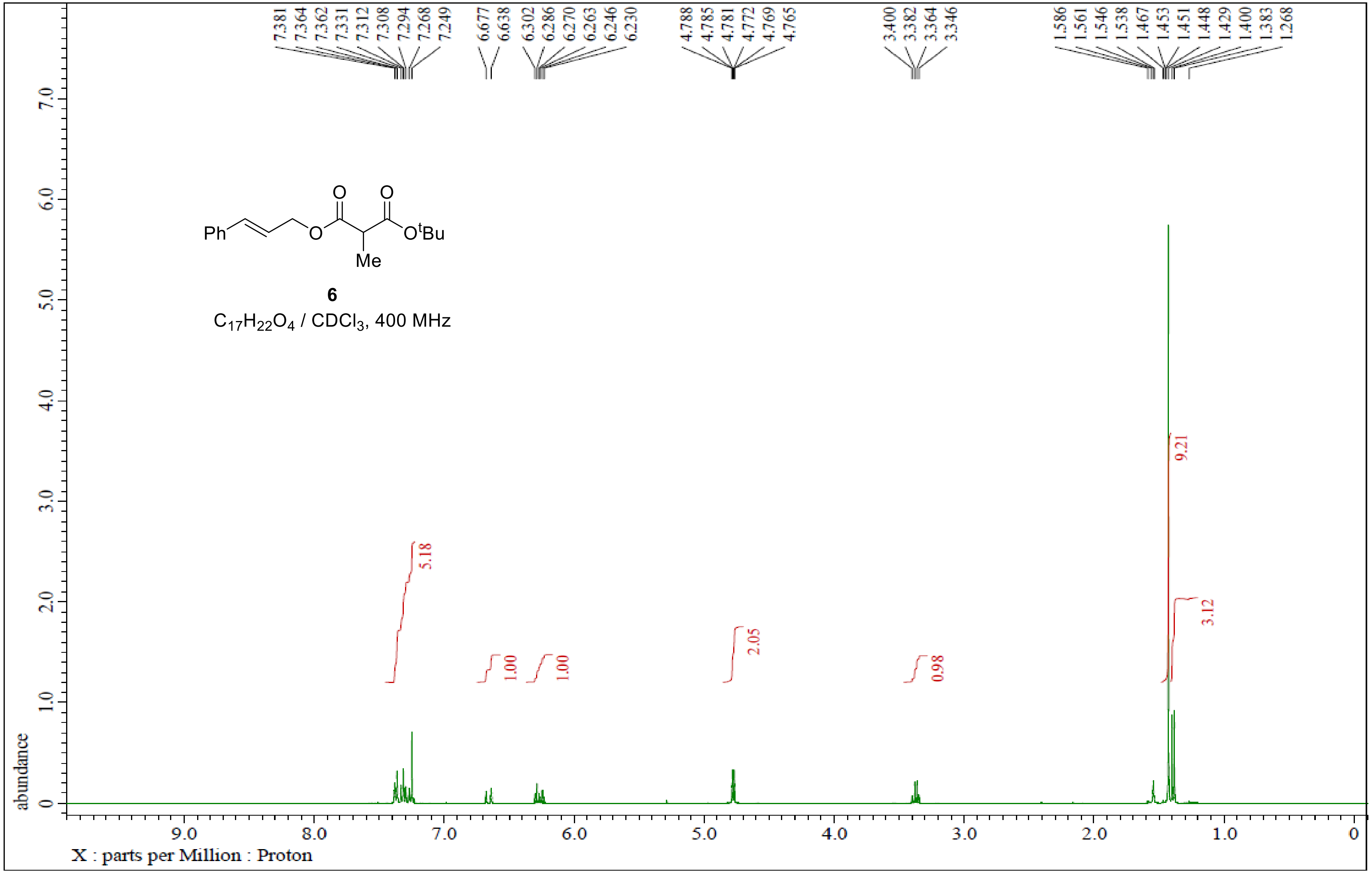

^{13}*C-NMR of compound* **(6)**

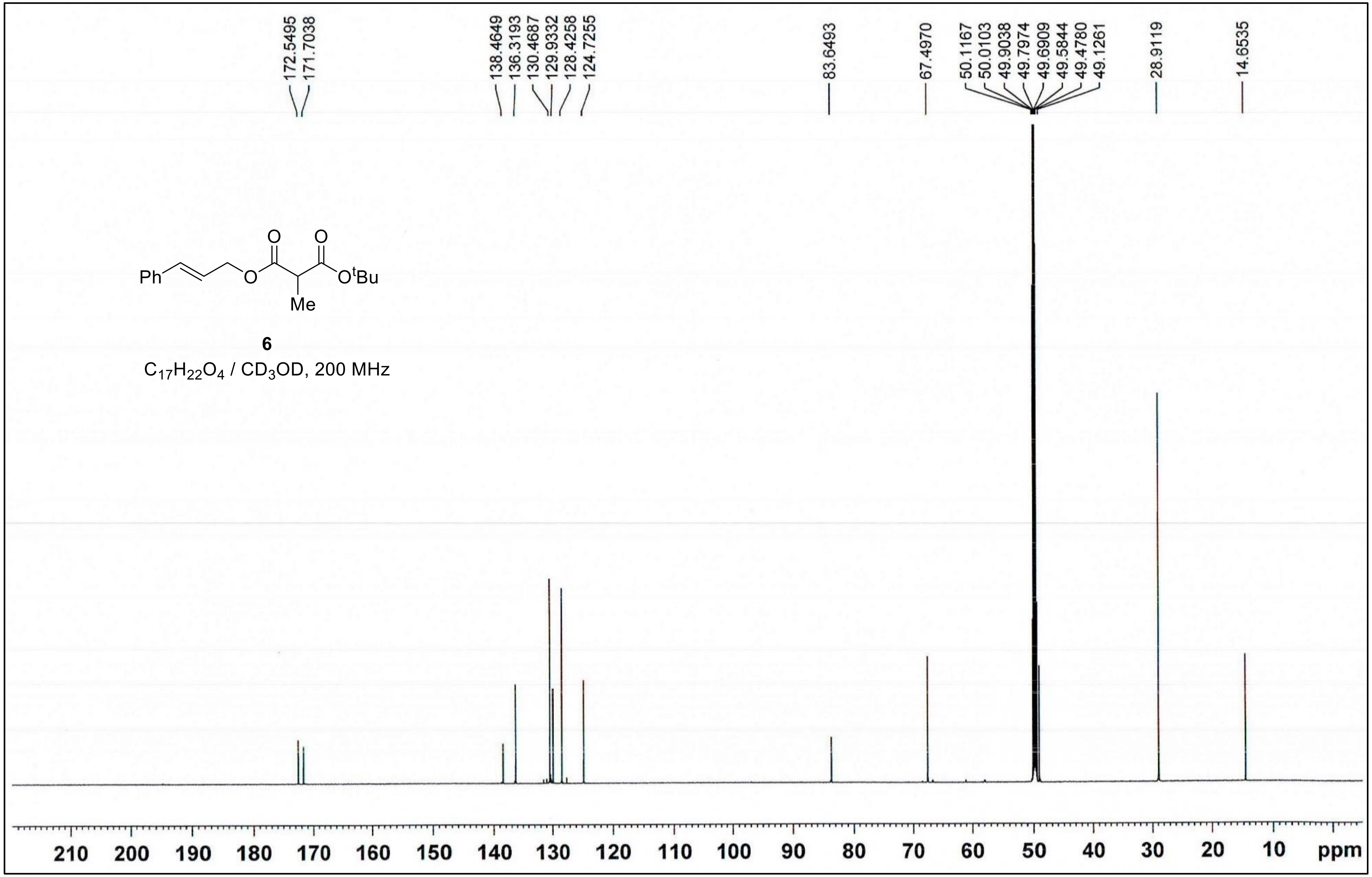

^{1}H-NMR of compound **(3)**

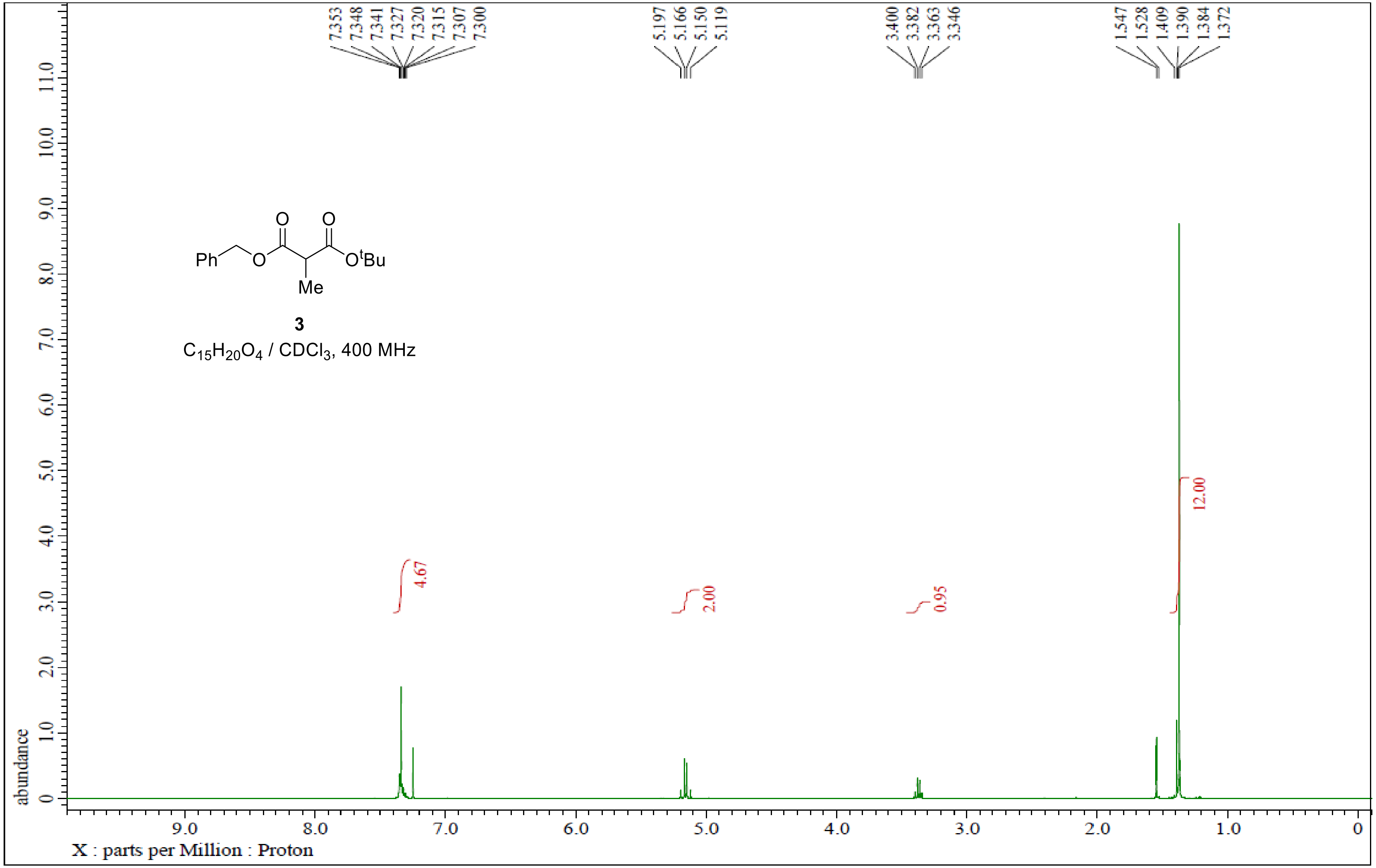

[13]*C-NMR of compound* **(3)**

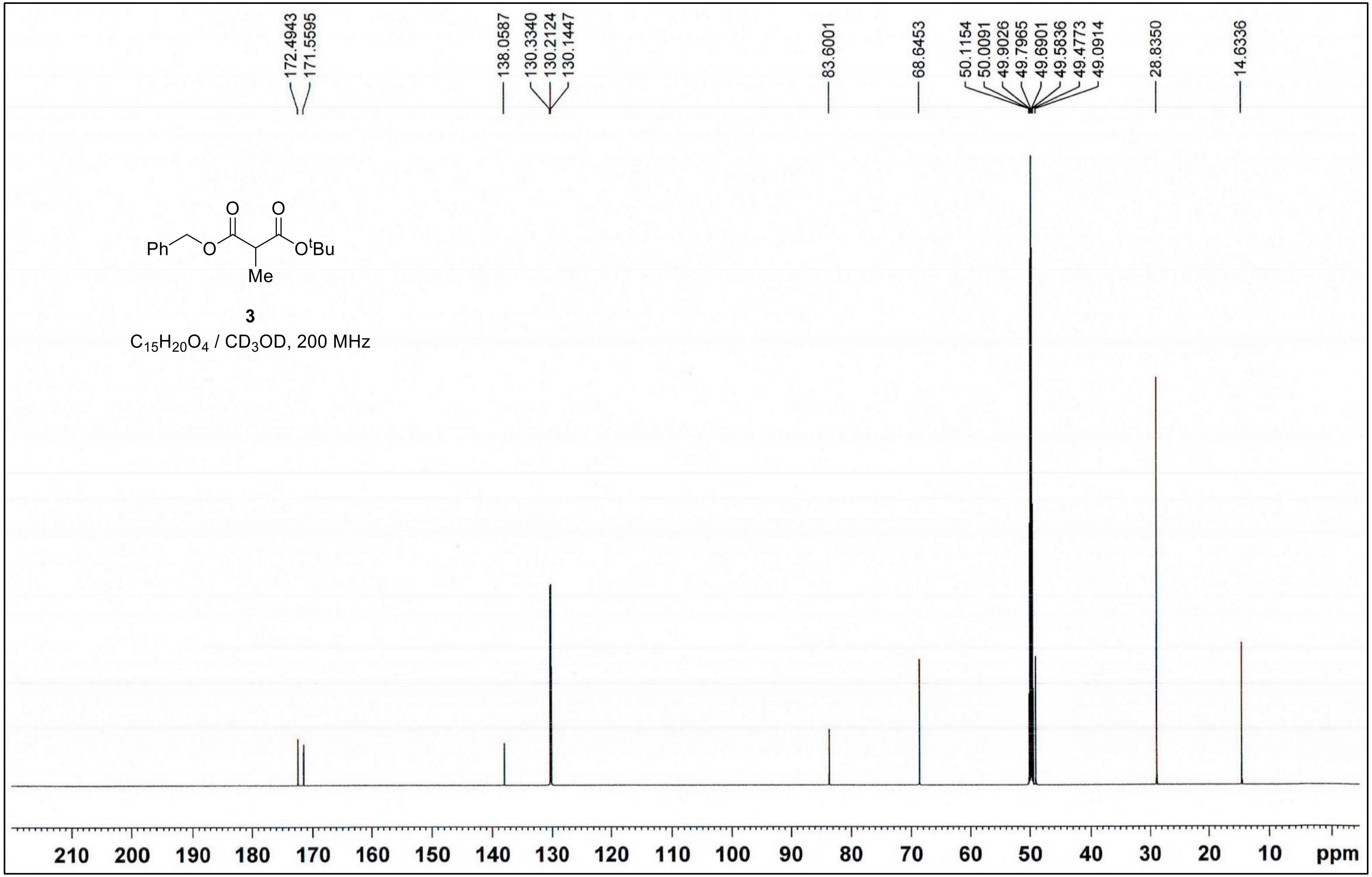

^{1}*H-NMR of compound* **(4)**

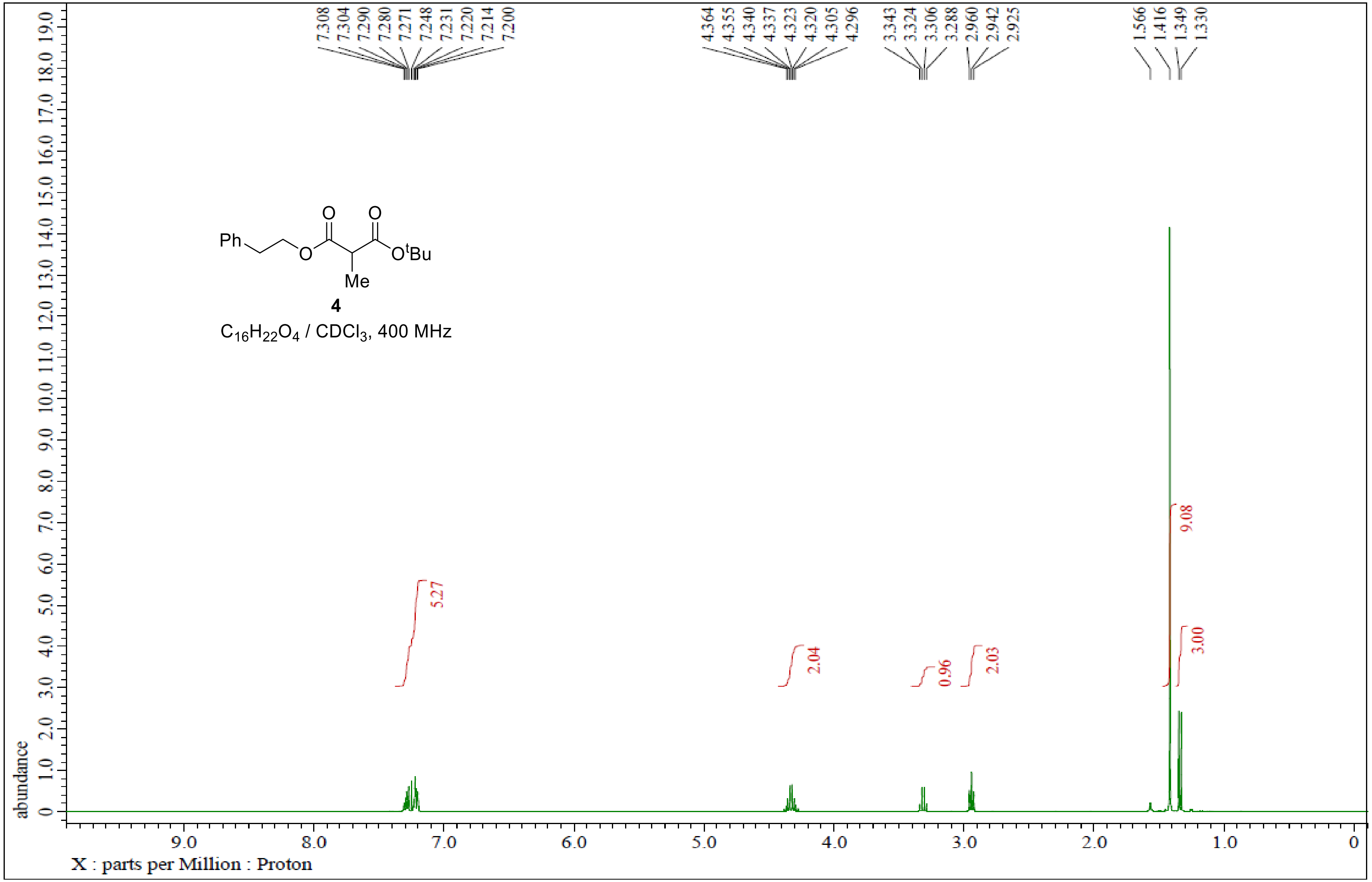

^{13}C-NMR of compound **(4)**

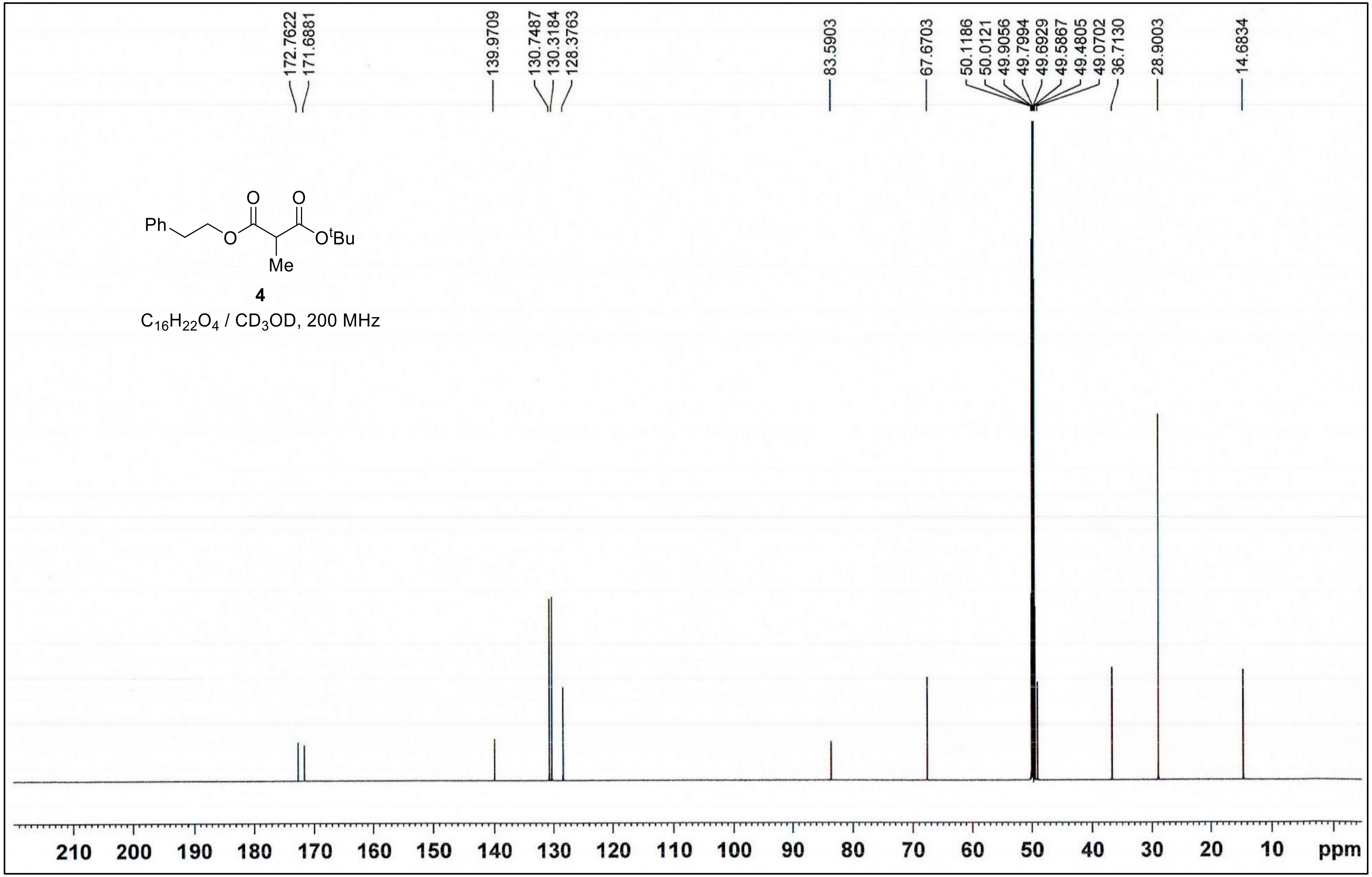

^{1}H-NMR of compound **(5)**

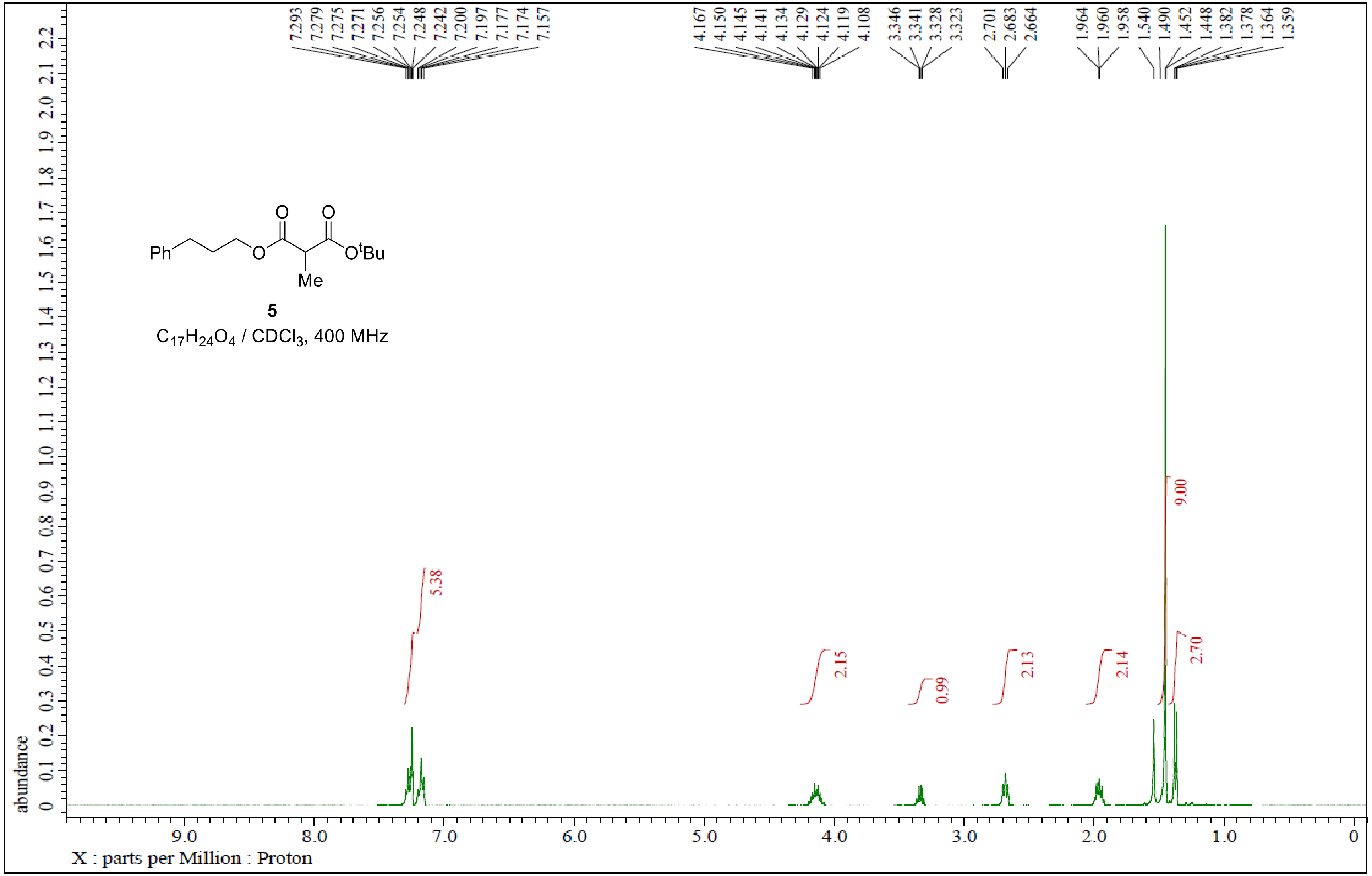

^{13}*C-NMR of compound* **(5)**

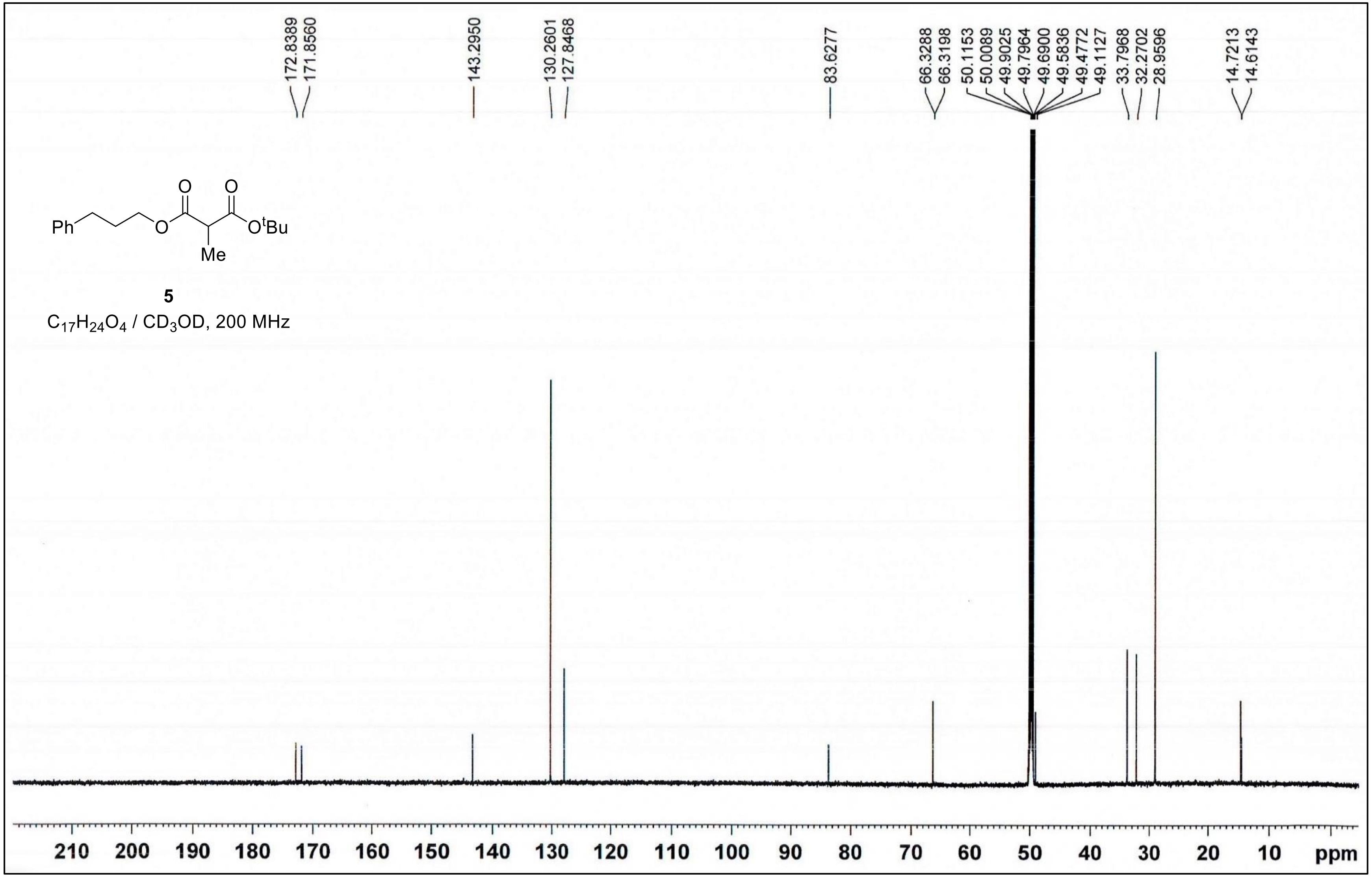

[1]*H-NMR of compound* **(7)**

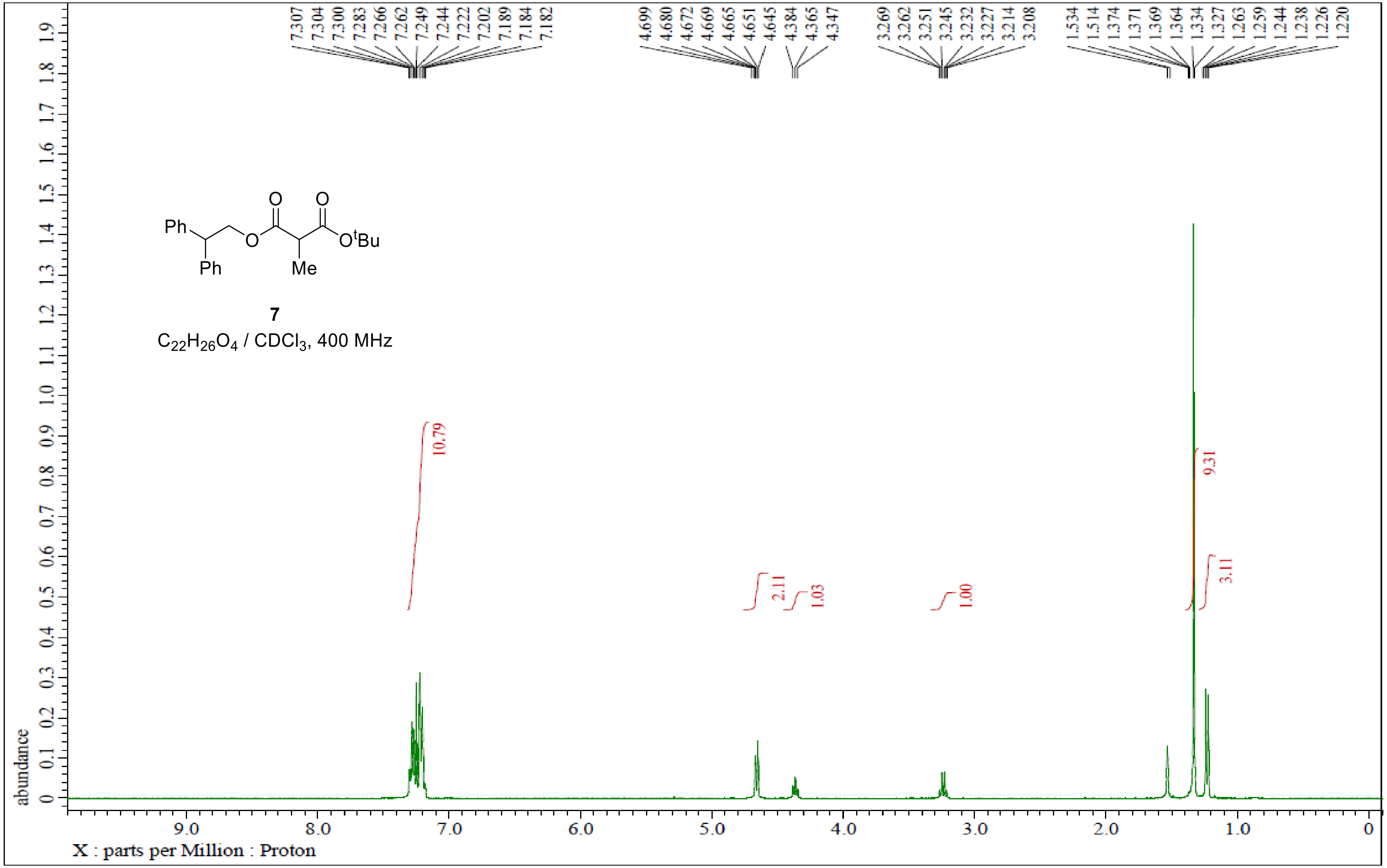

^{13}*C-NMR of compound* **(7)**

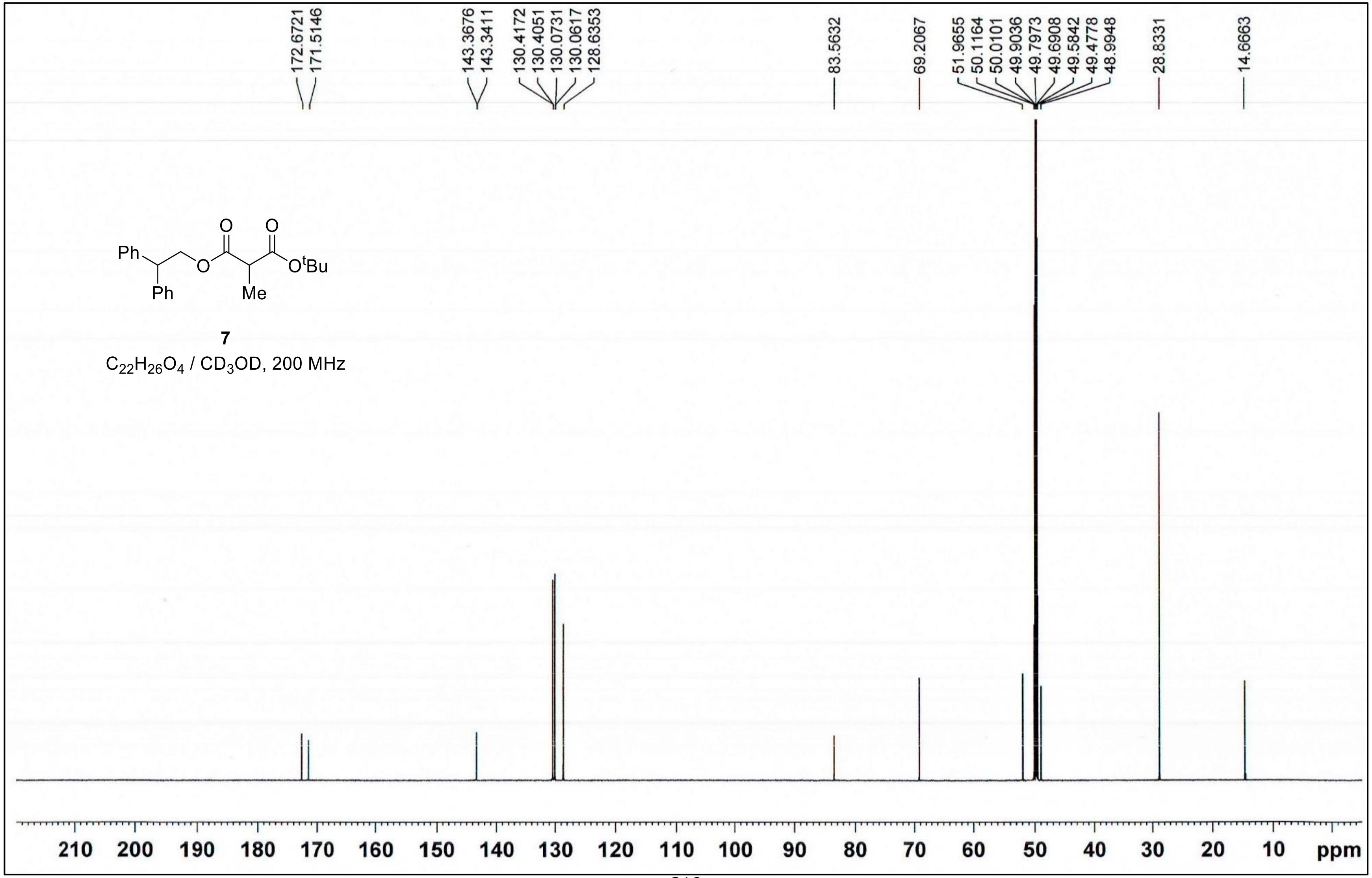

^{1}*H-NMR of compound* **(3e)**

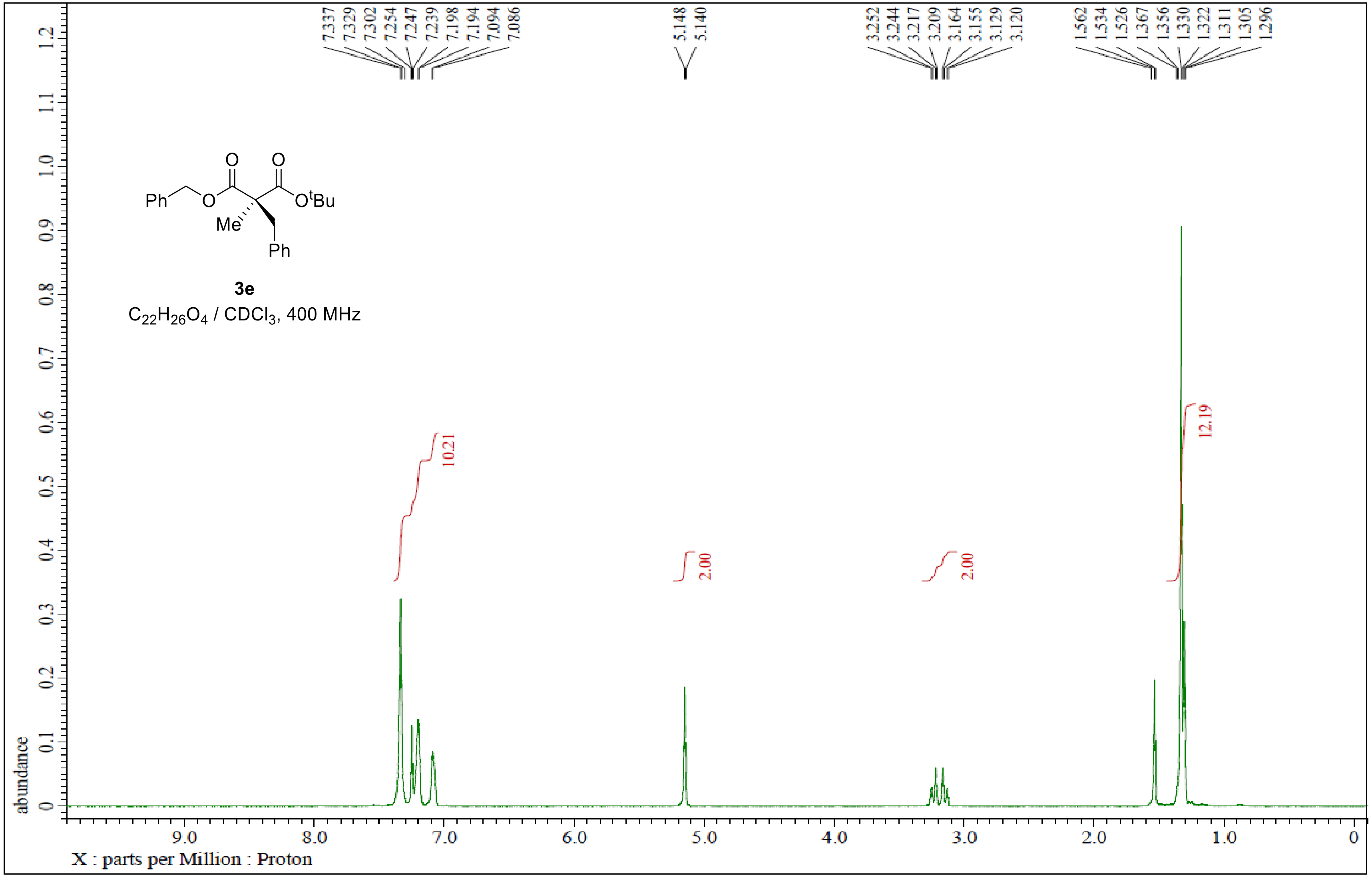

^{13}C-NMR of compound **(3e)**

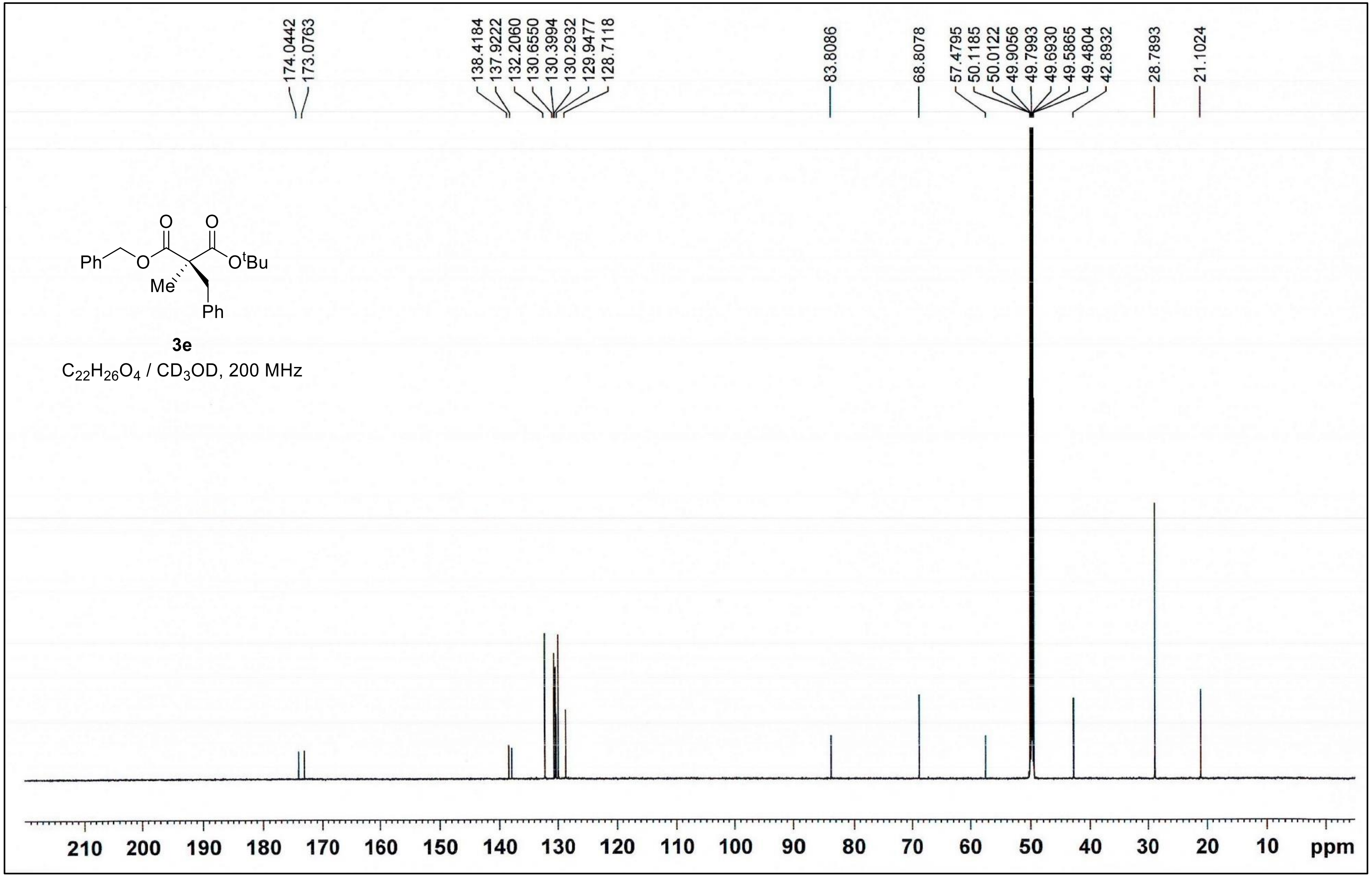

^{1}H-NMR of compound **(4e)**

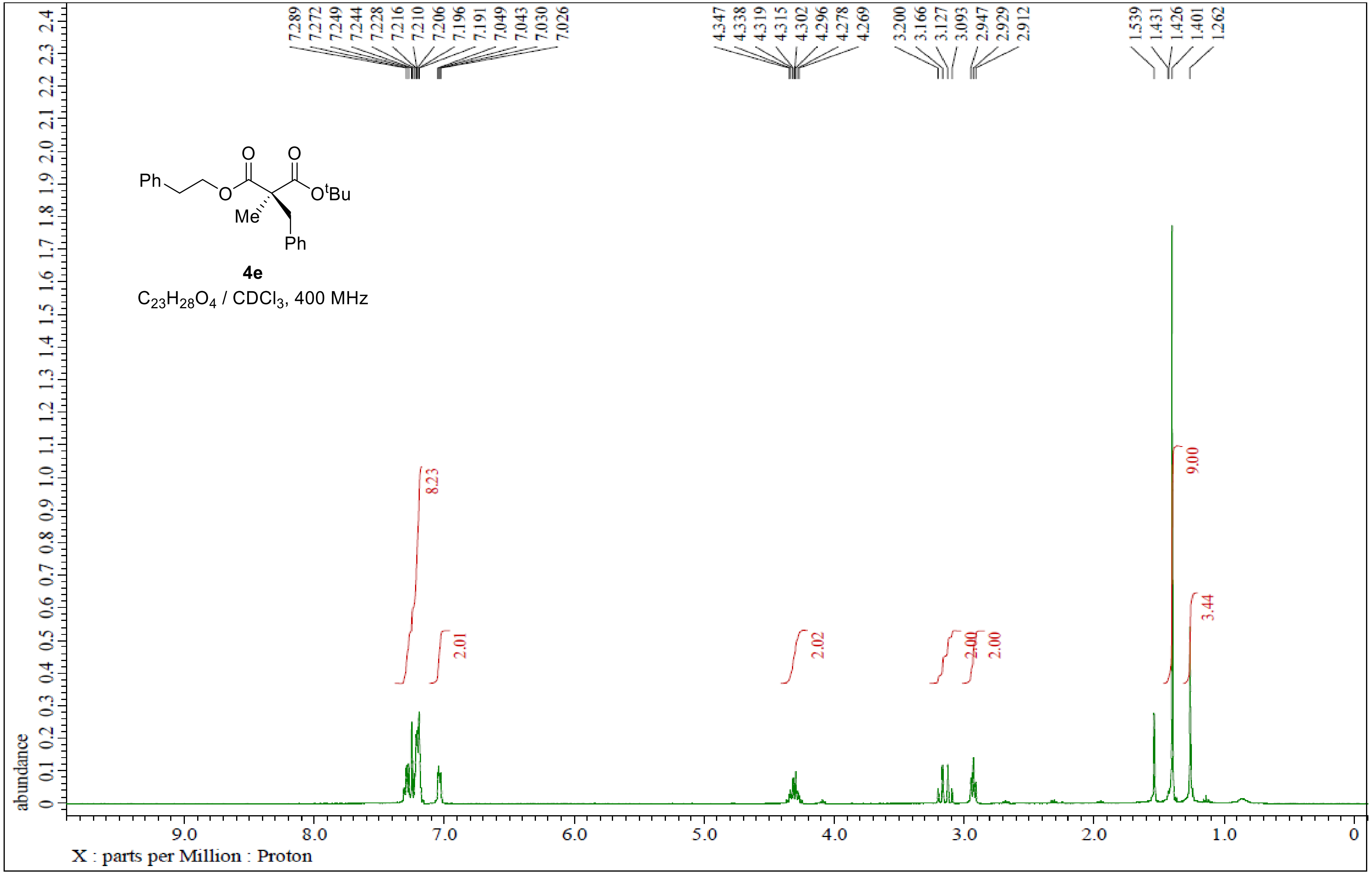

^{13}C-NMR of compound **(4e)**

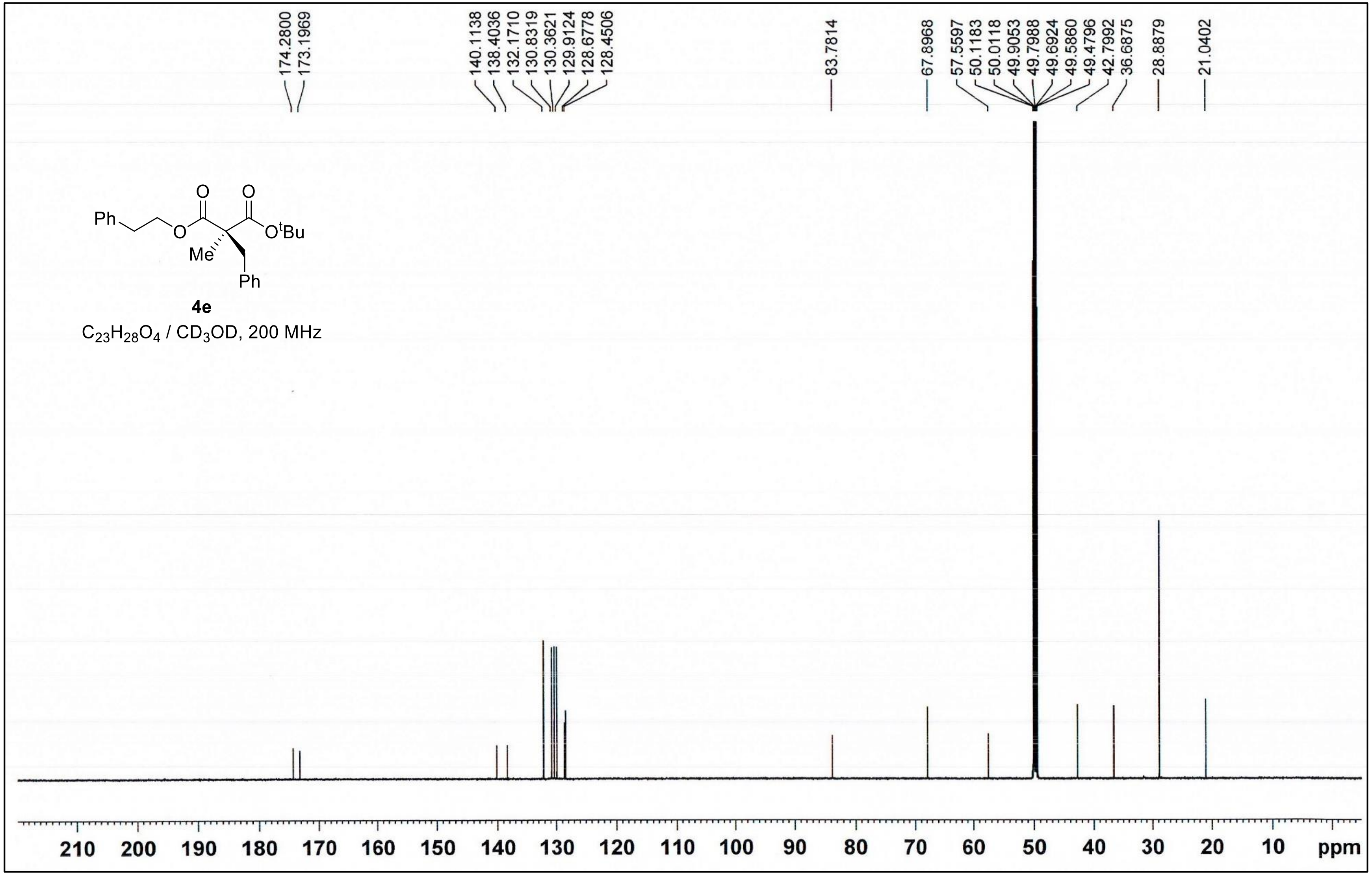

^{1}H-NMR of compound **(5e)**

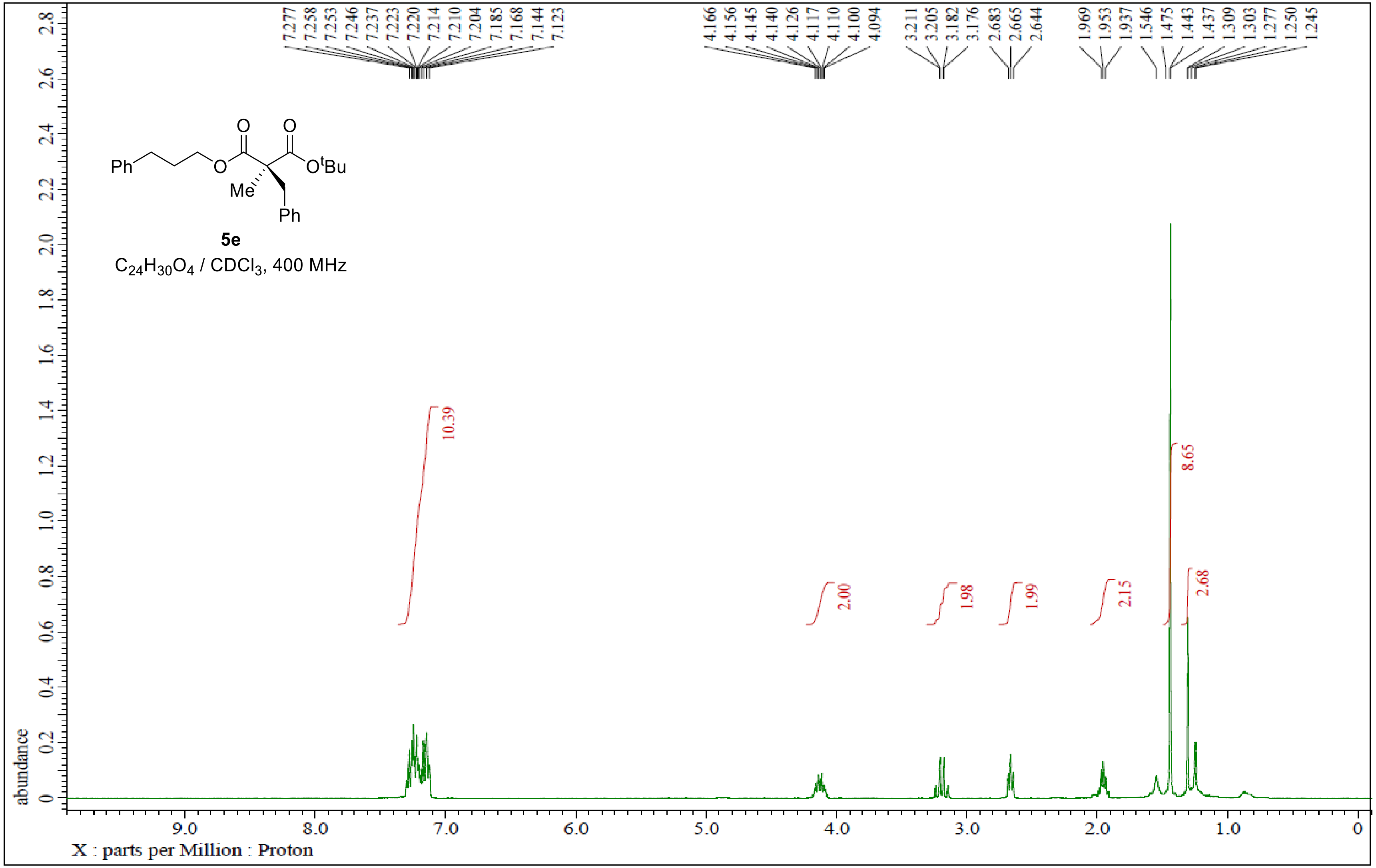

^{13}*C-NMR of compound* **(5e)**

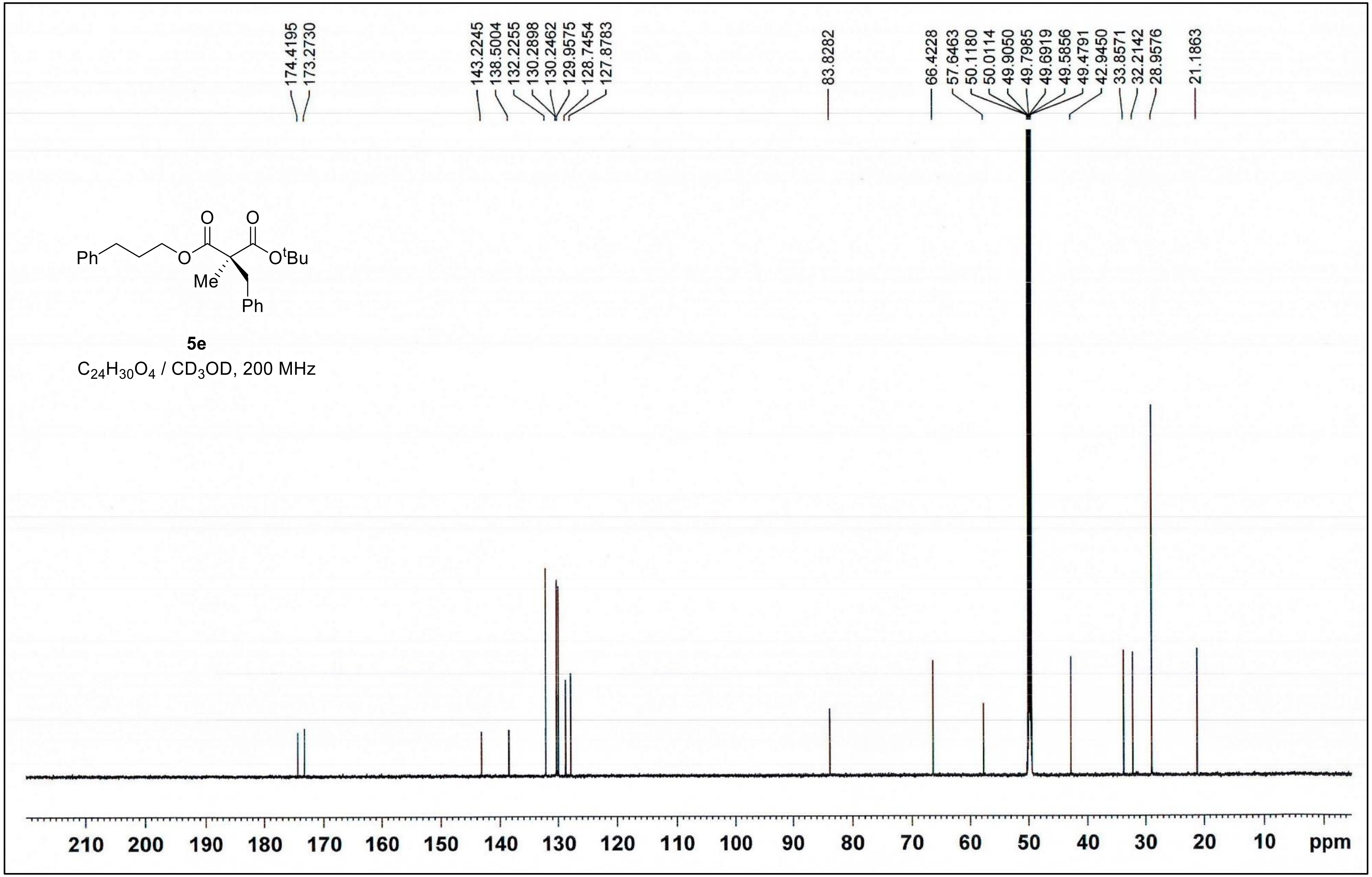

[1]*H-NMR of compound* **(6e)**

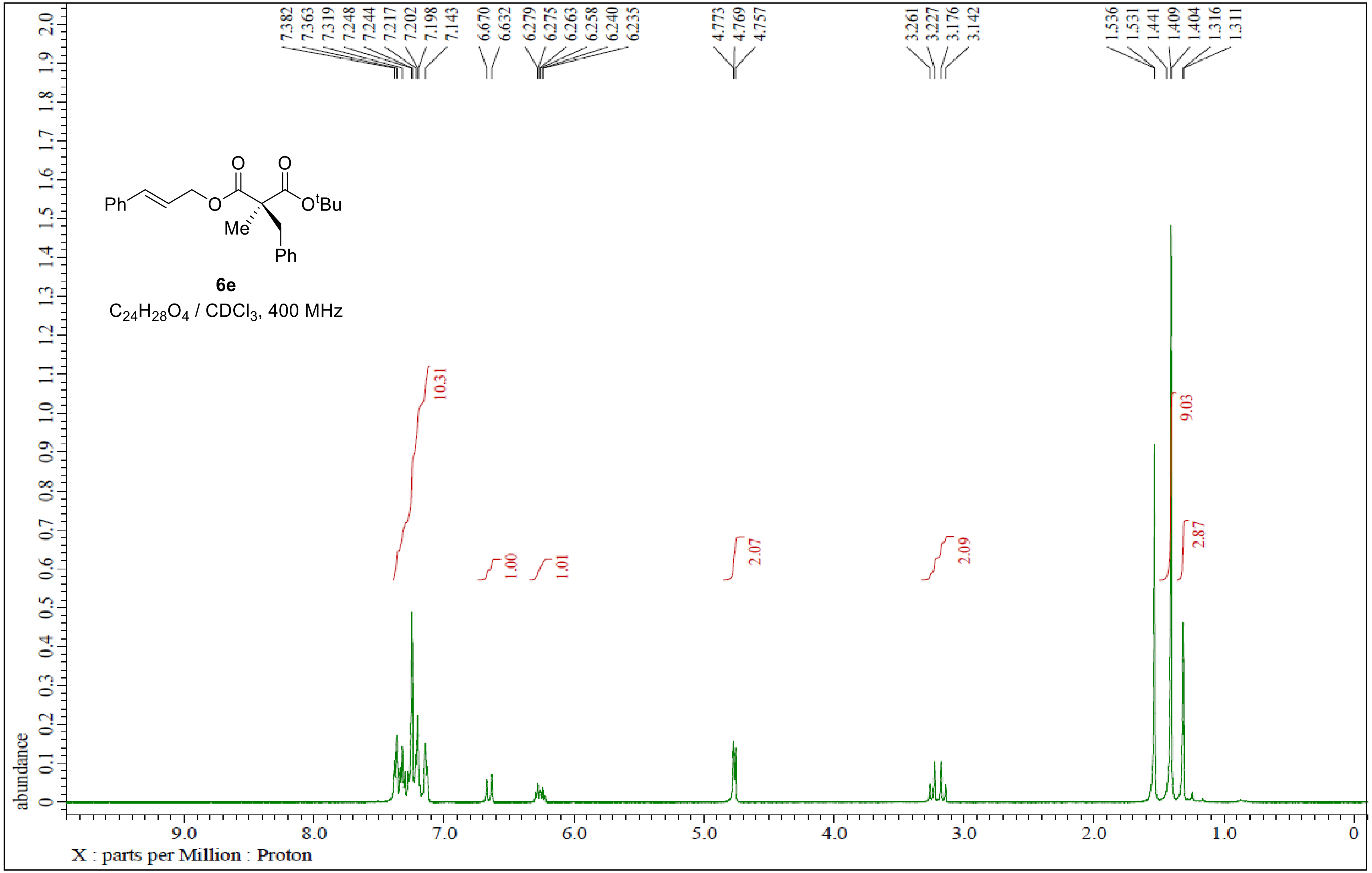

^{13}C-NMR of compound **(6e)**

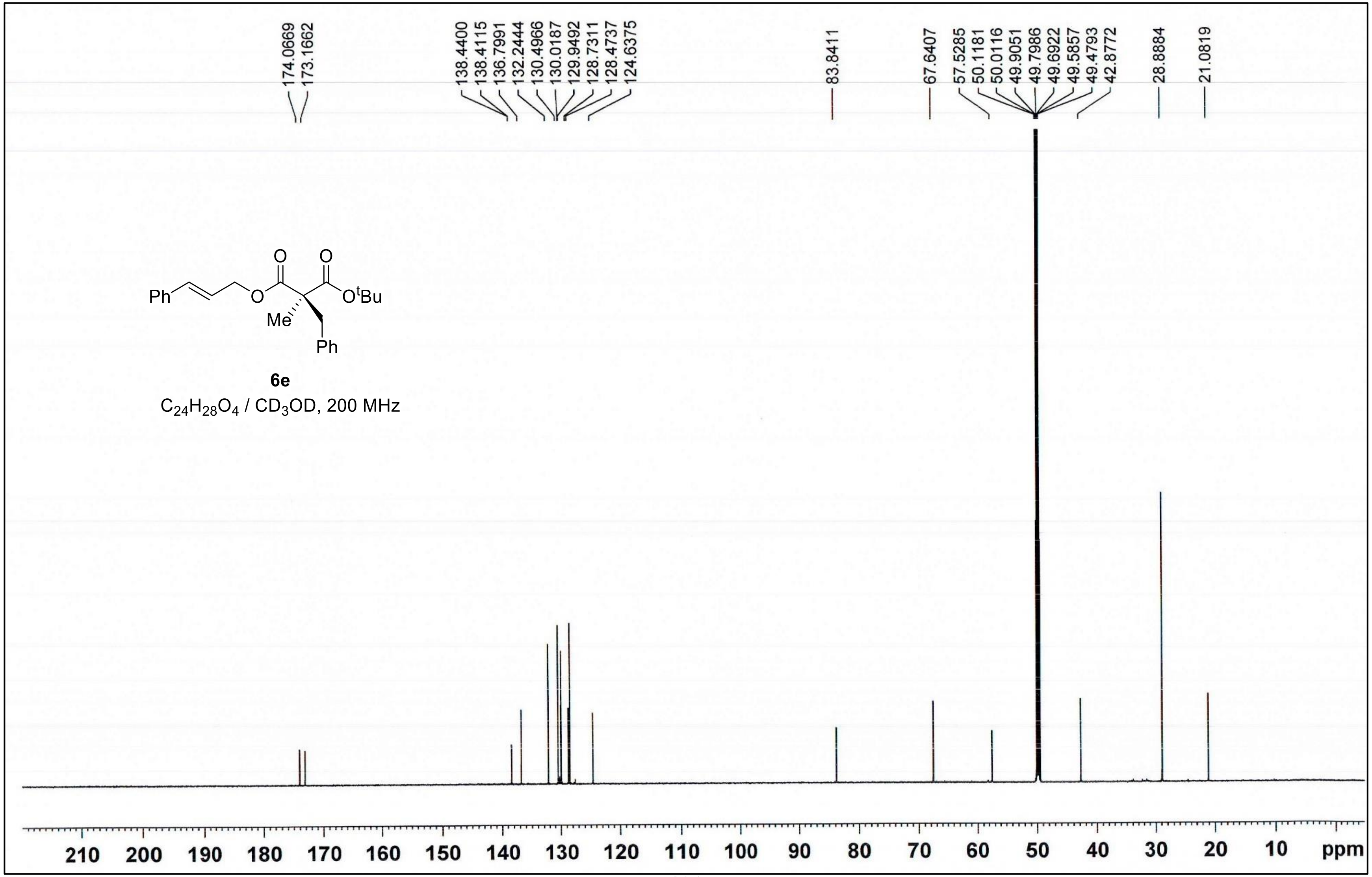

^{1}H-NMR of compound **(7a)**

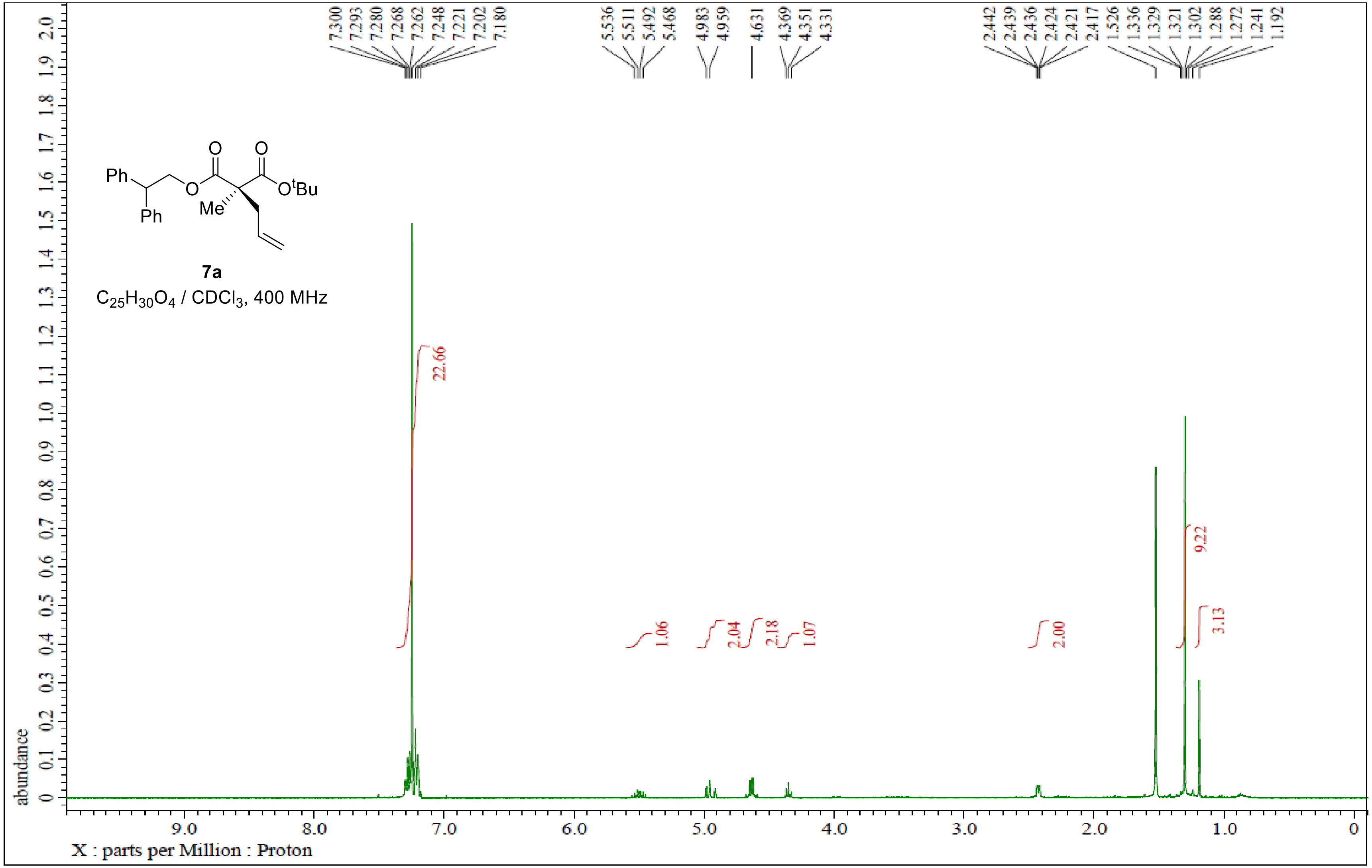

[13]*C-NMR of compound* (**7a**)

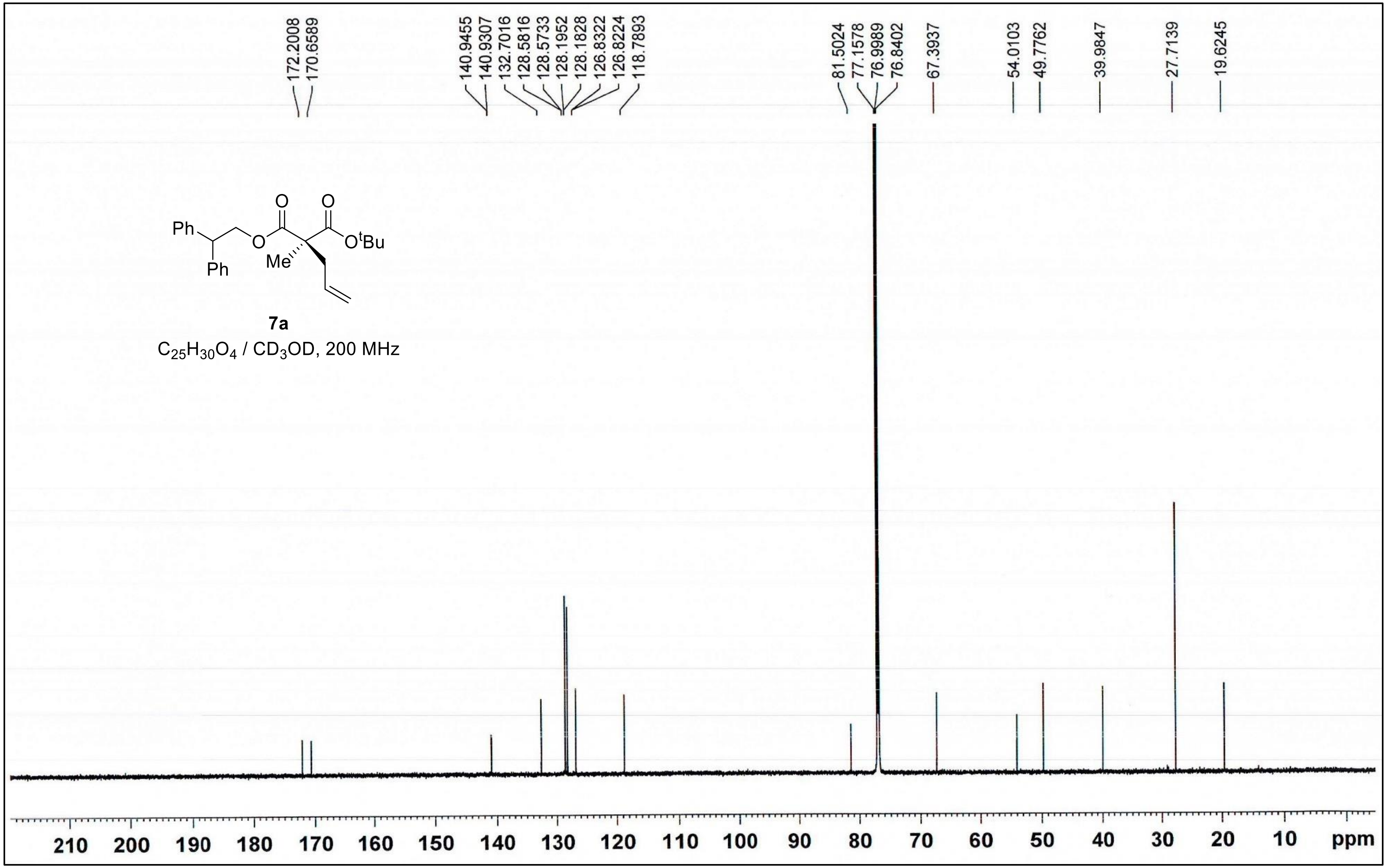

[1]*H-NMR of compound* **(7b)**

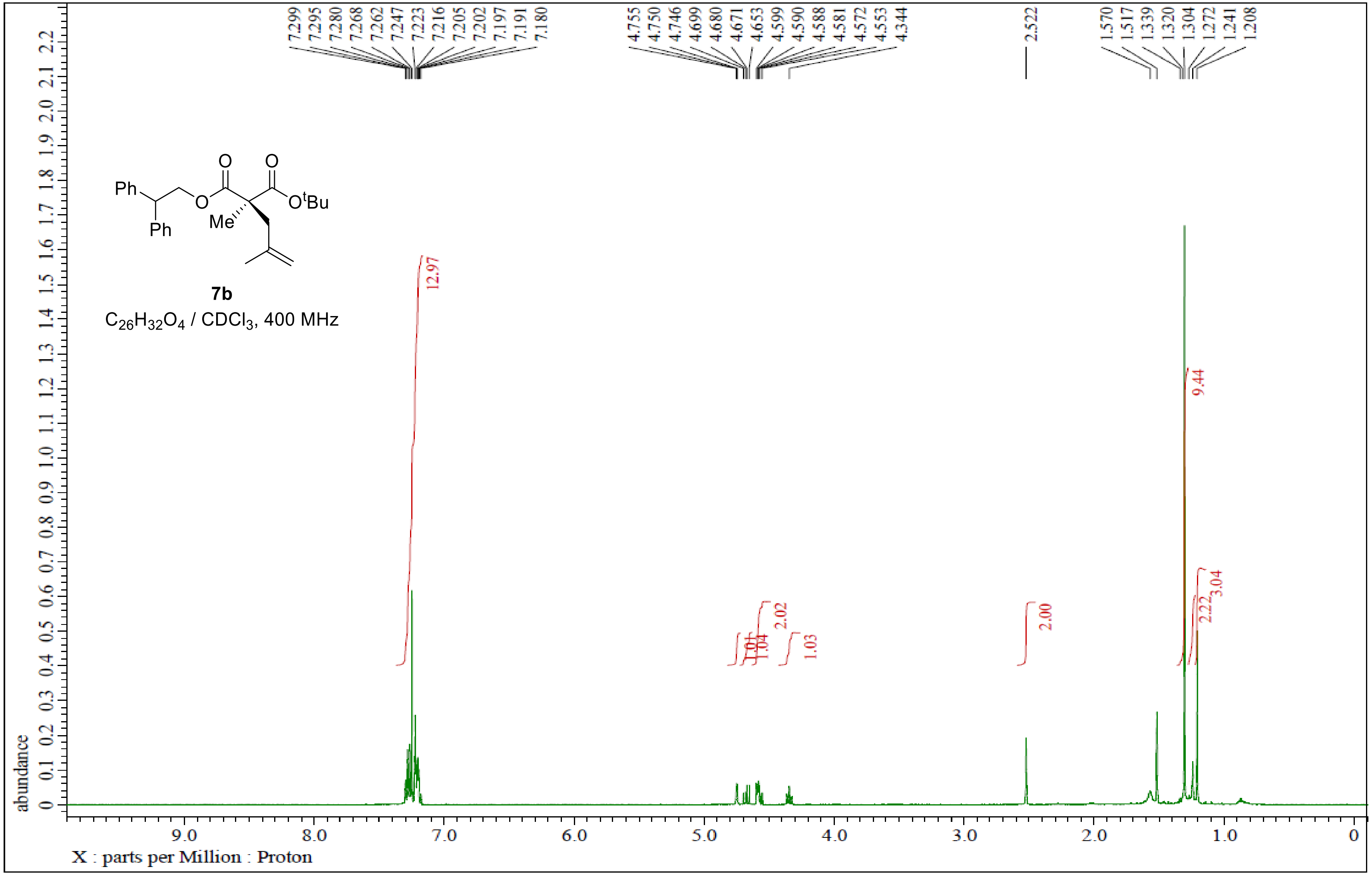

^{13}*C-NMR of compound* **(7b)**

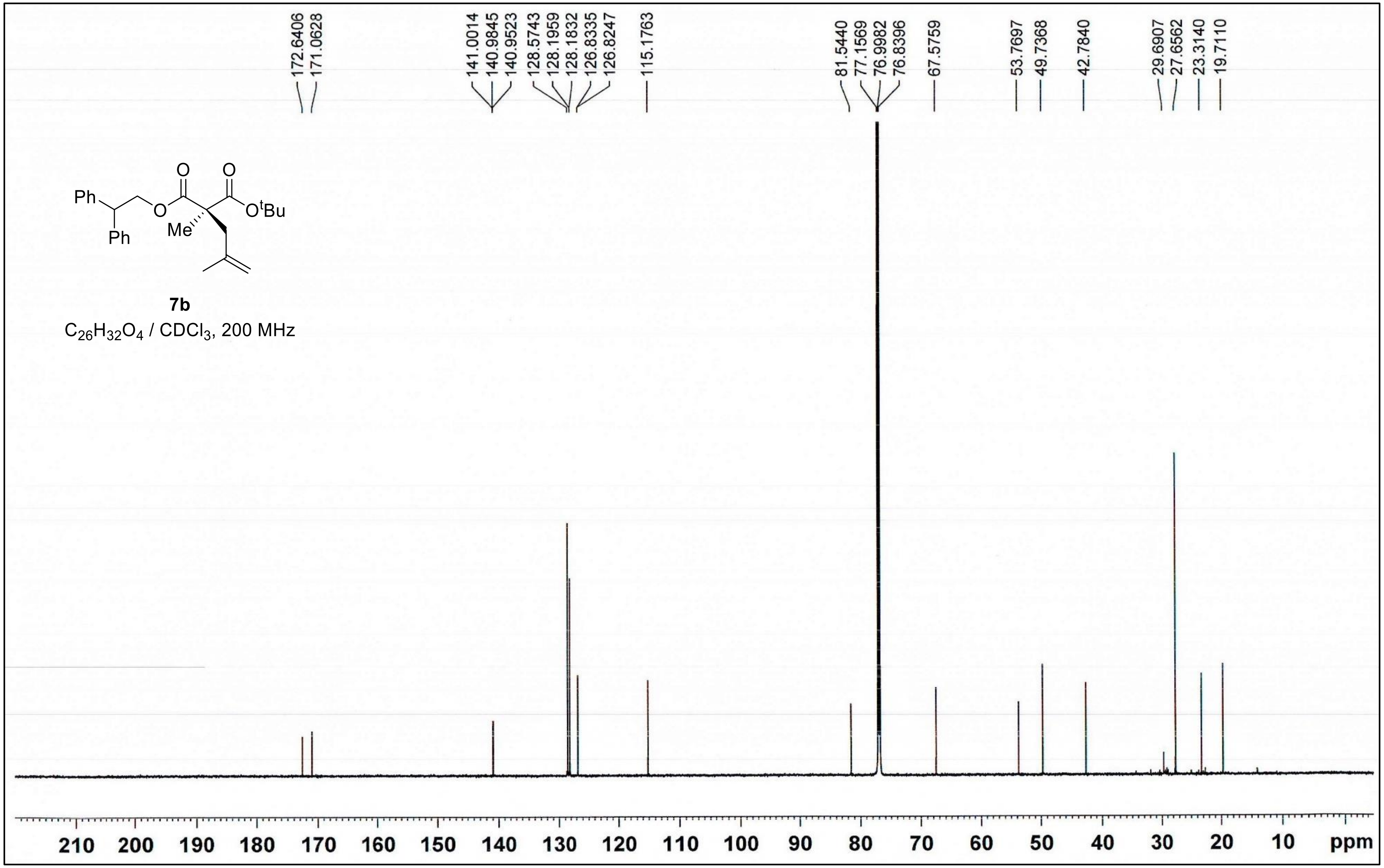

^{1}H-NMR of compound **(7c)**

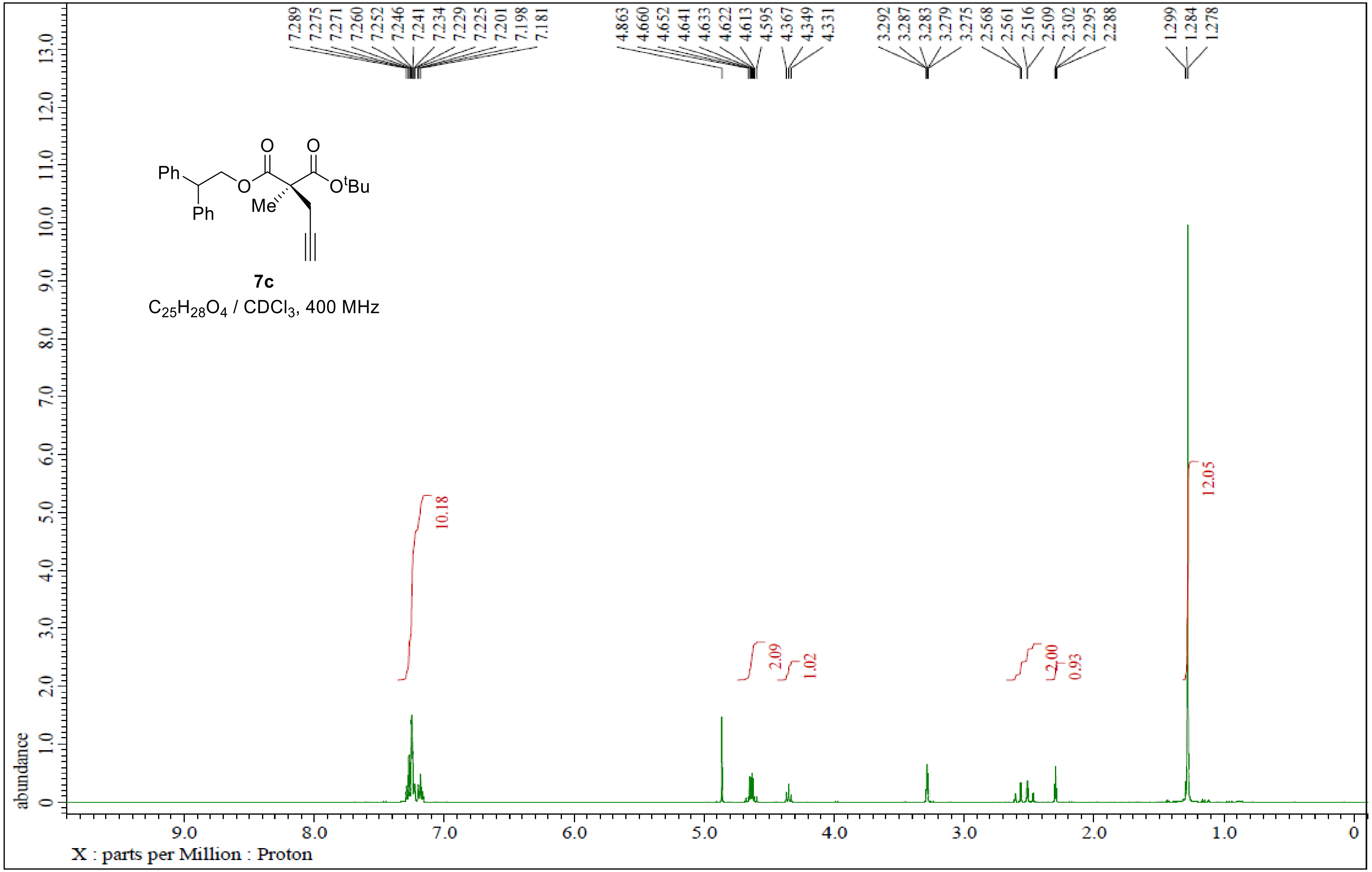

^{13}C-NMR of compound **(7c)**

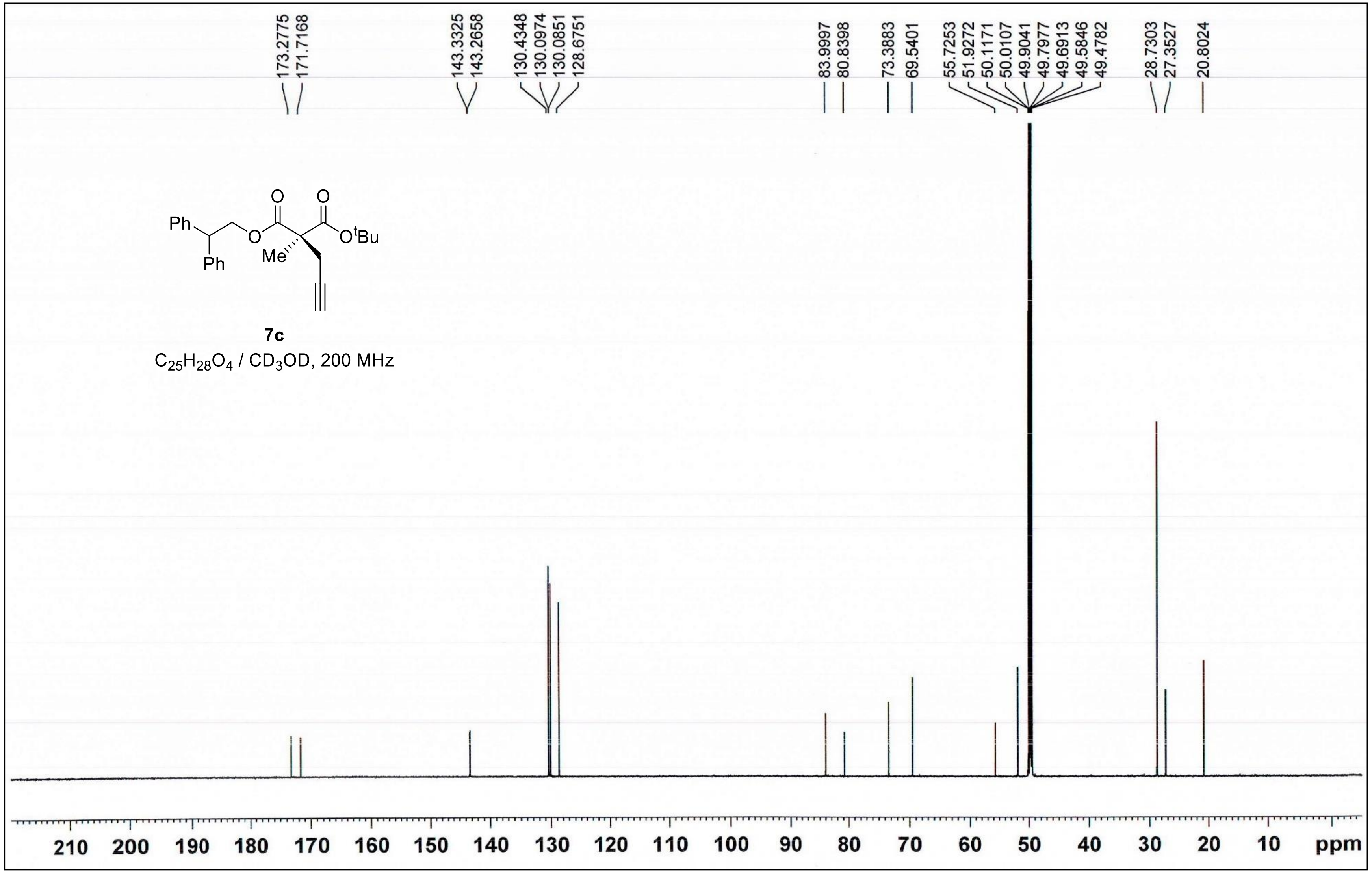

^{1}H-NMR of compound **(7d)**

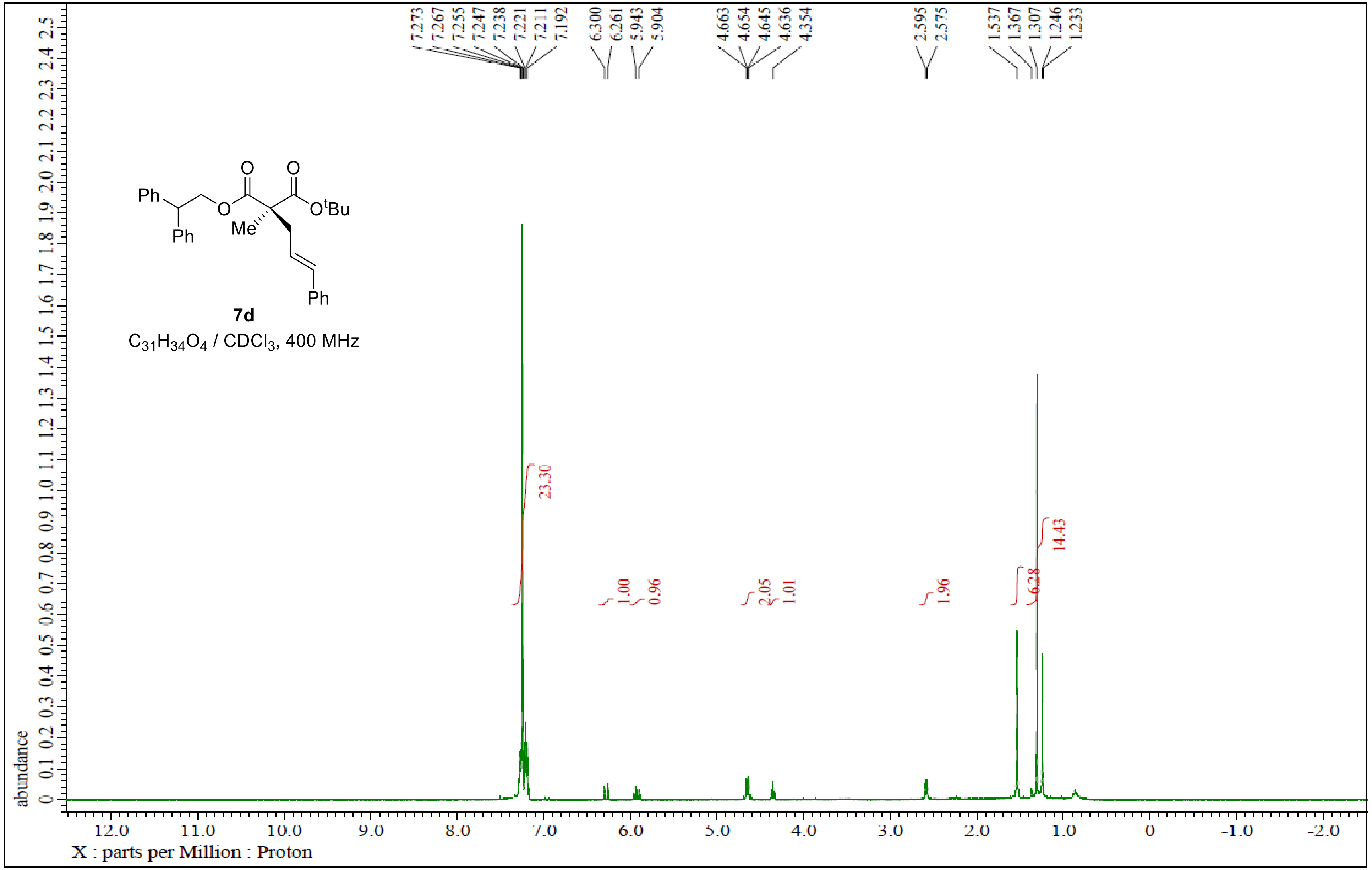

^{13}C-NMR of compound **(7d)**

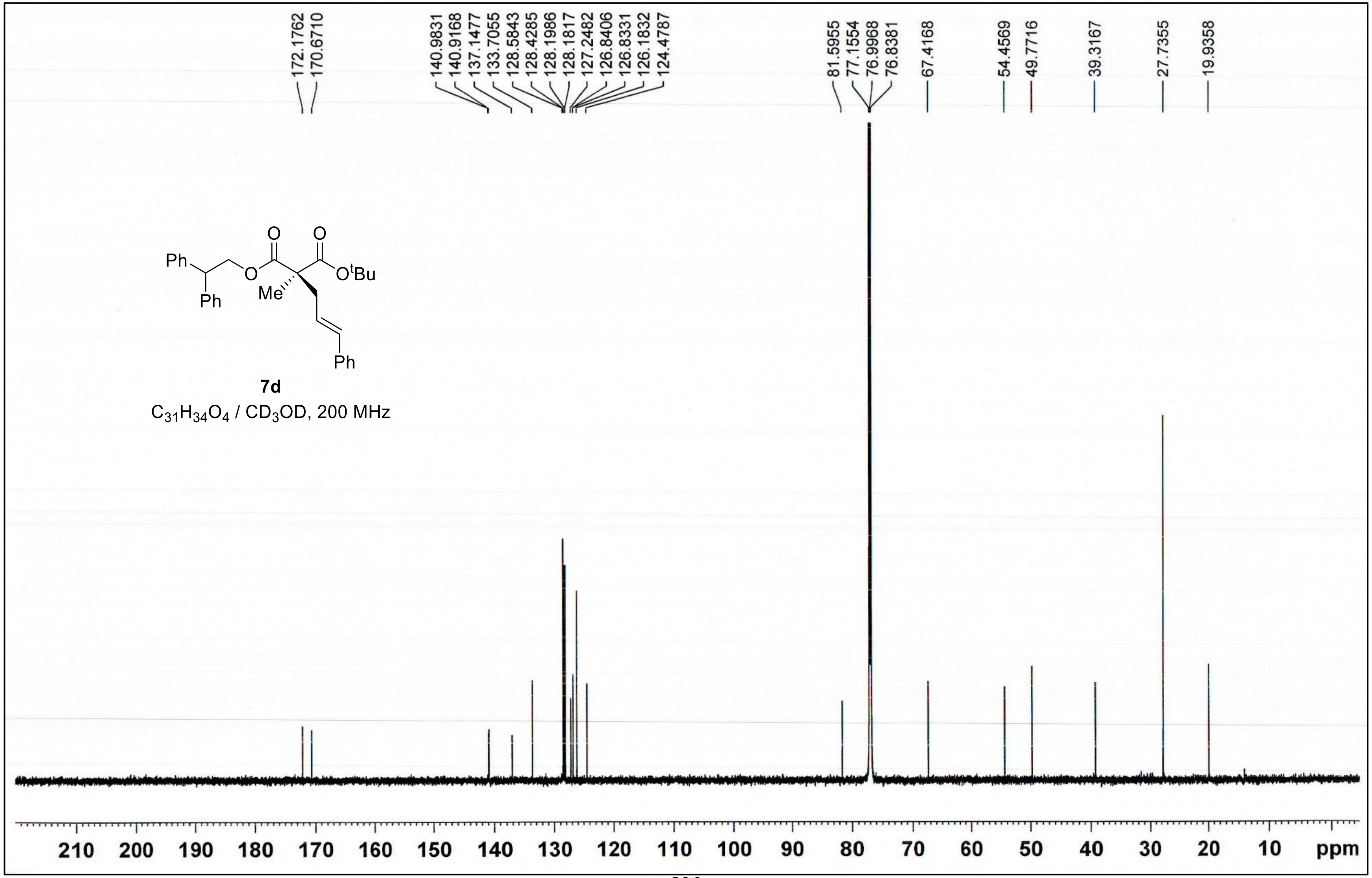

[1]*H-NMR of compound* **(7e)**

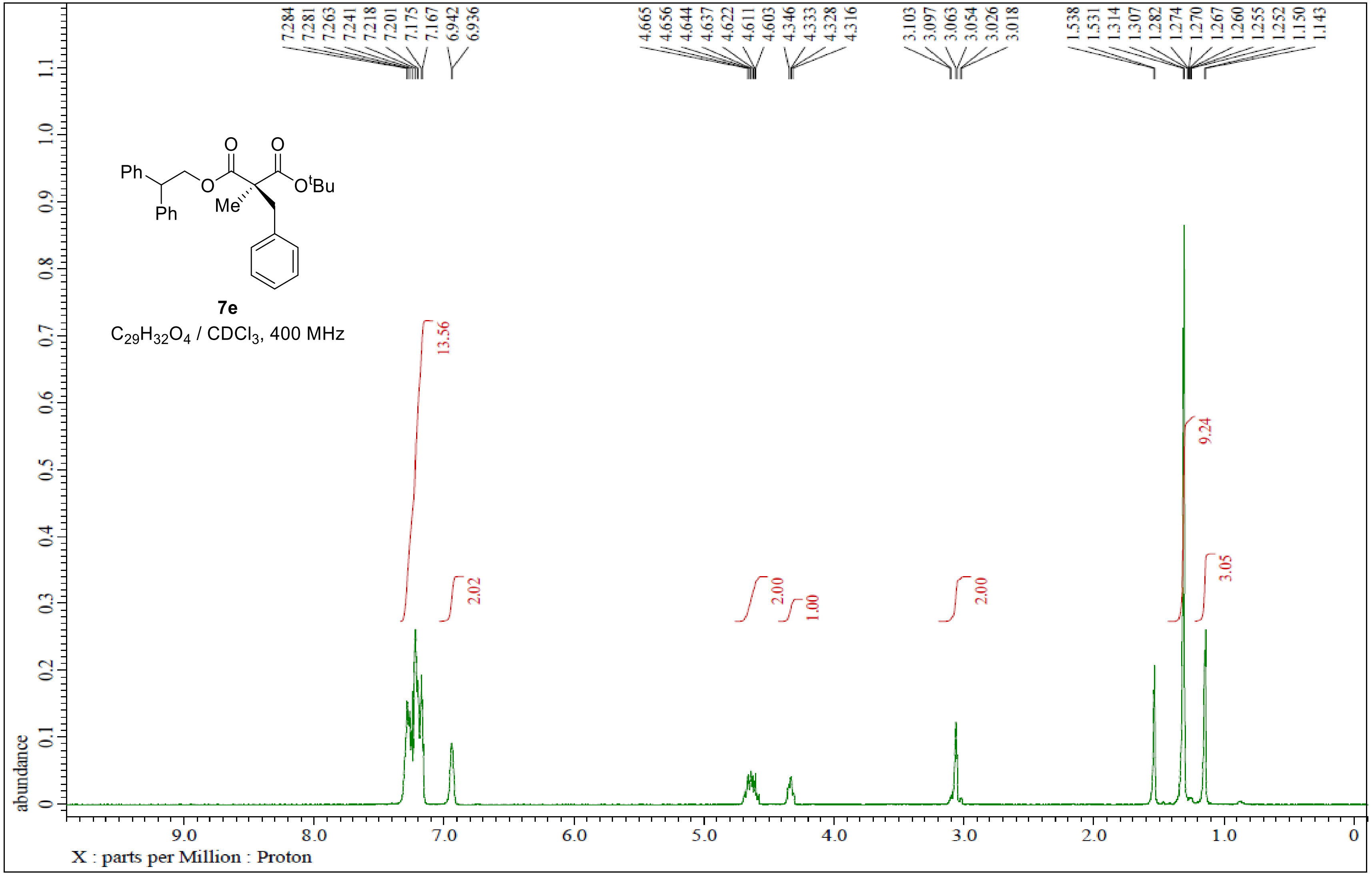

^{13}C-NMR of compound **(7e)**

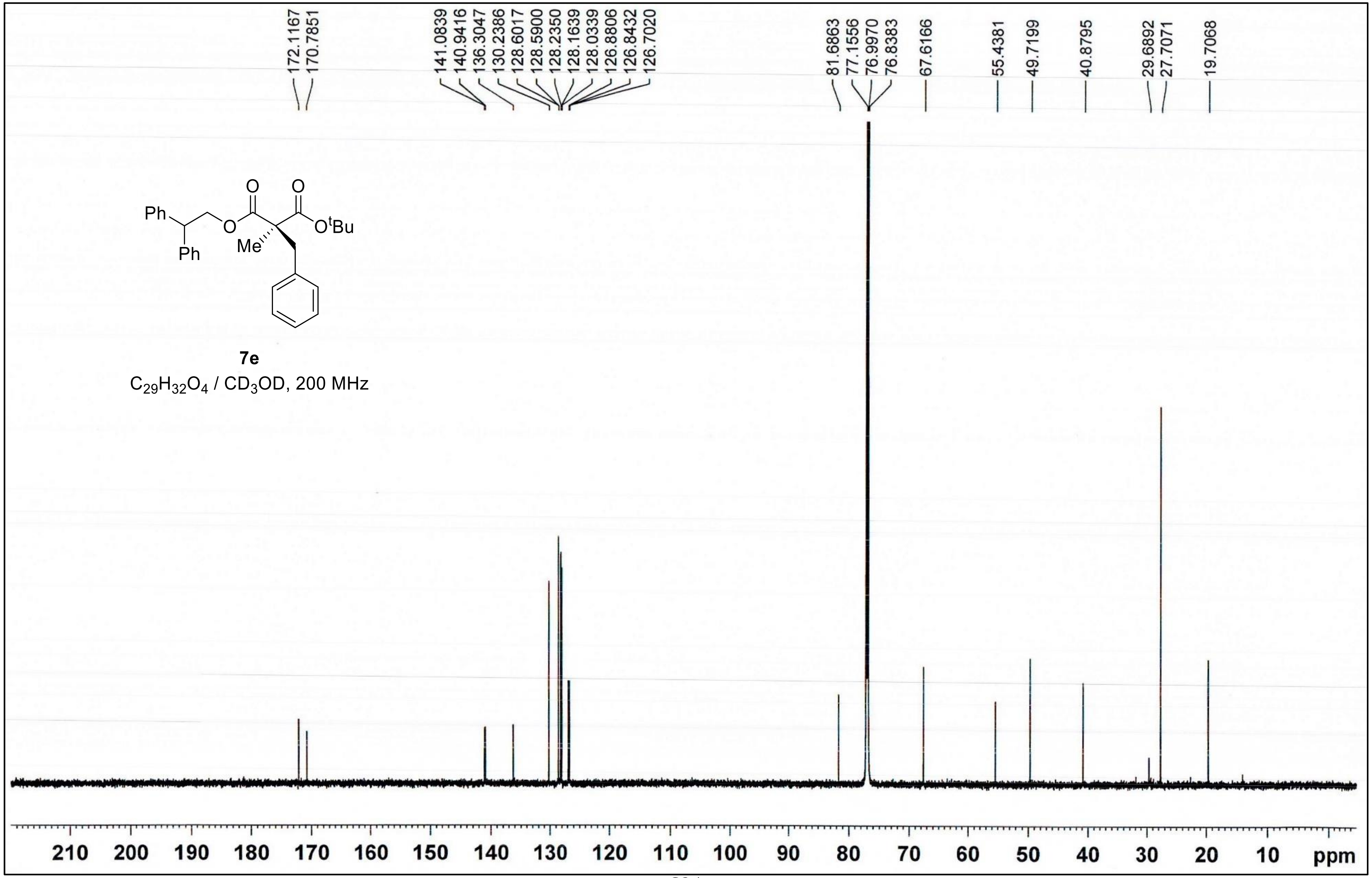

^{1}H-NMR of compound **(7f)**

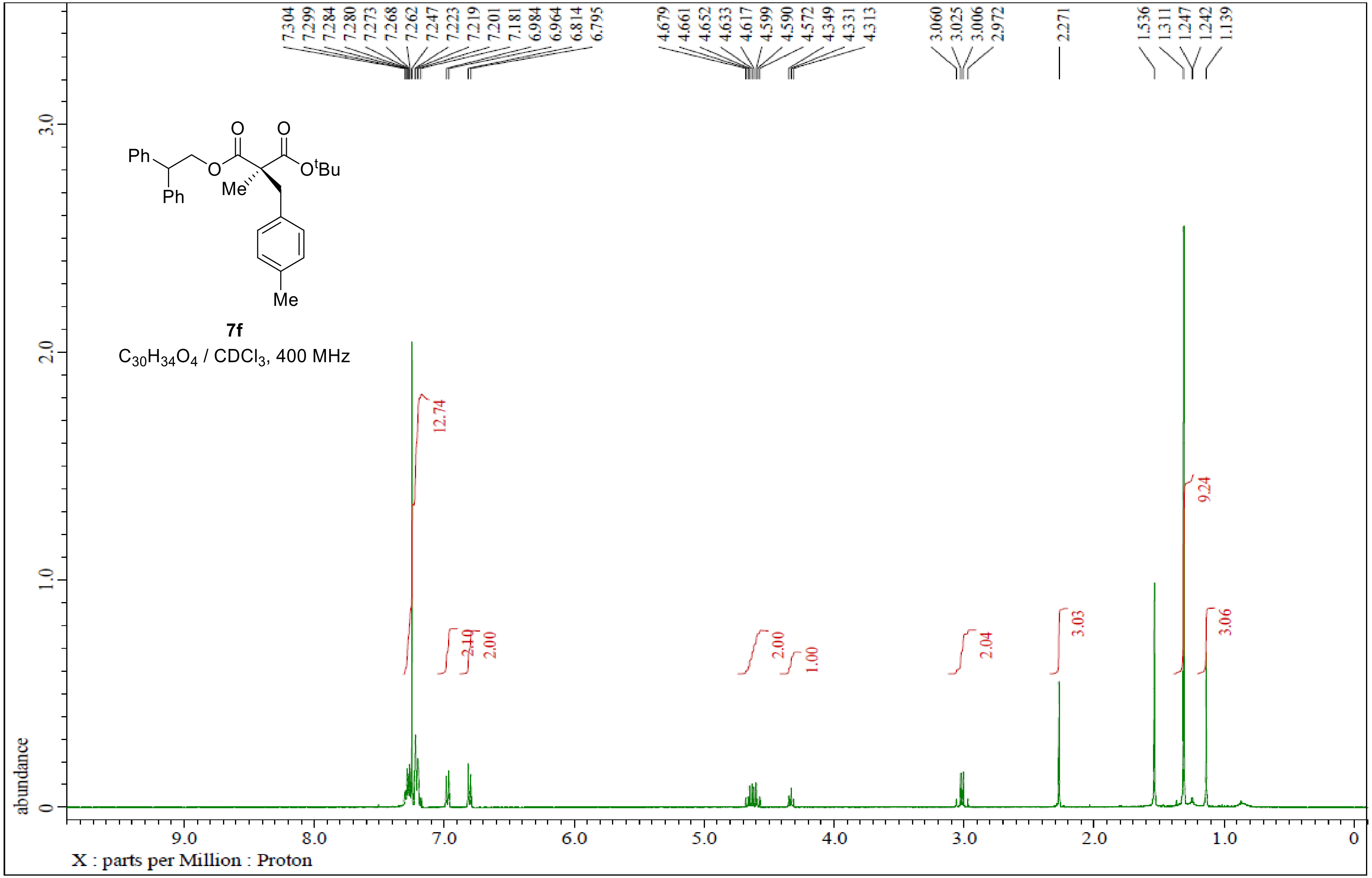

^{13}*C-NMR of compound* **(7f)**

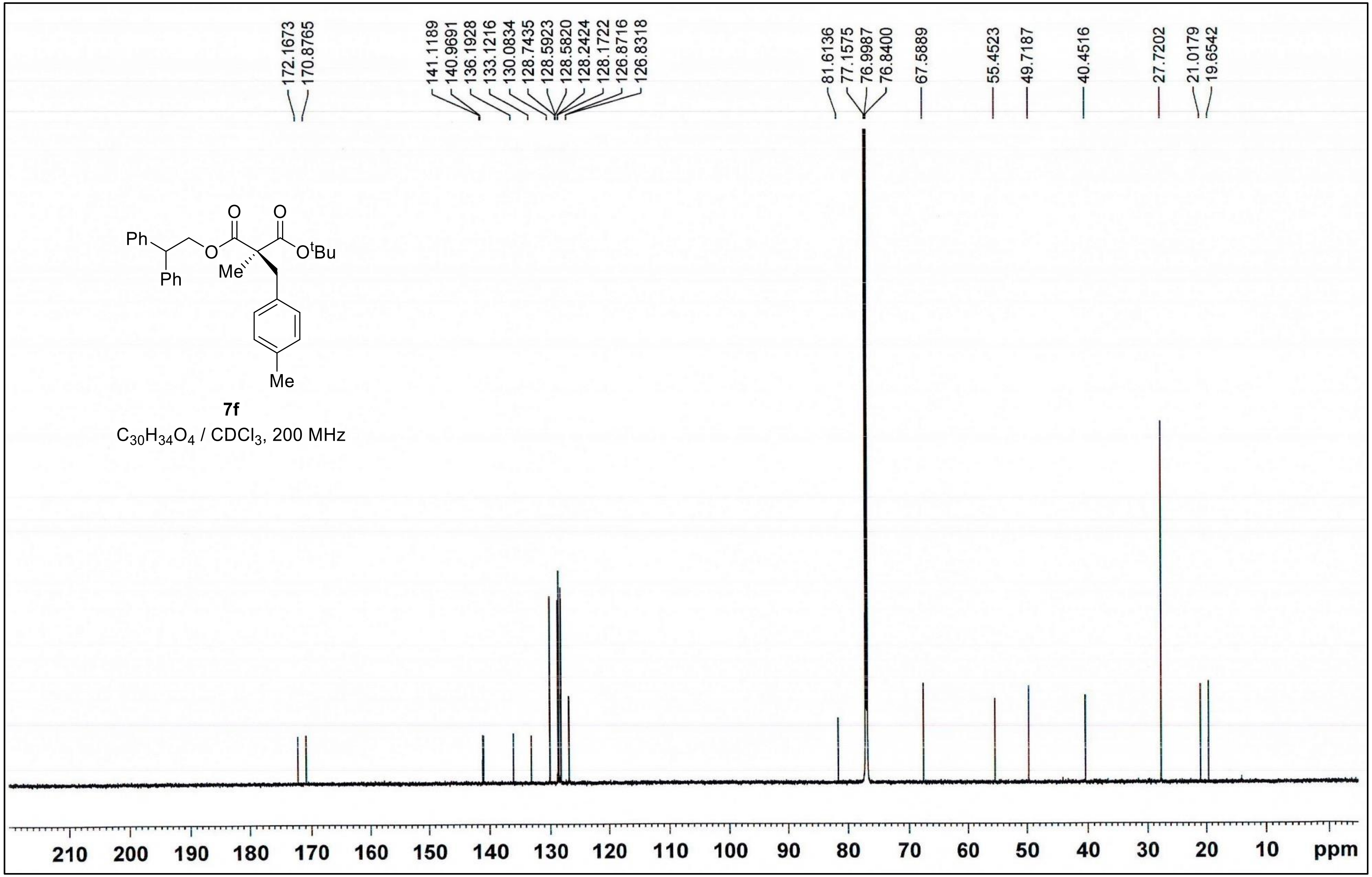

^{1}H-NMR of compound **(7g)**

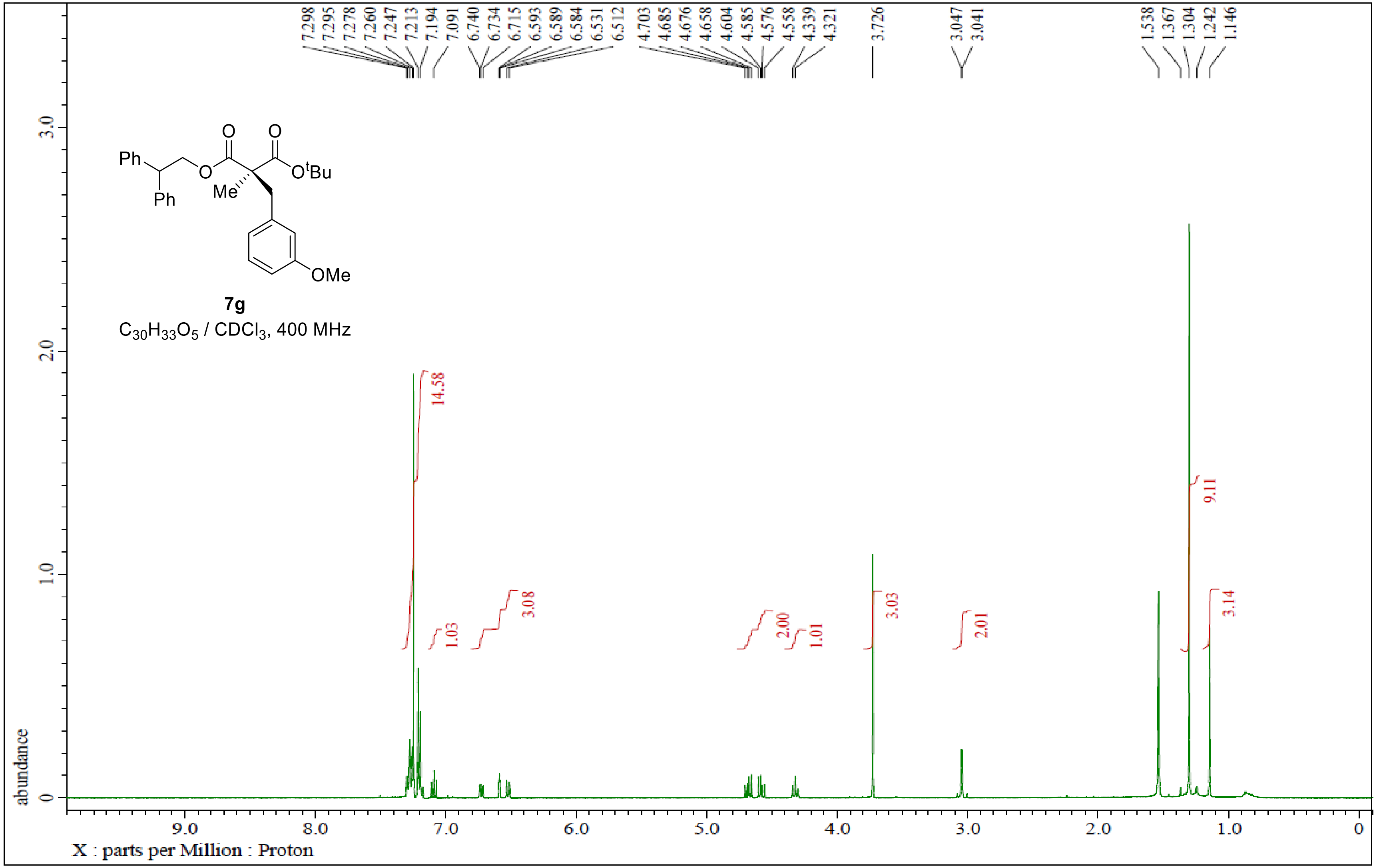

^{13}*C-NMR of compound* **(7g)**

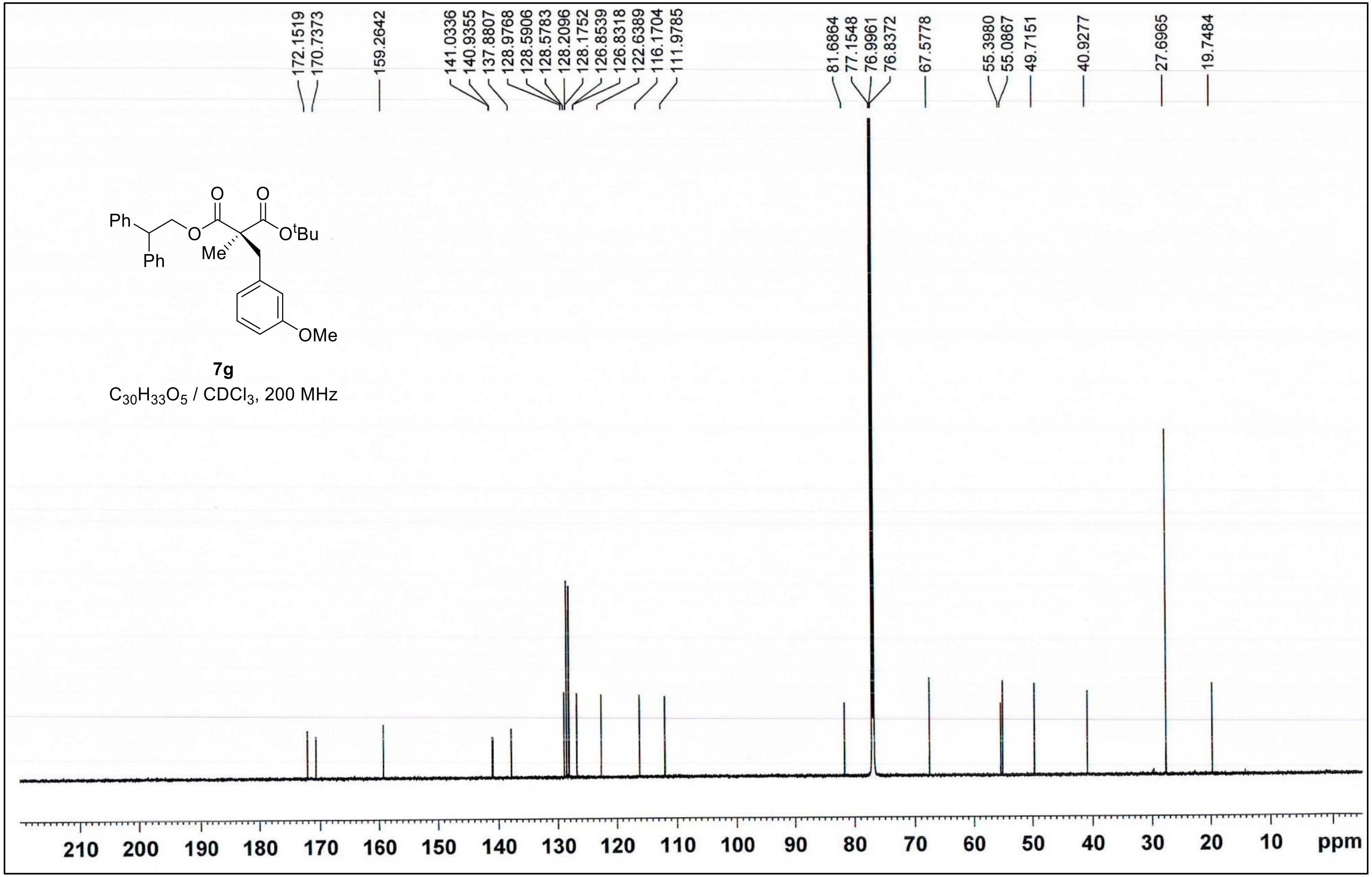

^{1}H-NMR of compound **(7h)**

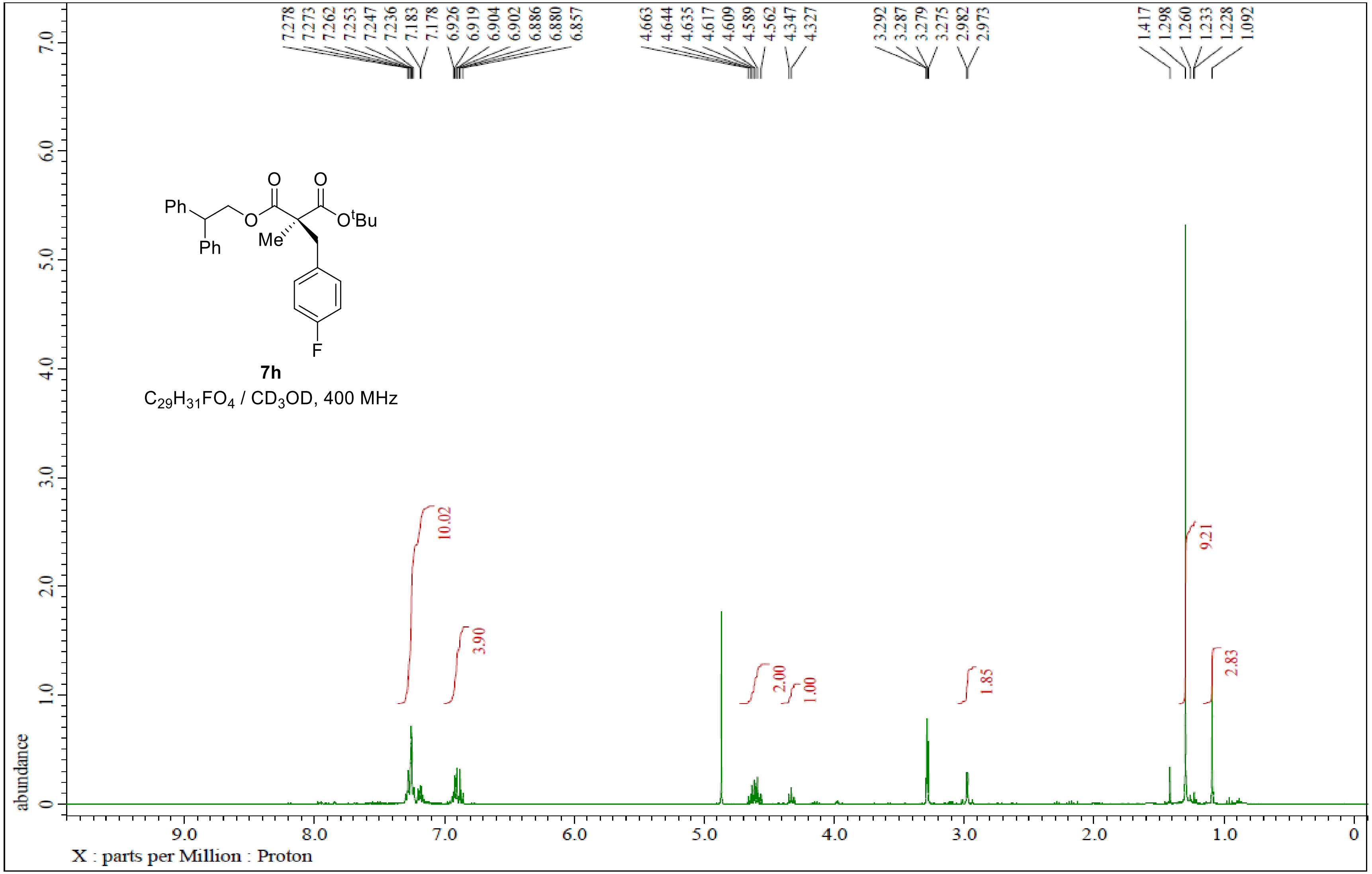

^{13}C-NMR of compound **(7h)**

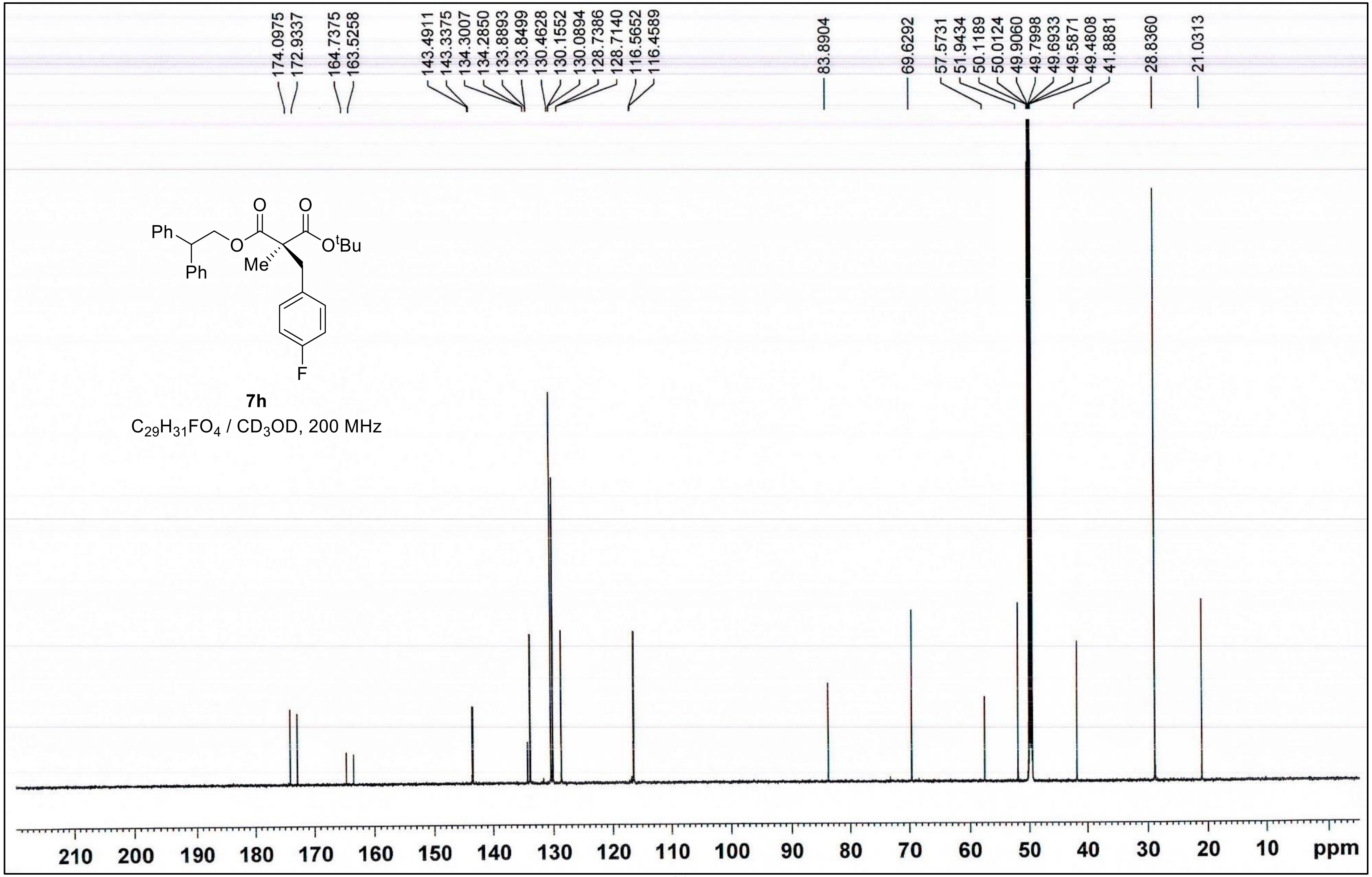

^{1}H-NMR of compound **(7i)**

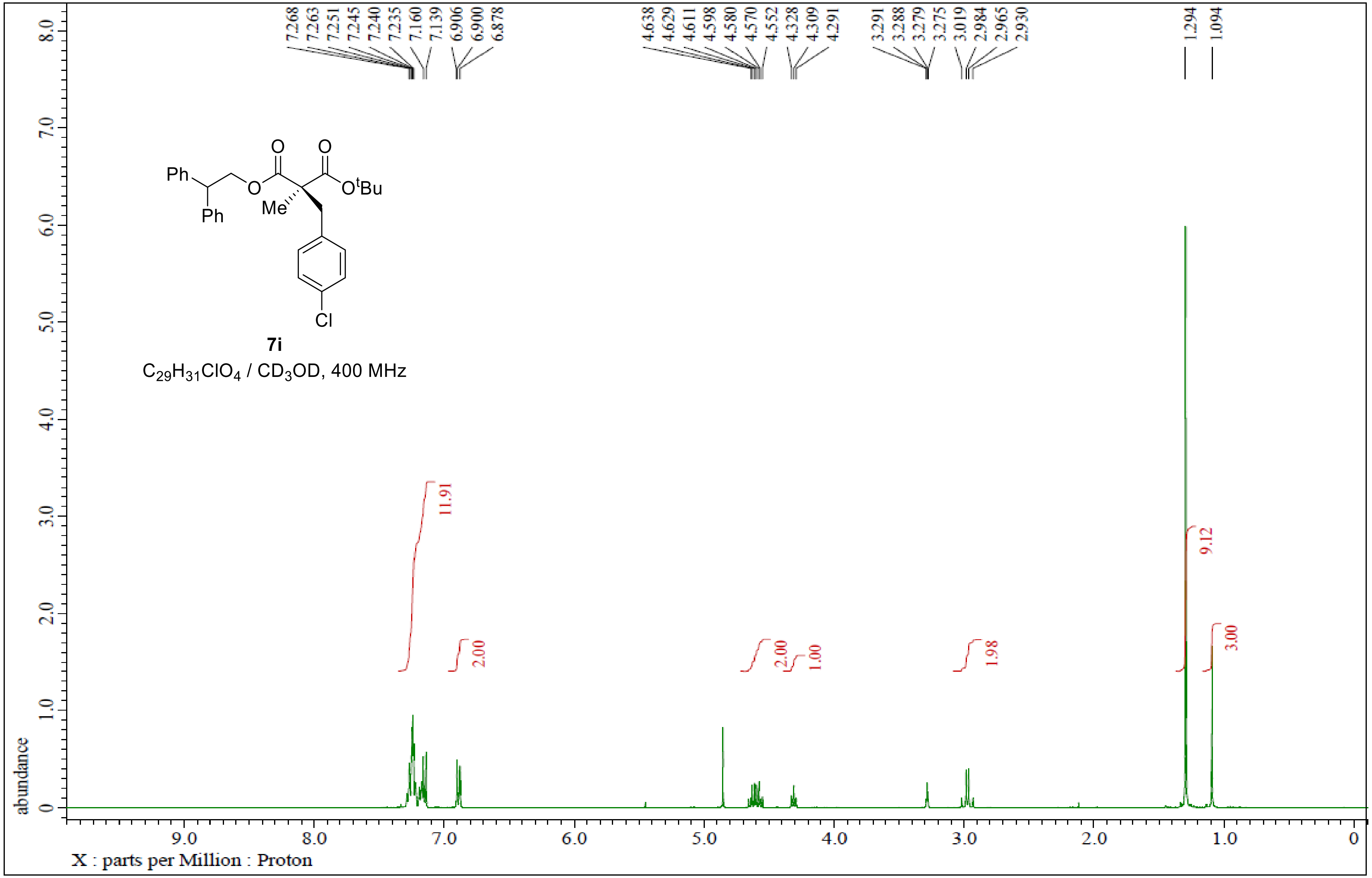

^{13}C-NMR of compound **(7i)**

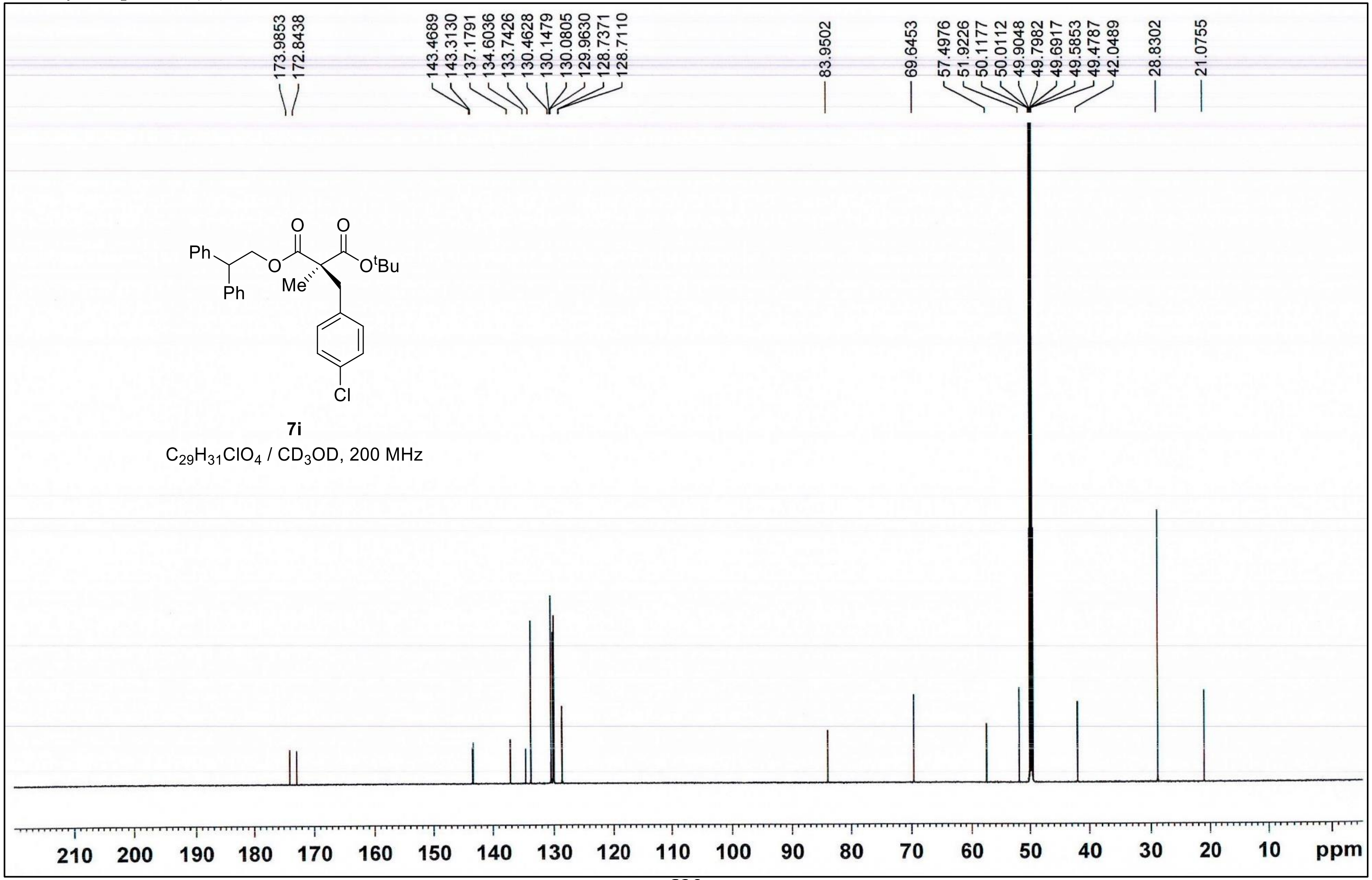

^{1}H-NMR of compound **(7j)**

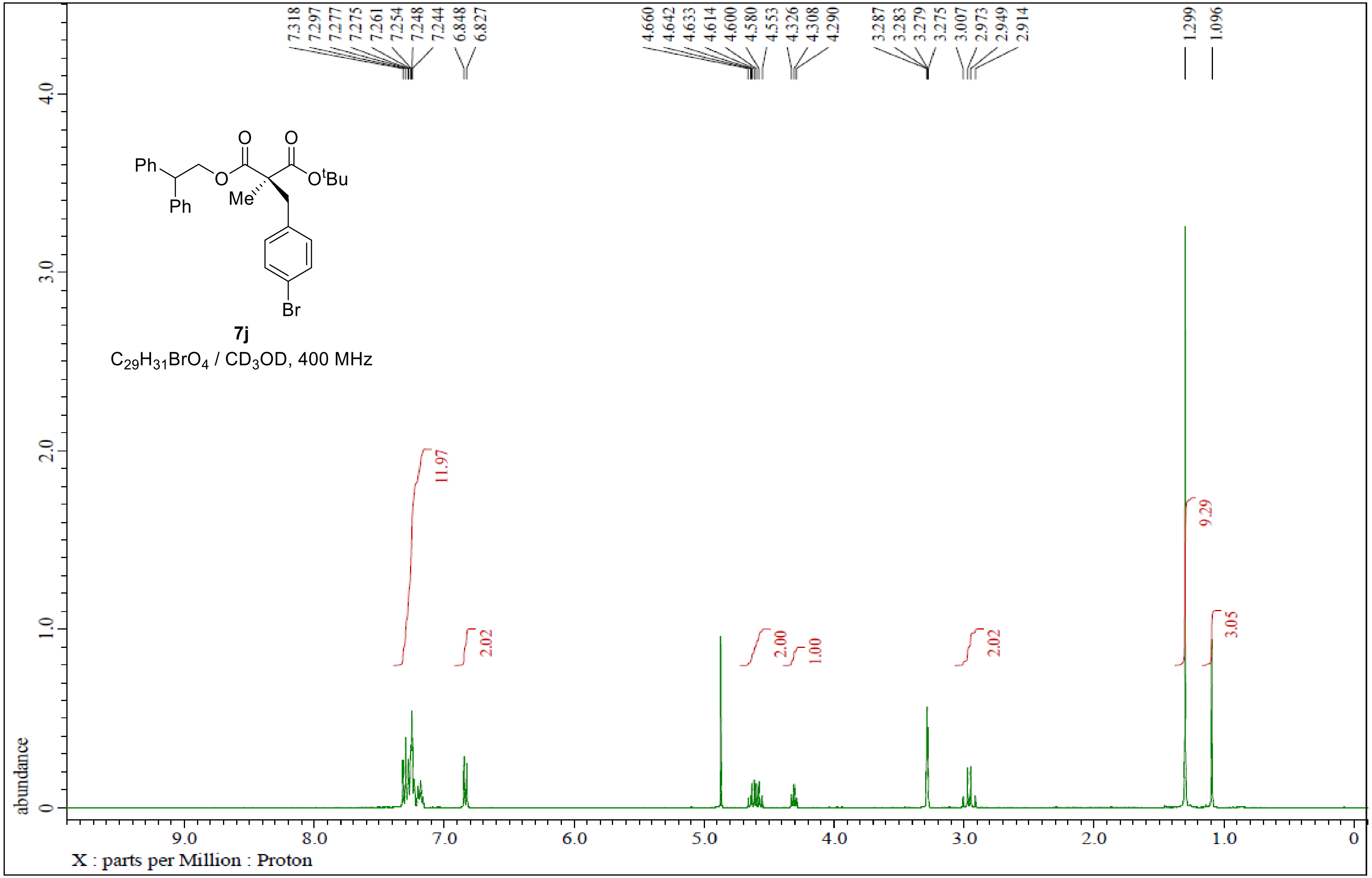

^{13}*C-NMR of compound* **(7j)**

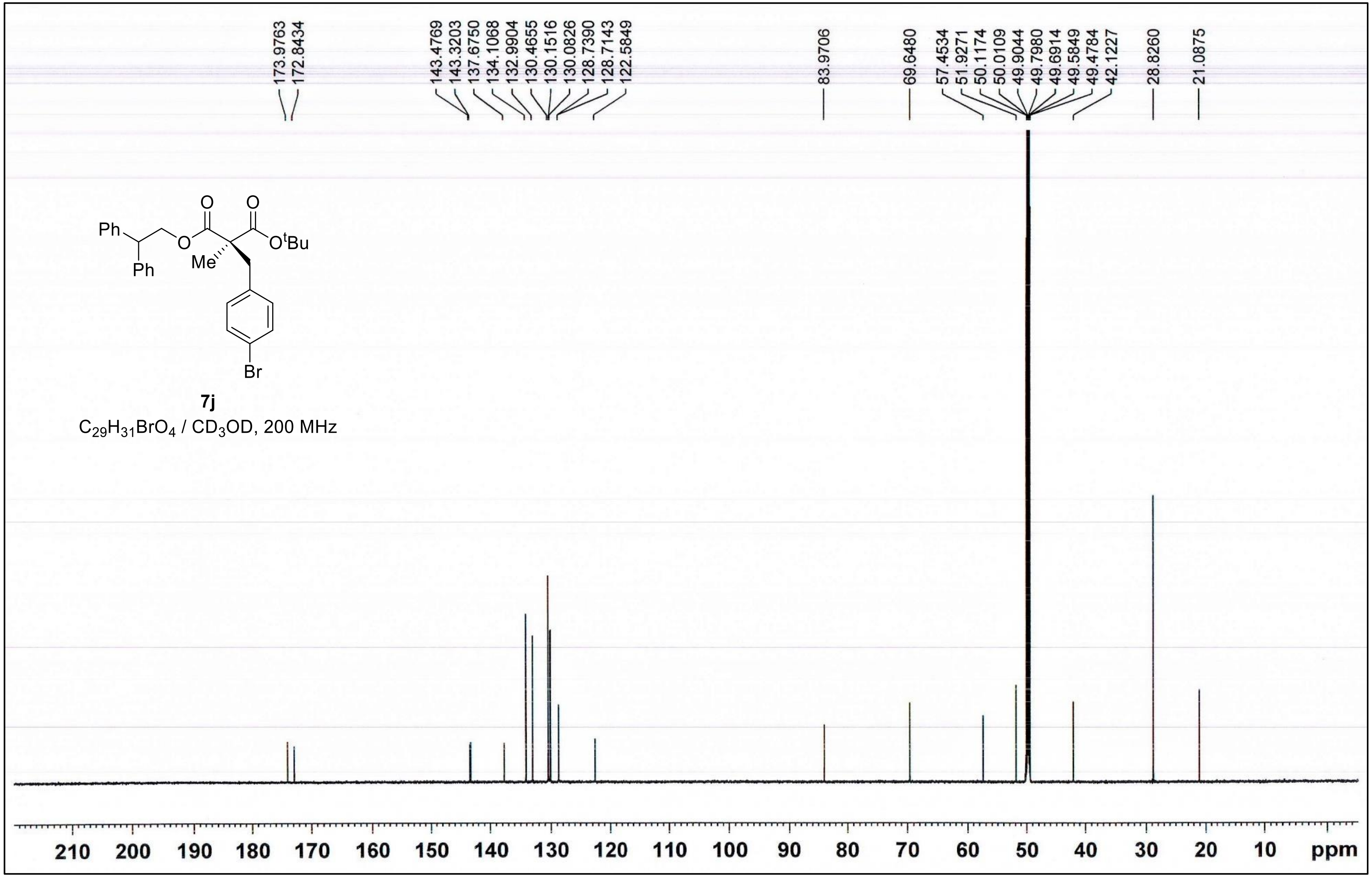

^{1}H-NMR of compound **(7k)**

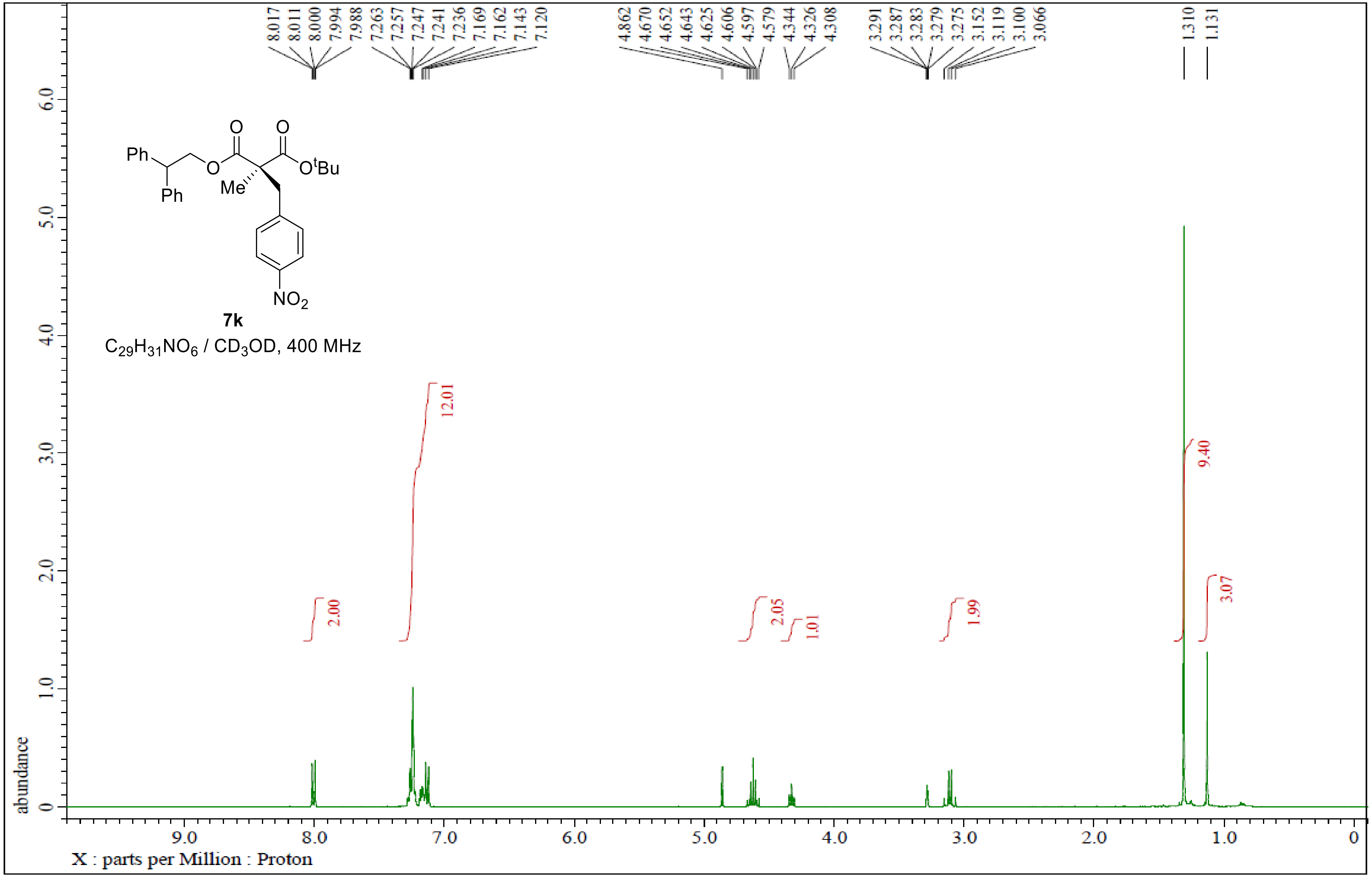

^{13}C-NMR of compound **(7k)**

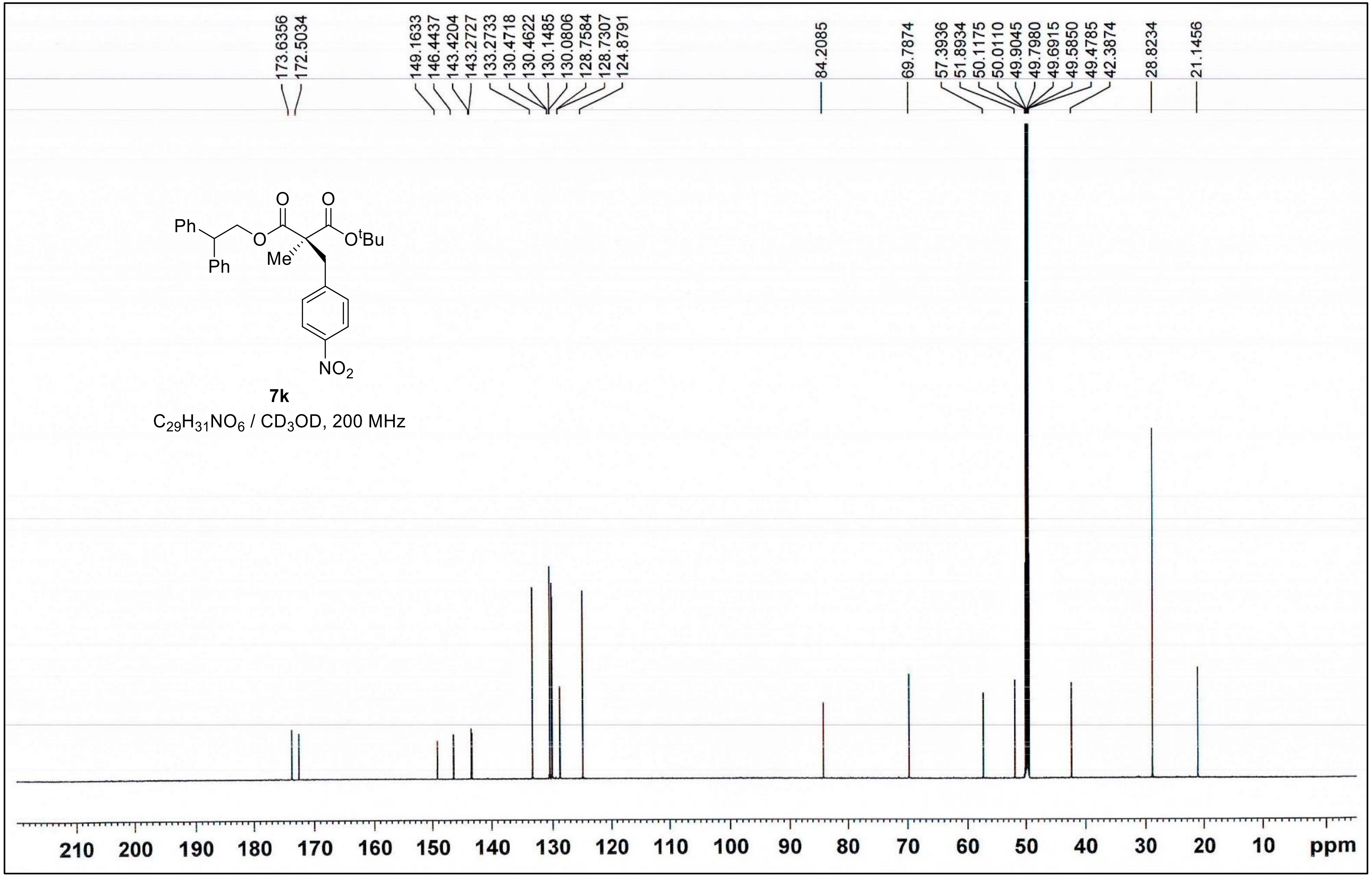

^{1}H-NMR of compound **(7l)**

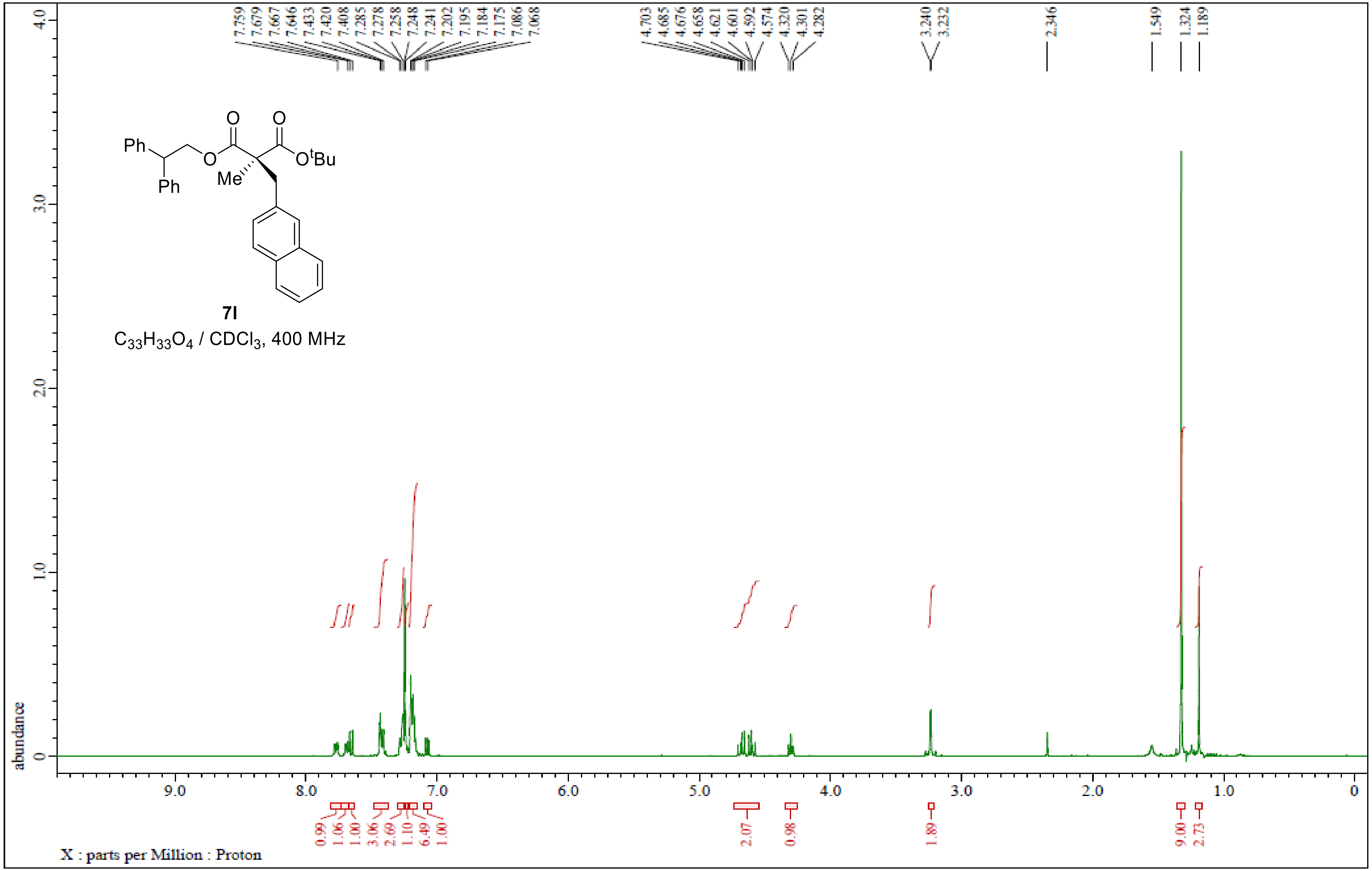

^{13}*C-NMR of compound* **(7l)**

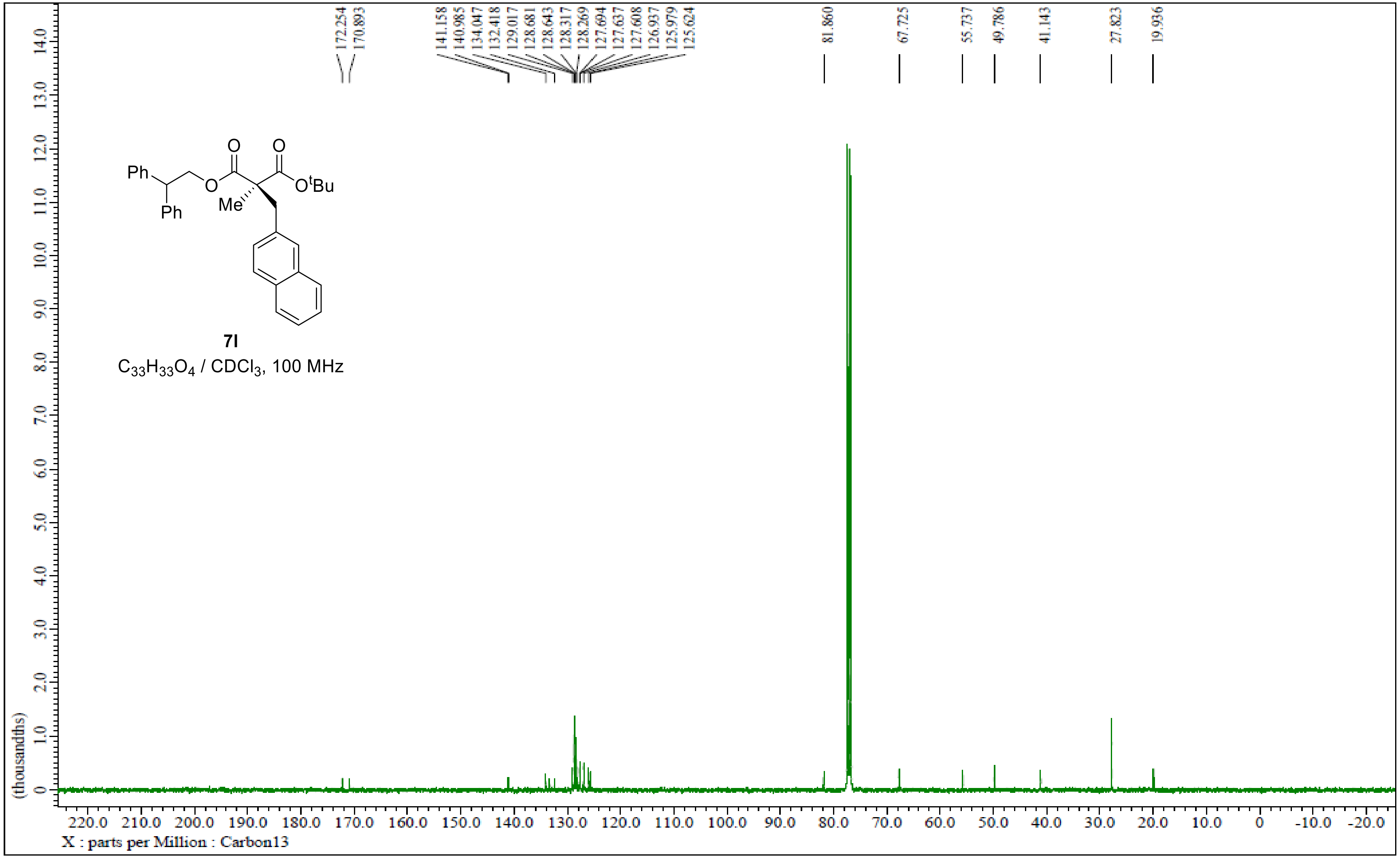

[1]*H-NMR of compound* **(9e)**

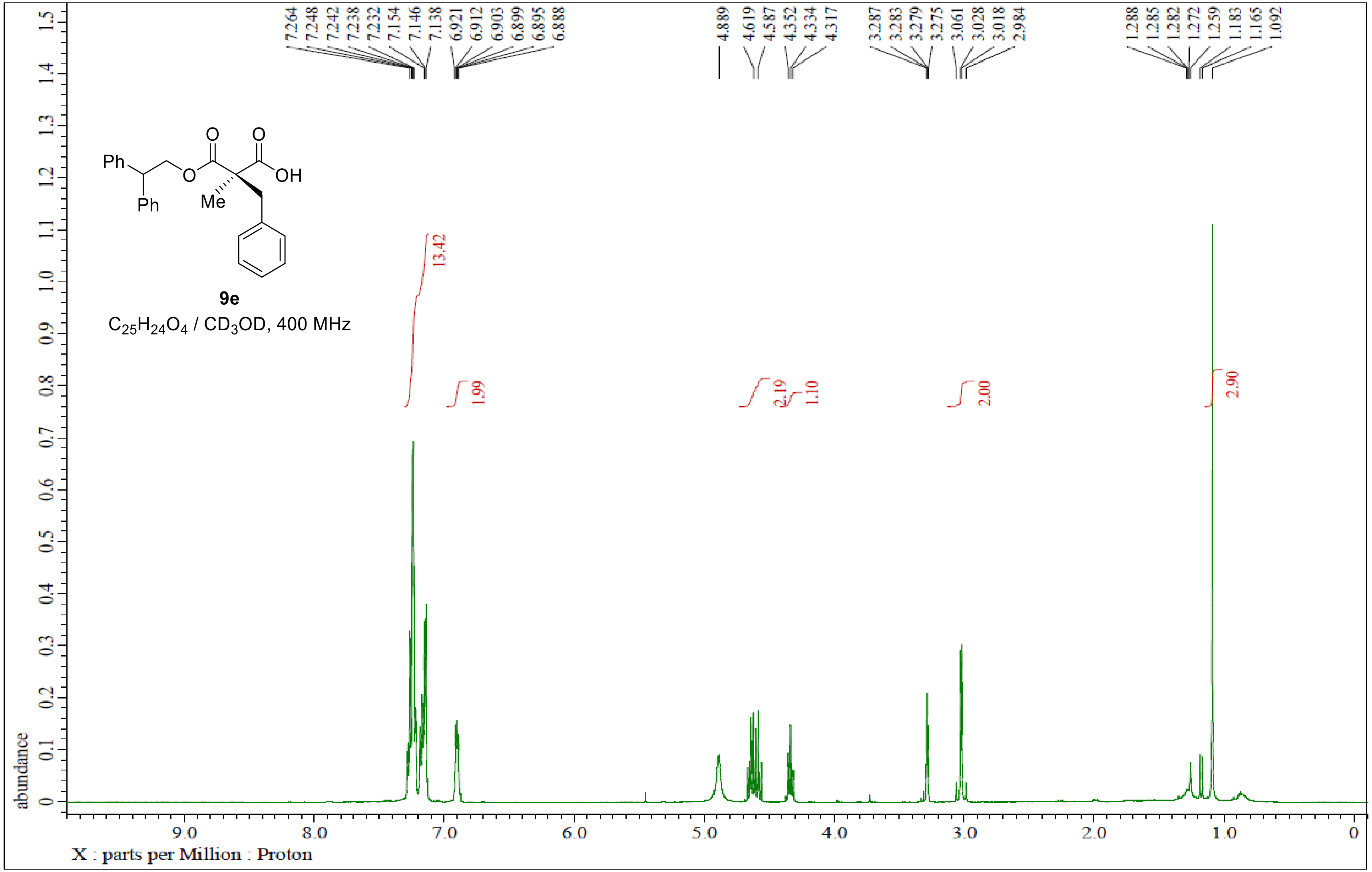

^{13}C-NMR of compound **(9e)**

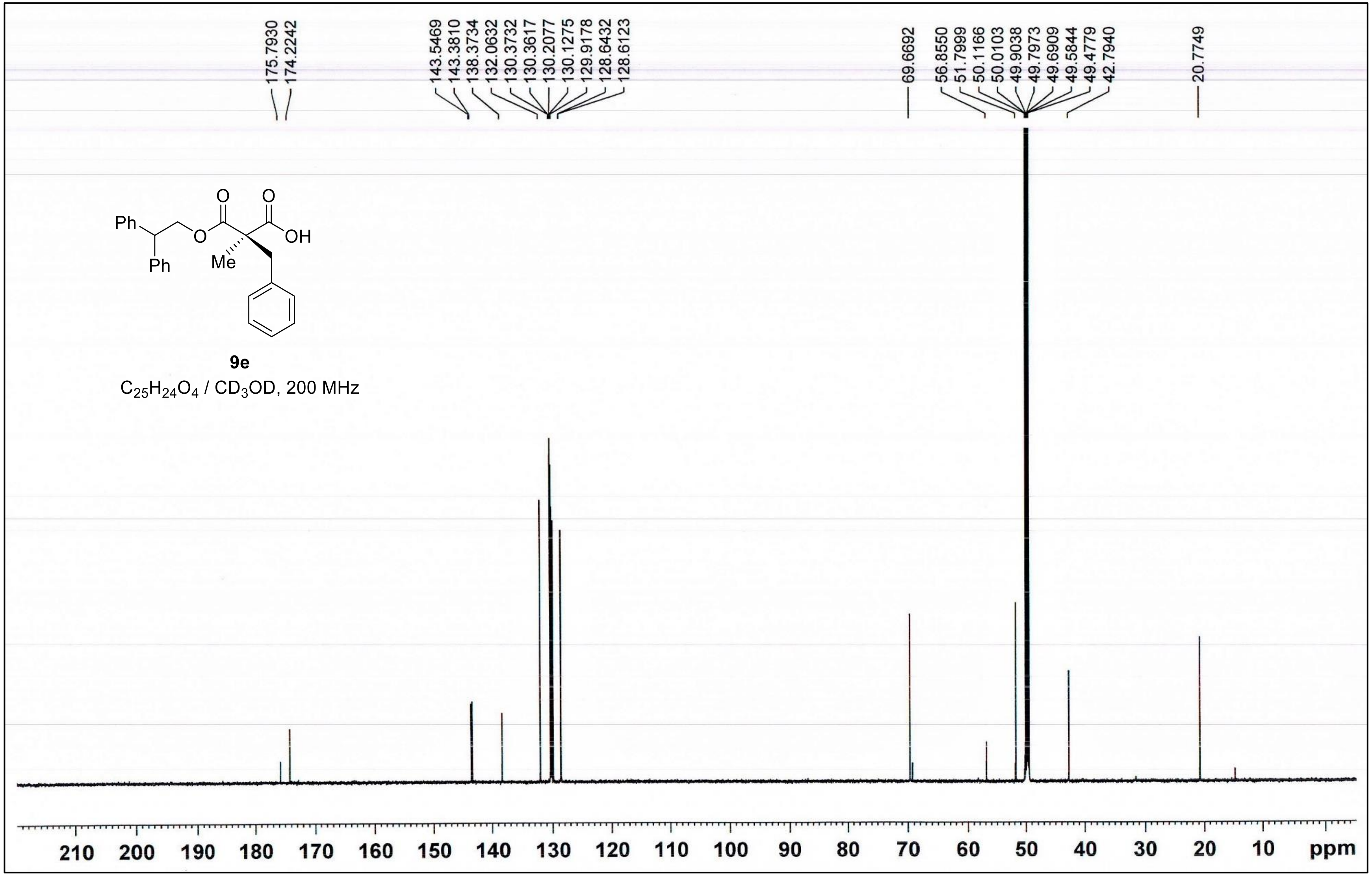

^{1}H-NMR of compound **(10e)**

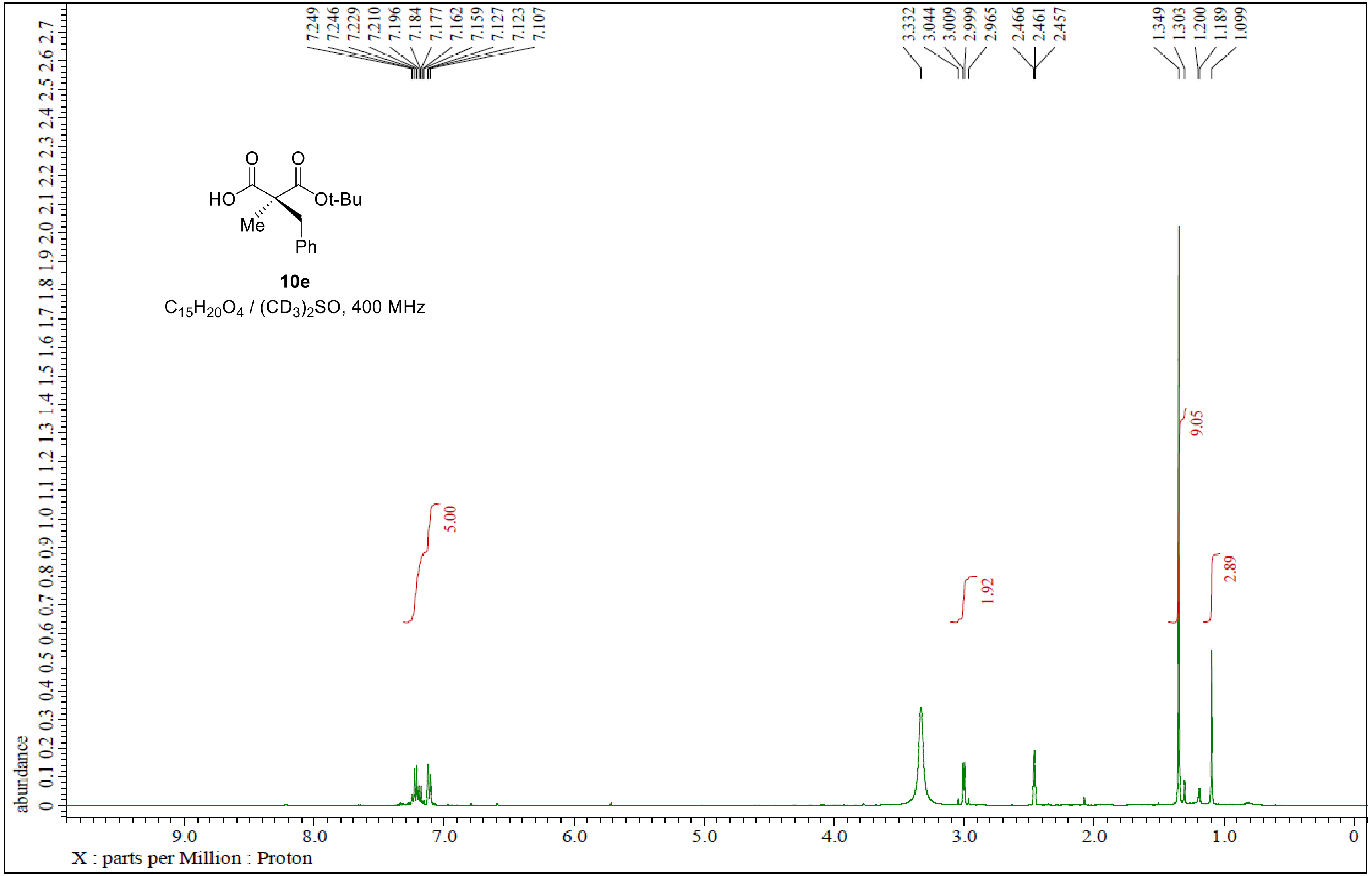

[13]*C-NMR of compound* **(10e)**

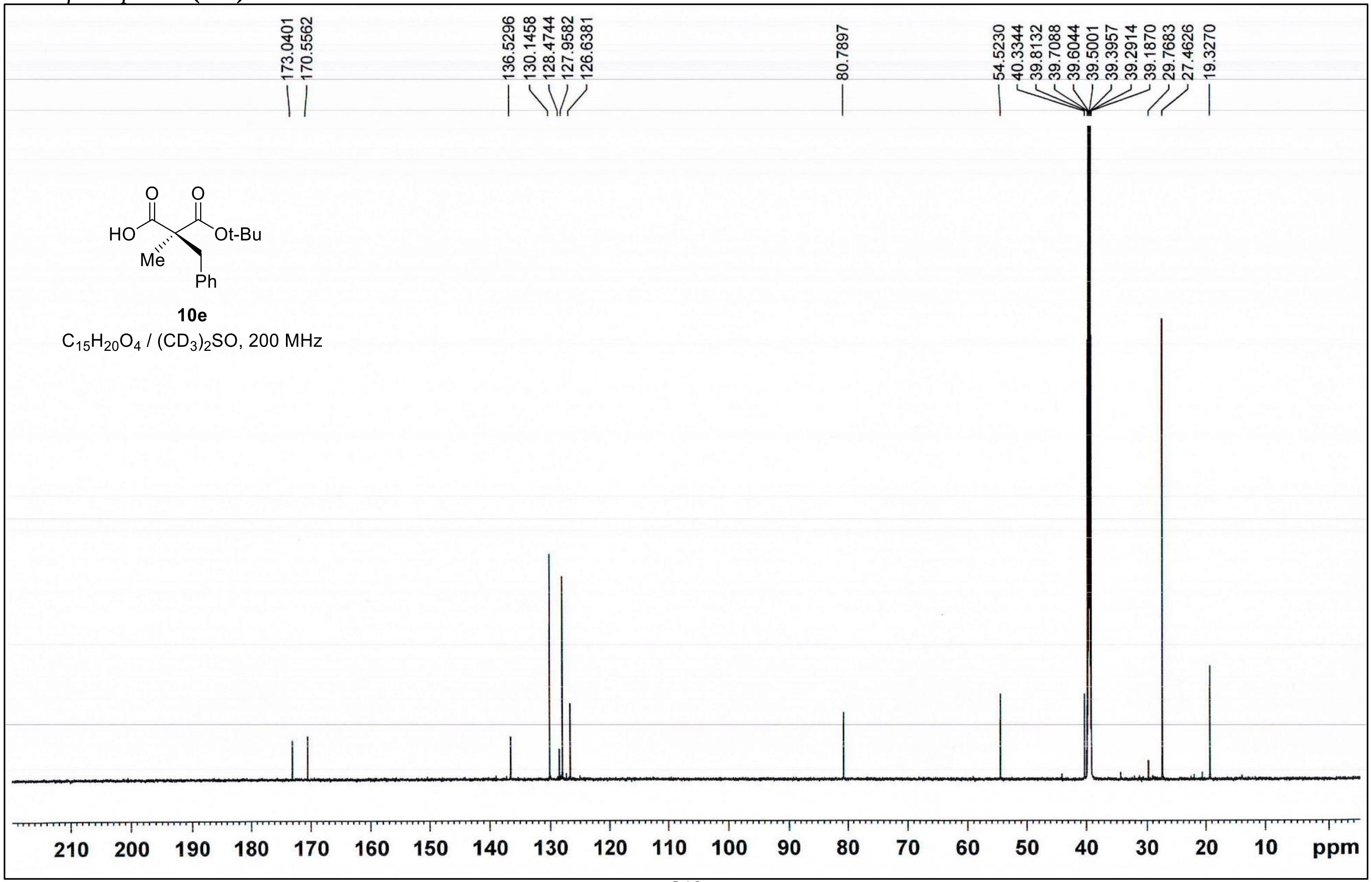

^{1}H-NMR of compound **(10i)**

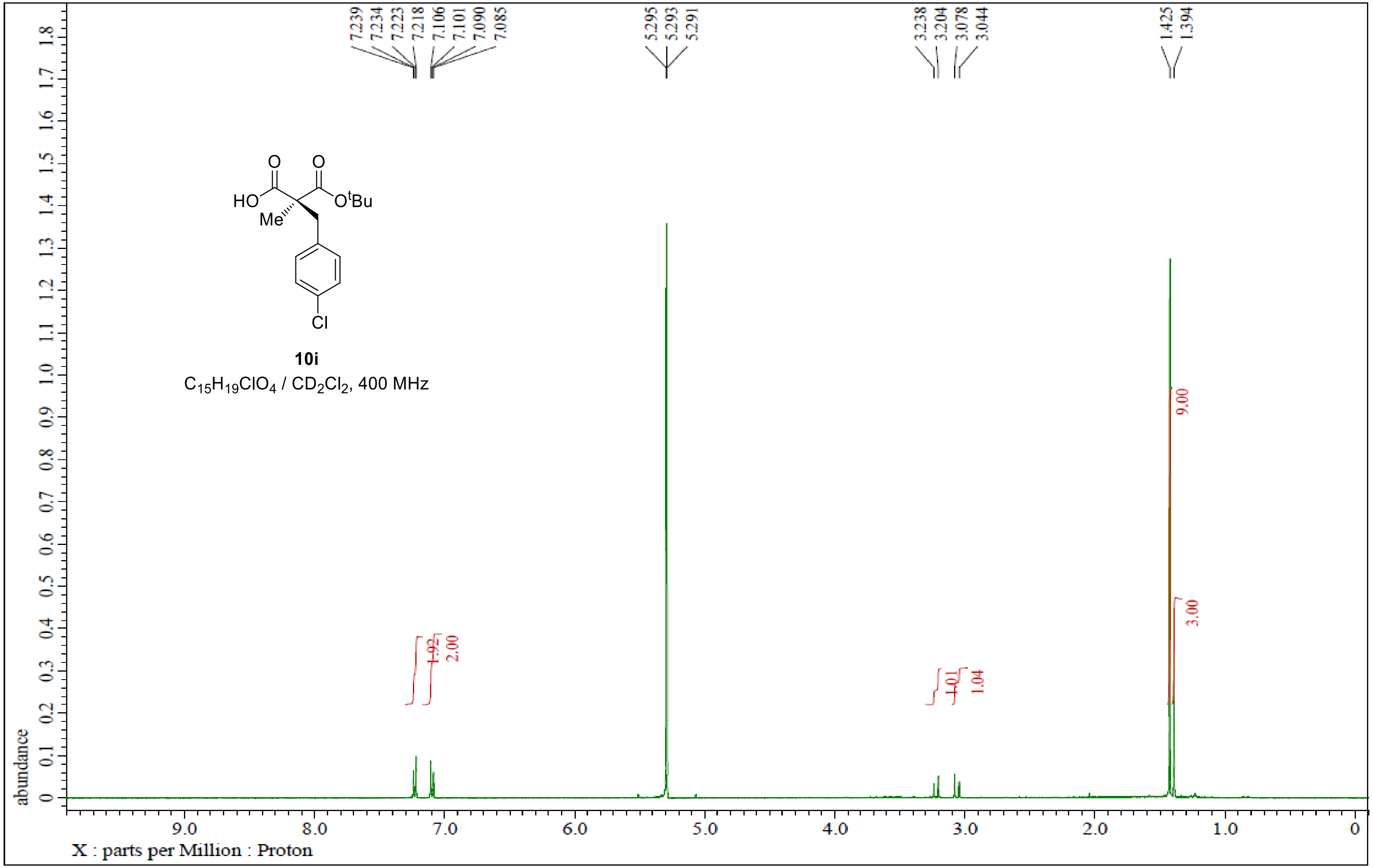

^{13}*C-NMR of compound* **(10i)**

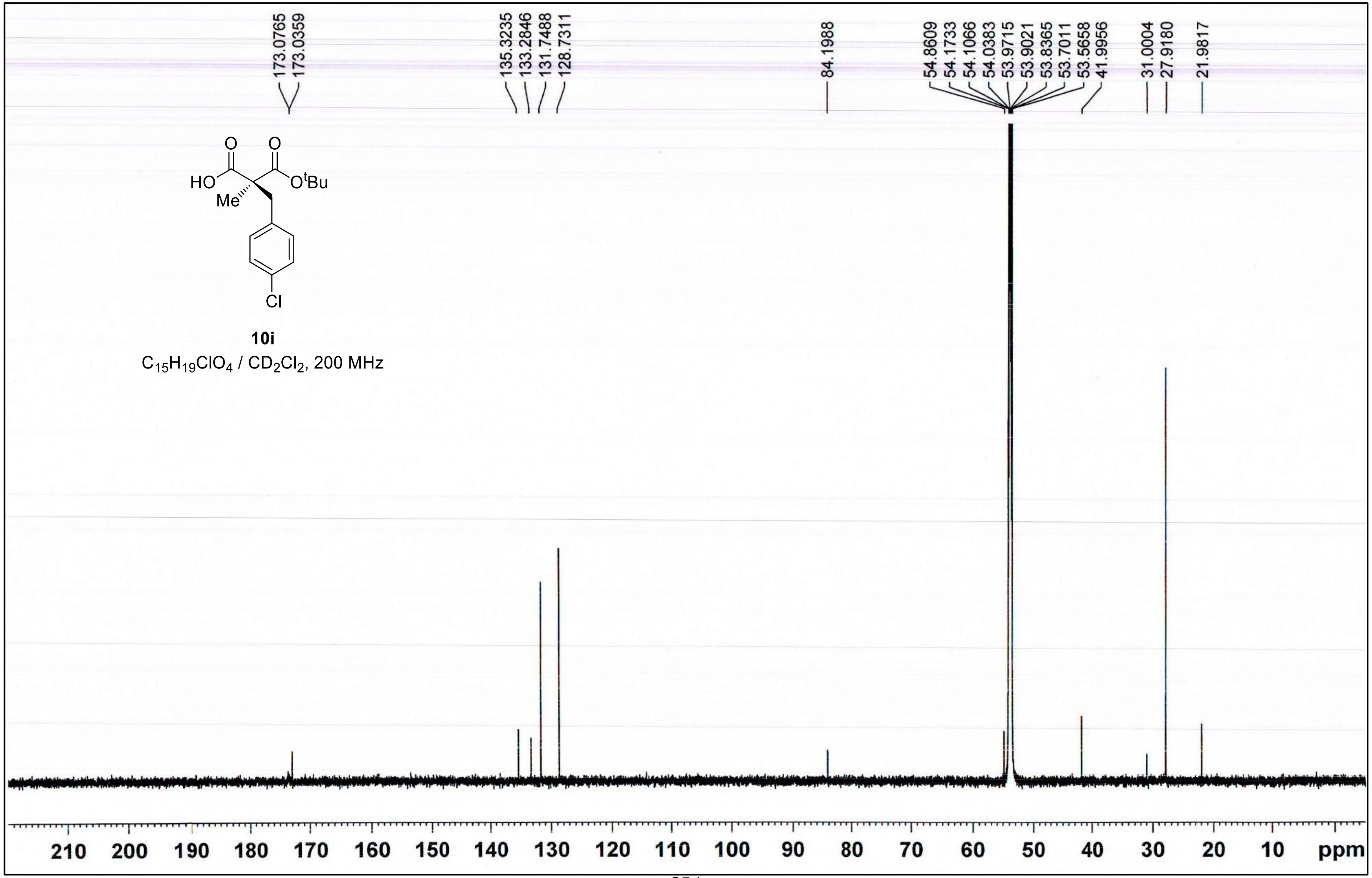

(2) Chiral HPLC Chromatogram

Area Percent Report

Instrument Name: L-2000

Software Version: Version LaChrom 8908800-07

Acquisition Method: Diacel Chiralcel OJ-H, Hexane: 2-Propanol = 99.8 : 0.2,

λ = 256nm, flow rate= 1 mL/min

Sample ID: ***rac*-12**

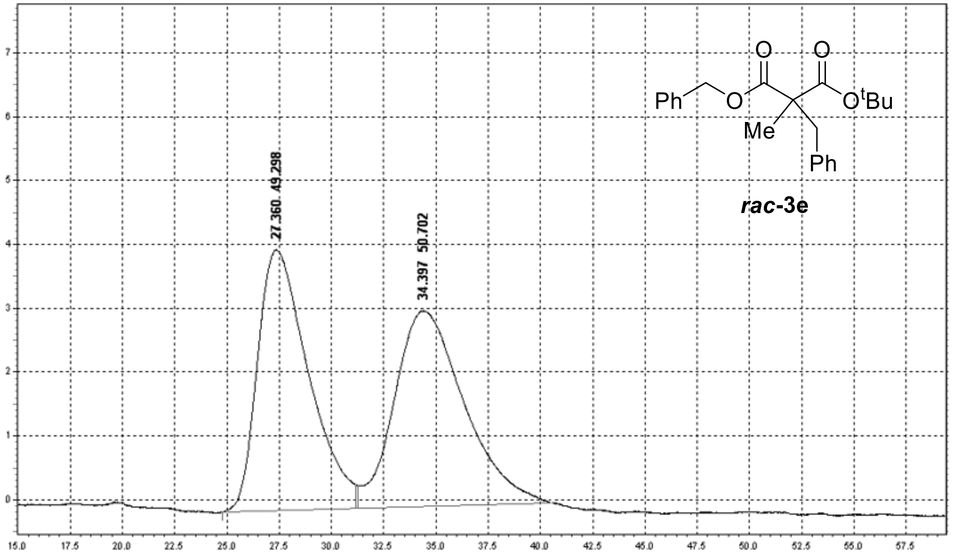

UV Results

Name	Retention Time	Area	Area Percent	Integration Codes
	27.360	2757700	49.298	mm
	34.397	2836239	50.702	mm
Totals		5593939	100.000	

Area Percent Report

Instrument Name: L-2000

Software Version: Version LaChrom 8908800-07

Acquisition Method: Diacel Chiralcel OJ-H, Hexane: 2-Propanol = 99.8 : 0.2,

λ = 256nm, flow rate= 1 mL/min

Sample ID: ***chiral*-12**

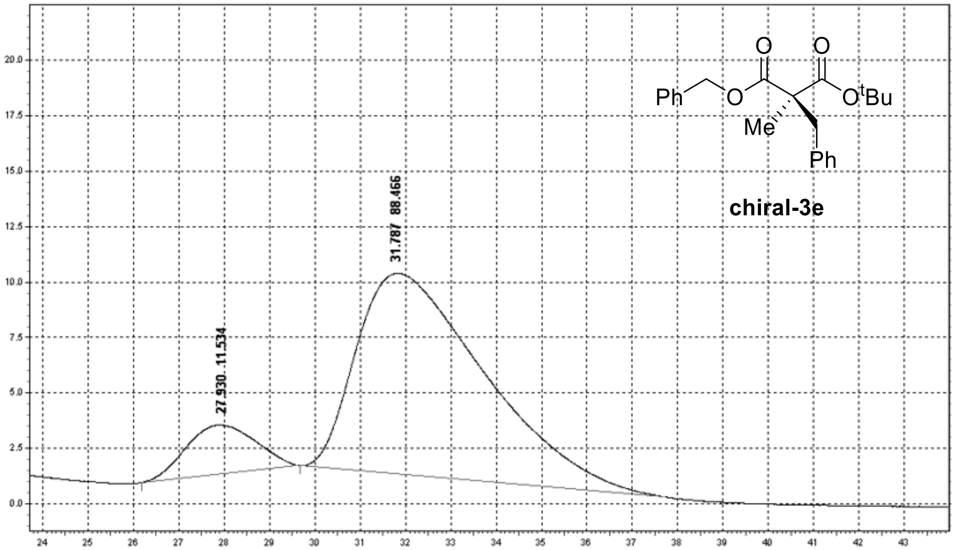

UV Results

Name	Retention Time	Area	Area Percent	Integration Codes
	27.930	926883	11.534	mm
	31.787	7109476	88.466	mm
Totals		8036359	100.000	

Area Percent Report

Instrument Name: L-2000

Software Version: Version LaChrom 8908800-07

Acquisition Method: Diacel Chiralcel OJ-H, Hexane: 2-Propanol = 99.5 : 0.5,

λ = 256nm, flow rate= 1 mL/min

Sample ID: ***rac*-13**

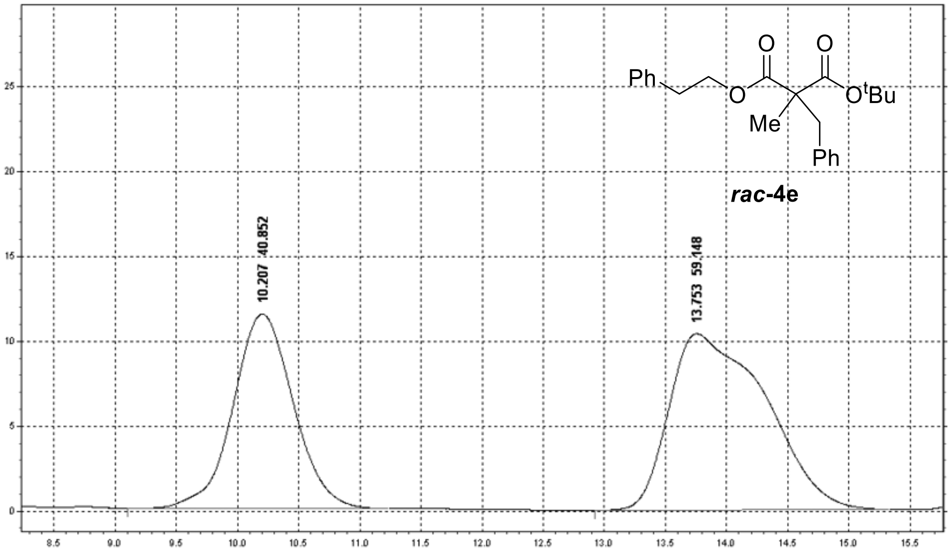

UV Results

Name	Retention Time	Area	Area Percent	Integration Codes
	10.207	1570158	40.929	BI
	13.753	2266120	59.071	II

Totals				
		3836278	100.000	

Area Percent Report

Instrument Name: L-2000

Software Version: Version LaChrom 8908800-07

Acquisition Method: Diacel Chiralcel OJ-H, Hexane: 2-Propanol = 99.5 : 0.5,

λ = 256nm, flow rate= 1 mL/min

Sample ID: ***chiral*-13**

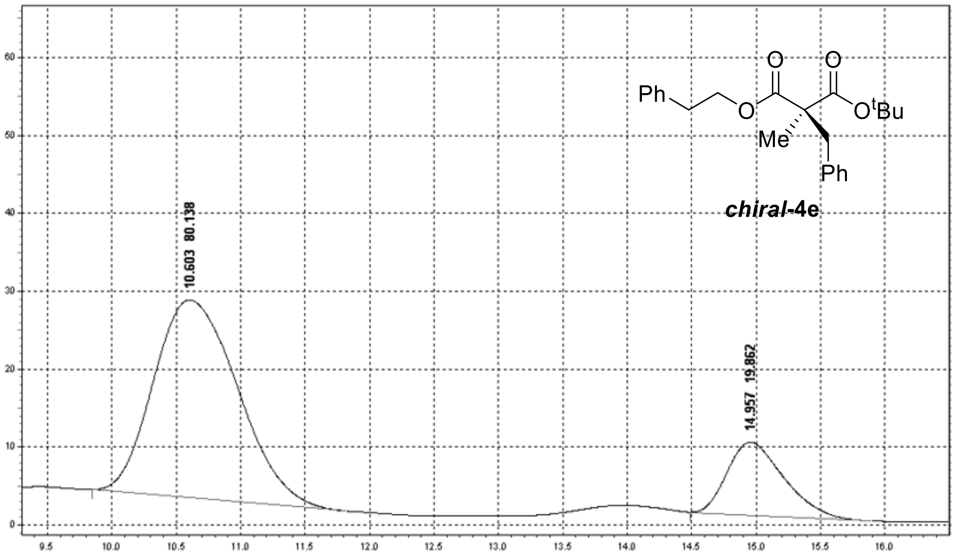

UV Results

Name	Retention Time	Area	Area Percent	Integration Codes
	10.603	4621474	80.138	BI
	14.957	1145455	19.862	BI

Totals				
		5766929	100.000	

Area Percent Report

Instrument Name: L-2000

Software Version: Version LaChrom 8908800-07

Acquisition Method: Diacel Chiralcel OJ-H, Hexane: 2-Propanol = 99.5 : 0.5,

λ = 256nm, flow rate= 1 mL/min

Sample ID: ***rac*-14**

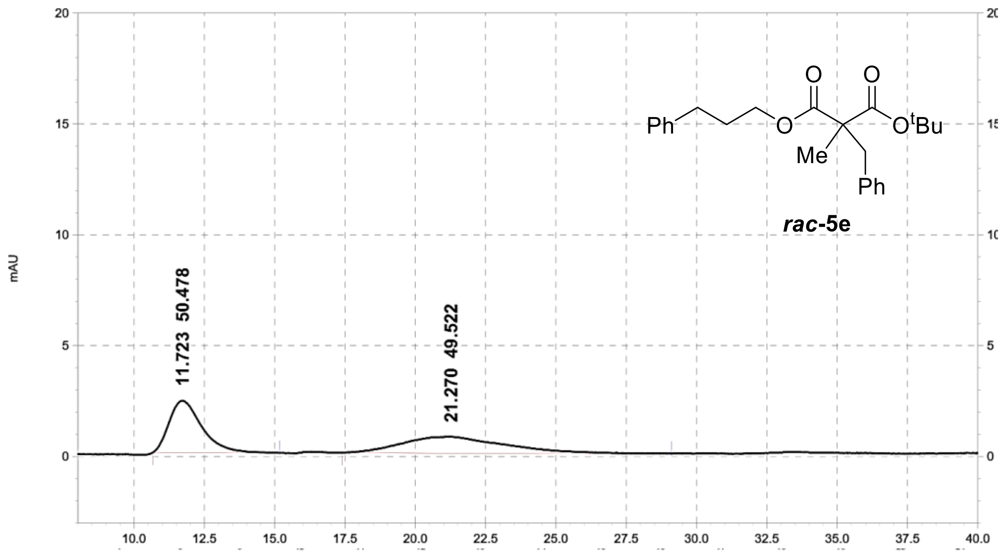

UV Results

Name	Retention Time	Area	Area Percent	Integration Codes
	11.723	767109	50.478	mm
	21.270	752566	49.522	MM

Totals				
		1519675	100.000	

Area Percent Report

Instrument Name: L-2000

Software Version: Version LaChrom 8908800-07

Acquisition Method: Diacel Chiralcel OJ-H, Hexane: 2-Propanol = 99.5 : 0.5,

λ = 256nm, flow rate= 1 mL/min

Sample ID: ***chiral*-14**

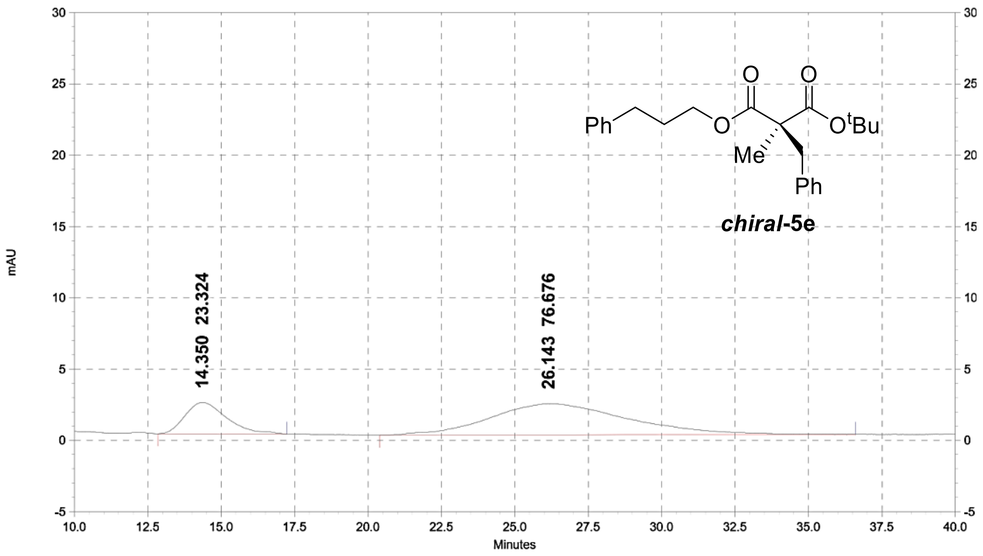

UV Results

Name	Retention Time	Area	Area Percent	Integration Codes
	14.350	874433	23.324	MM
	26.143	2874629	76.676	MM

Totals				
		3749062	100.000	

Area Percent Report

Instrument Name: L-2000

Software Version: Version LaChrom 8908800-07

Acquisition Method: Diacel Chiralcel OJ-H, Hexane: 2-Propanol = 99.5 : 0.5,

λ = 256nm, flow rate= 1 mL/min

Sample ID: ***rac*-11**

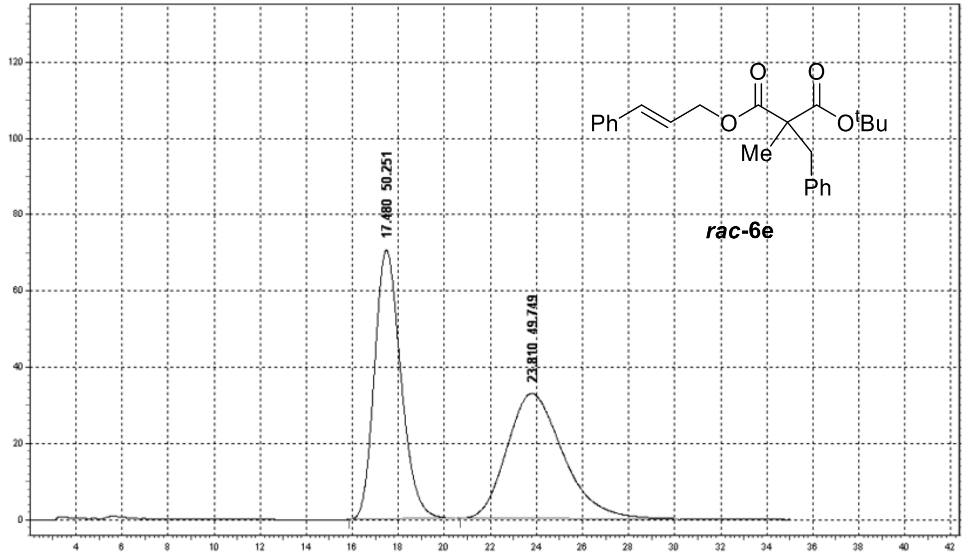

UV Results

Name	Retention Time	Area	Area Percent	Integration Codes
	17.480	22433238	50.251	MM
	23.810	22209490	49.749	mm
Totals		44642728	100.000	

Area Percent Report

Instrument Name: L-2000

Software Version: Version LaChrom 8908800-07

Acquisition Method: Diacel Chiralcel OJ-H, Hexane: 2-Propanol = 99.5 : 0.5,

λ = 256nm, flow rate= 1 mL/min

Sample ID: ***chiral*-11**

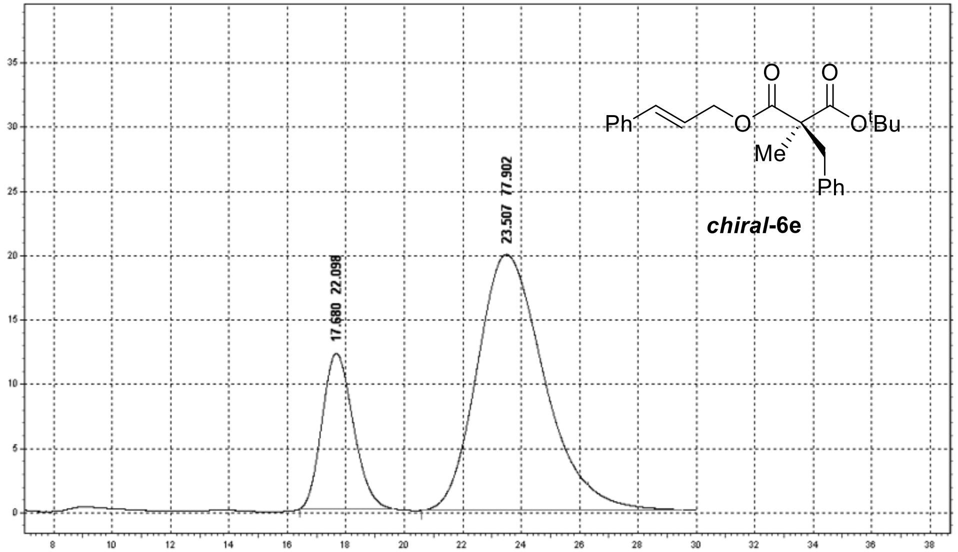

UV Results

Name	Retention Time	Area	Area Percent	Integration Codes
	17.680	3594423	22.098	MM
	23.507	12671615	77.902	mm
Totals		16266038	100.000	

Area Percent Report

Instrument Name: L-2000

Software Version: Version LaChrom 8908800-07

Acquisition Method: Diacel Chiralpak AD-H, Hexane: 2-Propanol = 800 : 1,

λ = 220nm, flow rate= 1 mL/min

Sample ID: ***rac*-10a**

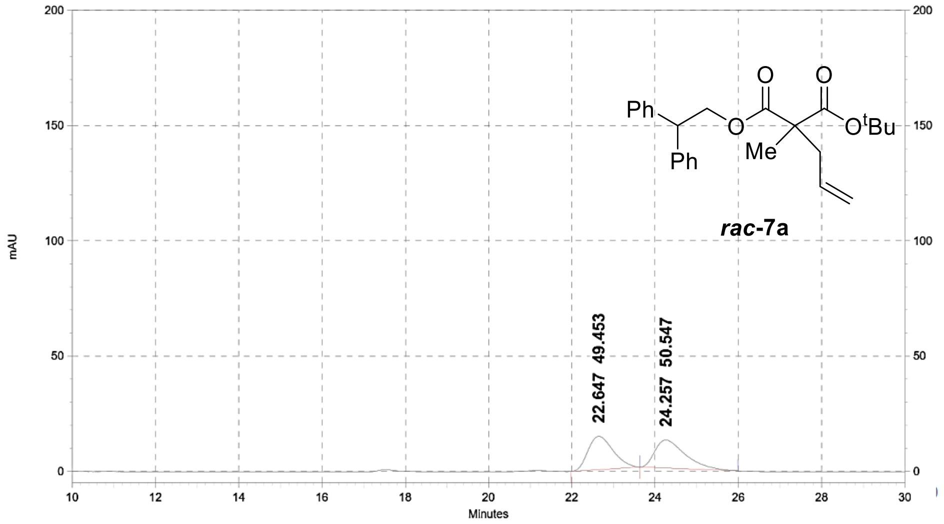

UV Results

Name	Retention Time	Area	Area Percent	Integration Codes
	22.647	2415842	49.453	MM
	24.257	2469259	50.547	MM

Totals				
		4885101	100.000	

Area Percent Report

Instrument Name: Waters 2489

Software Version: Breeze 2 HPLC System 6.20.00.00

Acquisition Method: Diacel Chiralpak AD-H, Hexane: 2-Propanol = 800 : 1,

λ = 220nm, flow rate= 1 mL/min

Sample ID: ***chiral*-10a**

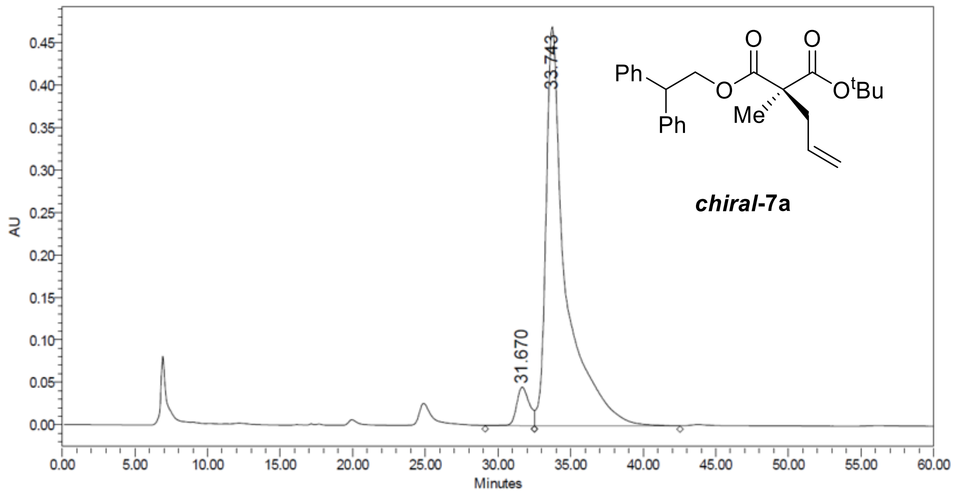

	RT	Area	% Area	Height
1	31.670	2748065	5.82	45323
2	33.743	44479806	94.18	469755

Area Percent Report

Instrument Name: L-2000

Software Version: Version LaChrom 8908800-07

Acquisition Method: Diacel Chiralpak AD-H, Hexane: 2-Propanol = 800 : 1,

λ = 220nm, flow rate= 1 mL/min

Sample ID: ***rac*-10b**

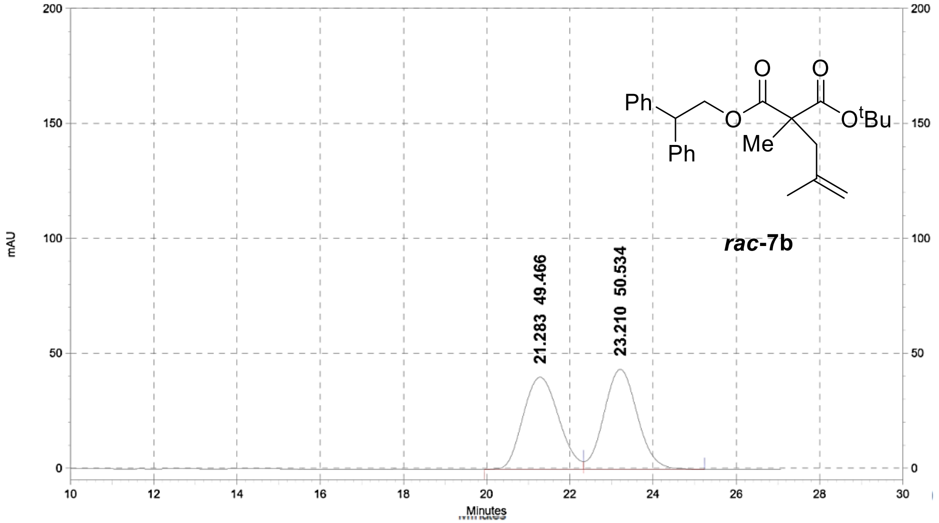

UV Results

Name	Retention Time	Area	Area Percent	Integration Codes
	21.283	9441092	49.466	BV
	23.210	9644869	50.534	VB

Totals		19085961	100.000	

Area Percent Report

Instrument Name: Waters 2489

Software Version: Breeze 2 HPLC System 6.20.00.00

Acquisition Method: Diacel Chiralpak AD-H, Hexane: 2-Propanol = 800 : 1,

λ = 220nm, flow rate= 1 mL/min

Sample ID: ***chiral*-10b**

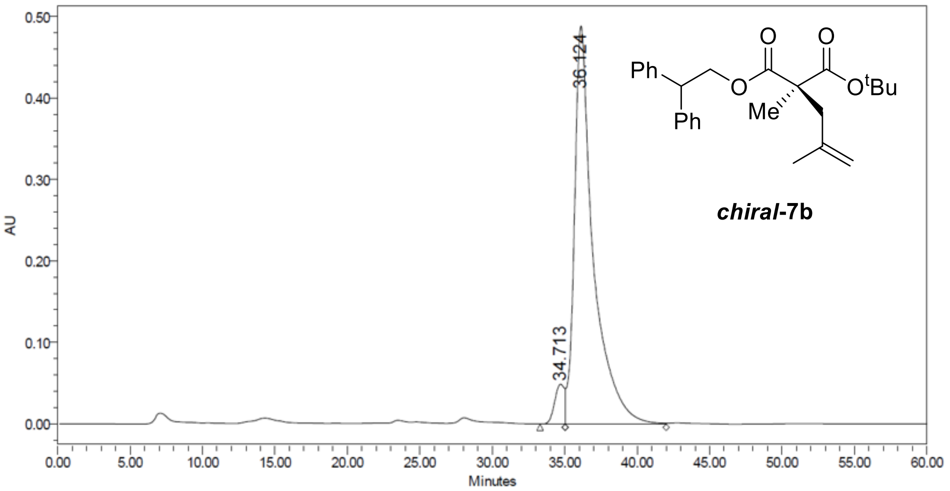

	RT	Area	% Area	Height
1	34.713	2250875	4.84	48913
2	36.124	44299373	95.16	488475

Area Percent Report

Instrument Name: L-2000

Software Version: Version LaChrom 8908800-07

Acquisition Method: Diacel Chiralpak AD-H, Hexane: 2-Propanol = 99 : 1,

λ = 256nm, flow rate= 1 mL/min

Sample ID: ***rac*-10c**

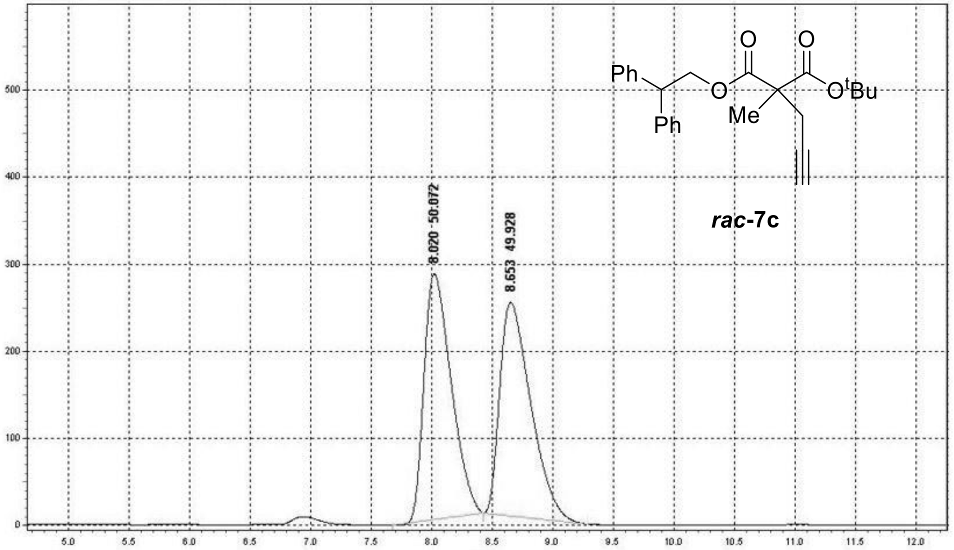

UV Results

Name	Retention Time	Area	Area Percent	Integration Codes
	8.020	17528174	50.072	mm
	8.653	17477897	49.928	BI

Totals				
		35006071	100.000	

Area Percent Report

Instrument Name: L-2000

Software Version: Version LaChrom 8908800-07

Acquisition Method: Diacel Chiralpak AD-H, Hexane: 2-Propanol = 99 : 1,

λ = 256nm, flow rate= 1 mL/min

Sample ID: ***chiral*-10c**

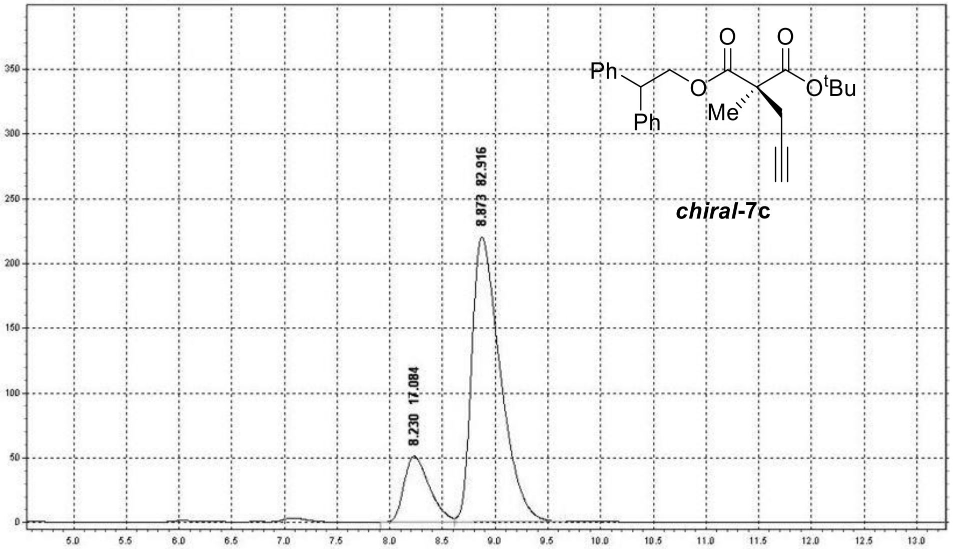

UV Results

Name	Retention Time	Area	Area Percent	Integration Codes
	8.230	3417932	17.084	BV
	8.873	16588218	82.916	VB

Totals				
		20006150	100.000	

Area Percent Report

Instrument Name: L-2000

Software Version: Version LaChrom 8908800-07

Acquisition Method: Diacel Chiralcel OD-H, Hexane: 2-Propanol = 99 : 1,

λ = 220nm, flow rate= 1 mL/min

Sample ID: ***rac*-10d**

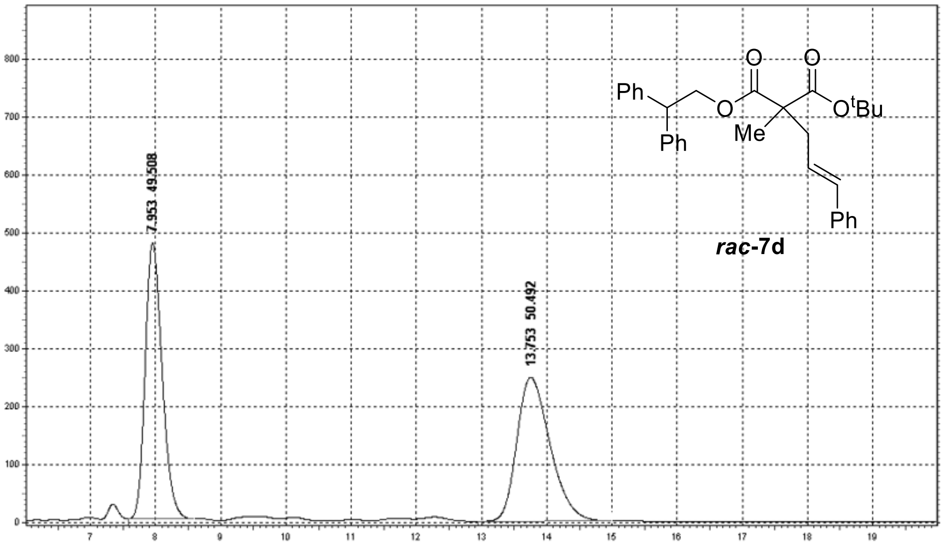

UV Results

Name	Retention Time	Area	Area Percent	Integration Codes
	7.953	34412104	49.508	IB
	13.753	35095980	50.492	BB

Totals				
		69508084	100.000	

Area Percent Report

Instrument Name: L-2000

Software Version: Version LaChrom 8908800-07

Acquisition Method: Diacel Chiralcel OD-H, Hexane: 2-Propanol = 99 : 1,

λ = 220nm, flow rate= 1 mL/min

Sample ID: ***chiral*-10d**

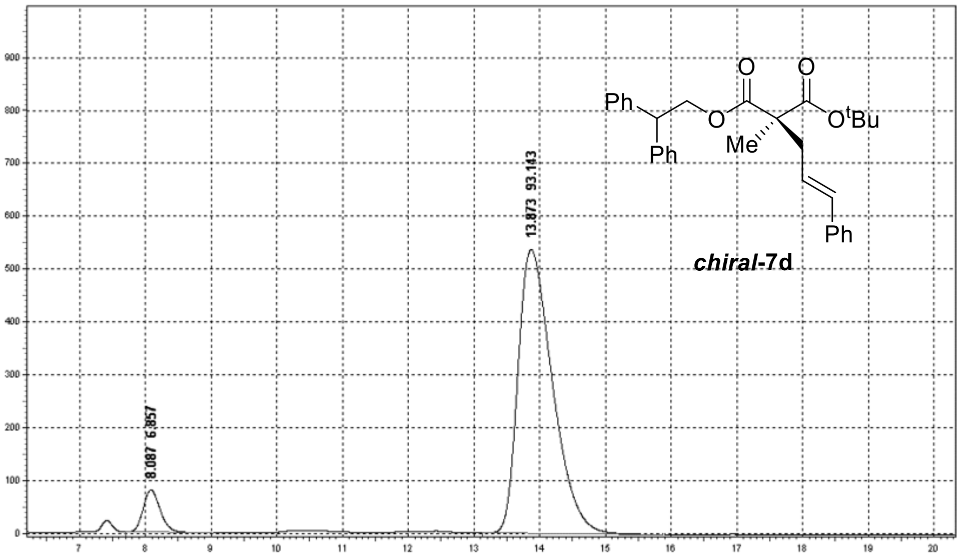

UV Results

Name	Retention Time	Area	Area Percent	Integration Codes
	8.087	5776775	6.857	BB
	13.873	78466467	93.143	BI

Totals				
		84243242	100.000	

Area Percent Report

Instrument Name: L-2000

Software Version: Version LaChrom 8908800-07

Acquisition Method: Diacel Chiralcel OJ-H, Hexane: 2-Propanol = 95 : 5,

λ = 256nm, flow rate= 1 mL/min

Sample ID: ***rac*-10e**

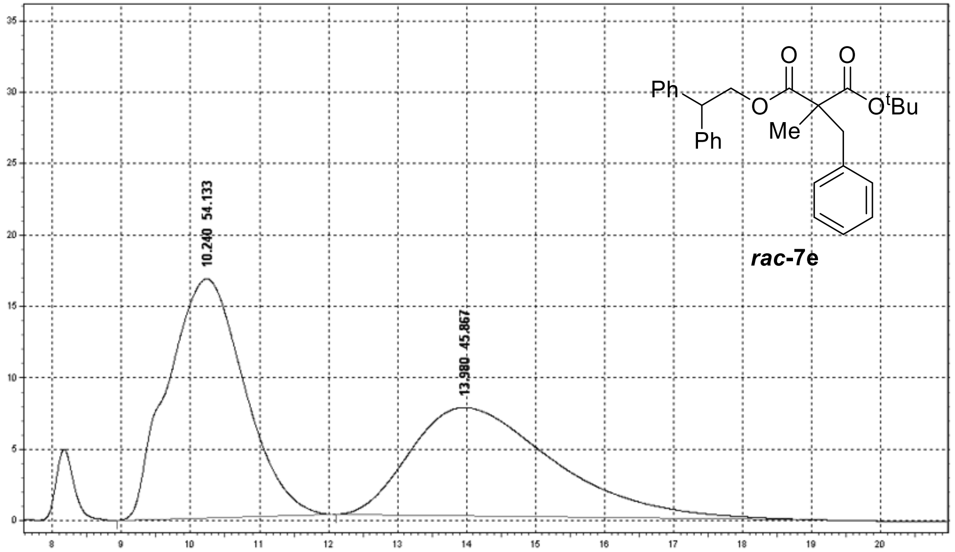

UV Results

Name	Retention Time	Area	Area Percent	Integration Codes
	10.240	5154938	54.133	BB
	13.980	4367747	45.867	mm
Totals		9522685	100.000	

Area Percent Report

Instrument Name: L-2000

Software Version: Version LaChrom 8908800-07

Acquisition Method: Diacel Chiralcel OJ-H, Hexane: 2-Propanol = 95 : 5,

λ = 256nm, flow rate= 1 mL/min

Sample ID: ***chiral*-10e**

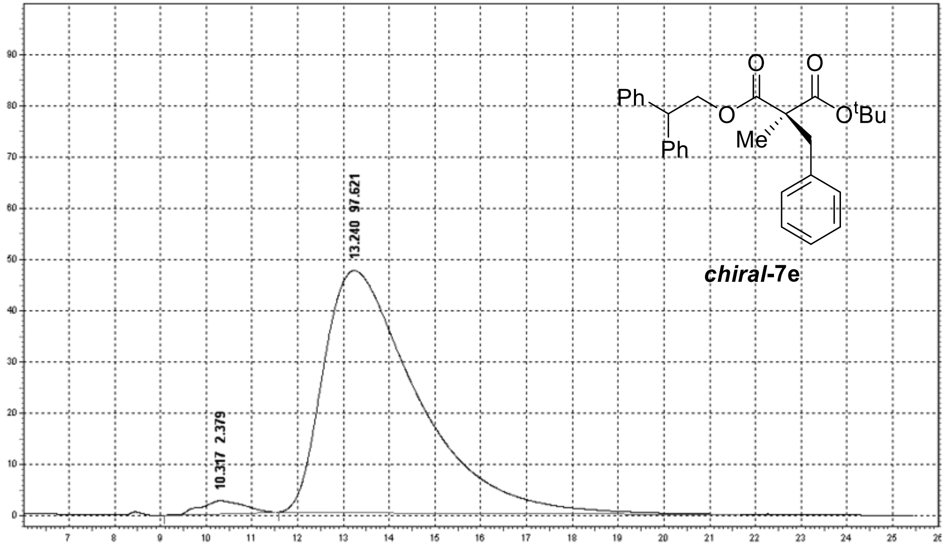

UV Results

Name	Retention Time	Area	Area Percent	Integration Codes
	10.317	656208	2.379	IB
	13.240	26925144	97.621	MM
Totals		27581352	100.000	

Area Percent Report

Instrument Name: L-2000

Software Version: Version LaChrom 8908800-07

Acquisition Method: Diacel Chiralcel OD-H, Hexane: 2-Propanol = 200 : 1,

λ = 220nm, flow rate= 1 mL/min

Sample ID: ***rac*-10f**

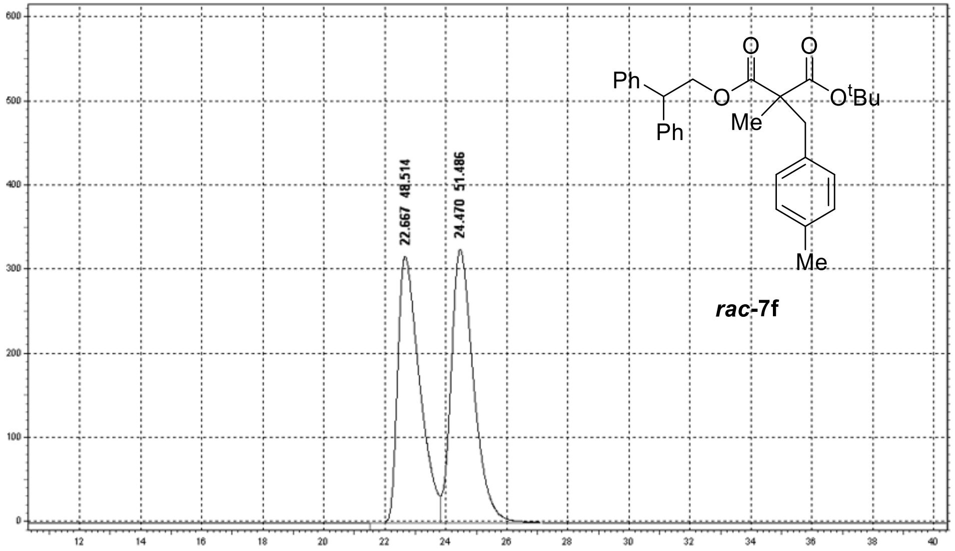

UV Results

Name	Retention Time	Area	Area Percent	Integration Codes
	22.667	62846610	48.514	IV
	24.470	66696530	51.486	VI

Totals				
		129543140	100.000	

Area Percent Report

Instrument Name: L-2000

Software Version: Version LaChrom 8908800-07

Acquisition Method: Diacel Chiralcel OD-H, Hexane: 2-Propanol = 200 : 1,

λ = 220nm, flow rate= 1 mL/min

Sample ID: ***chiral*-10f**

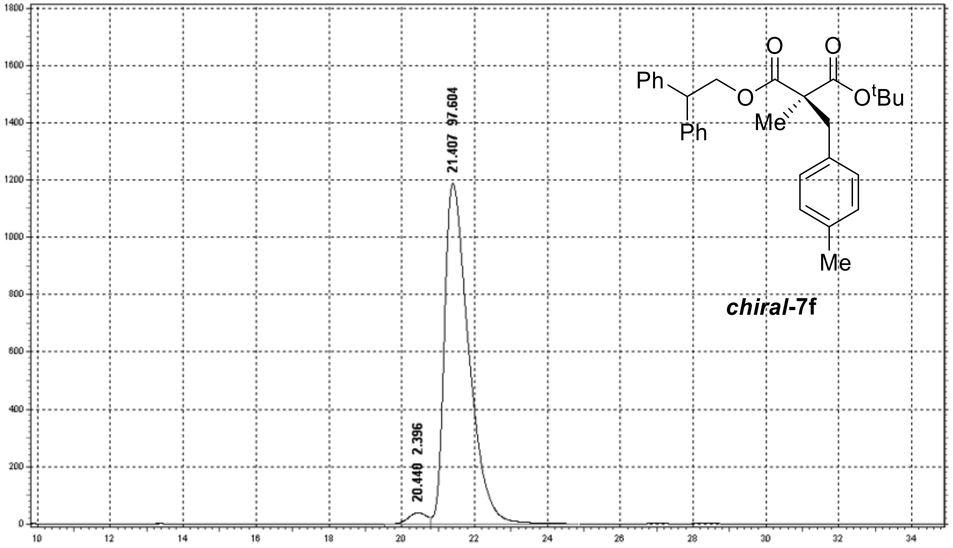

UV Results

Name	Retention Time	Area	Area Percent	Integration Codes
	20.440	5386852	2.396	BV
	21.407	219484204	97.604	VI

Totals				
		224871056	100.000	

Area Percent Report

Instrument Name: L-2000

Software Version: Version LaChrom 8908800-07

Acquisition Method: Diacel Chiralcel OJ-H, Hexane: 2-Propanol = 99 : 1,

λ = 256nm, flow rate= 1 mL/min

Sample ID: ***rac*-10g**

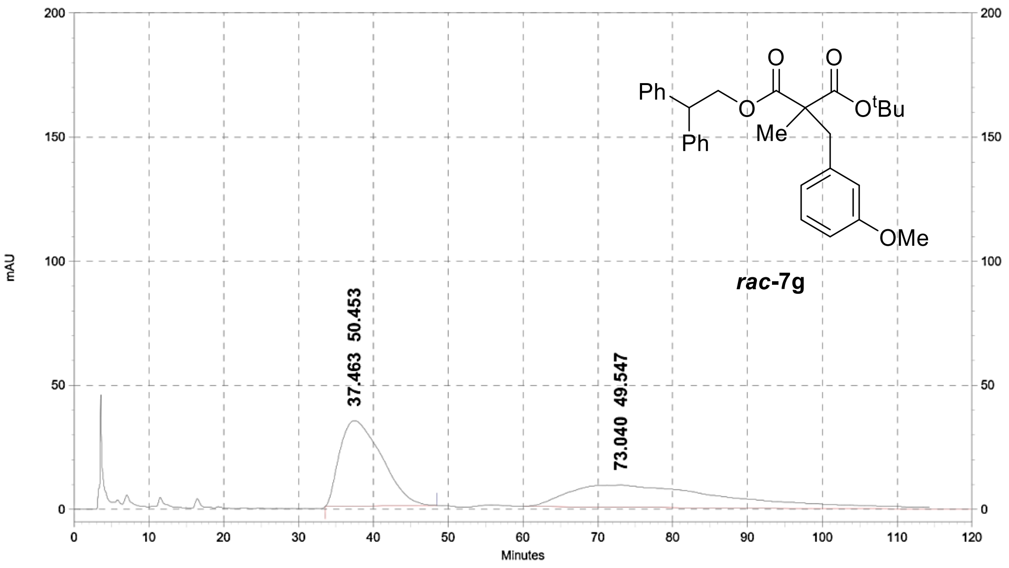

UV Results

Name	Retention Time	Area	Area Percent	Integration Codes
	37.463	53676838	50.453	mm
	73.040	52712644	49.547	mm

Totals				
		106389482	100.000	

Area Percent Report

Instrument Name: L-2000

Software Version: Version LaChrom 8908800-07

Acquisition Method: Diacel Chiralcel OJ-H, Hexane: 2-Propanol = 99 : 1,

λ = 256nm, flow rate= 1 mL/min

Sample ID: ***chiral*-10g**

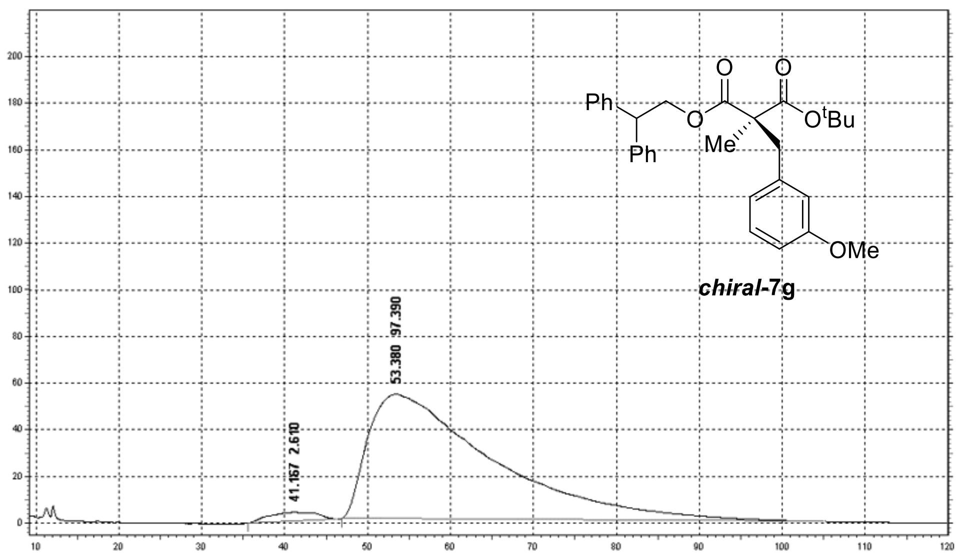

UV Results

Name	Retention Time	Area	Area Percent	Integration Codes
	41.167	6007492	2.610	MM
	53.380	224127019	97.390	MM

Totals				
		230134511	100.000	

Area Percent Report

Instrument Name: L-2000

Software Version: Version LaChrom 8908800-07

Acquisition Method: Diacel Chiralcel OJ-H, Hexane: 2-Propanol = 99 : 1,

λ = 220nm, flow rate= 1 mL/min

Sample ID: ***rac*-10h**

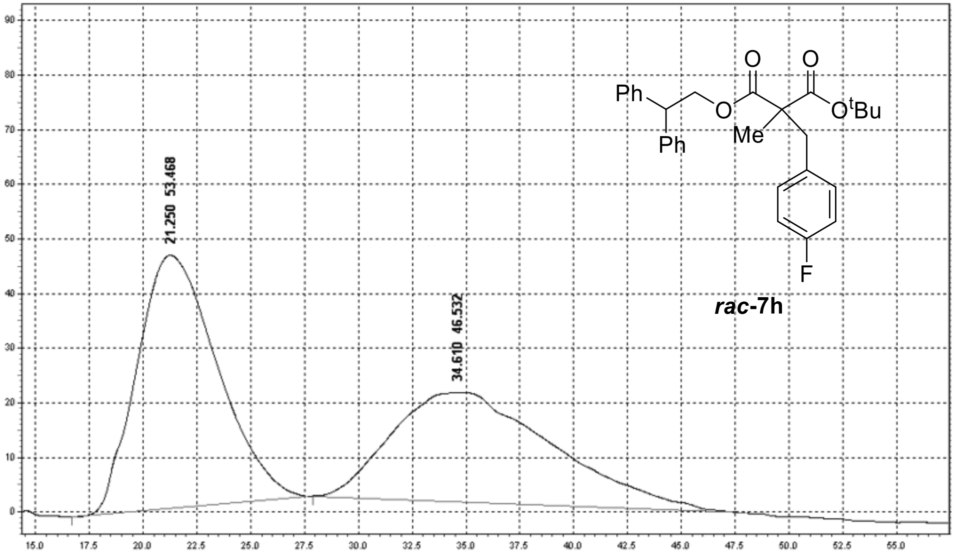

UV Results

Name	Retention Time	Area	Area Percent	Integration Codes
	21.250	48052028	53.468	mm
	34.610	41818871	46.532	mm
Totals		89870899	100.000	

Area Percent Report

Instrument Name: L-2000

Software Version: Version LaChrom 8908800-07

Acquisition Method: Diacel Chiralcel OJ-H, Hexane: 2-Propanol = 99 : 1,

λ = 220nm, flow rate= 1 mL/min

Sample ID: ***chiral*-10h**

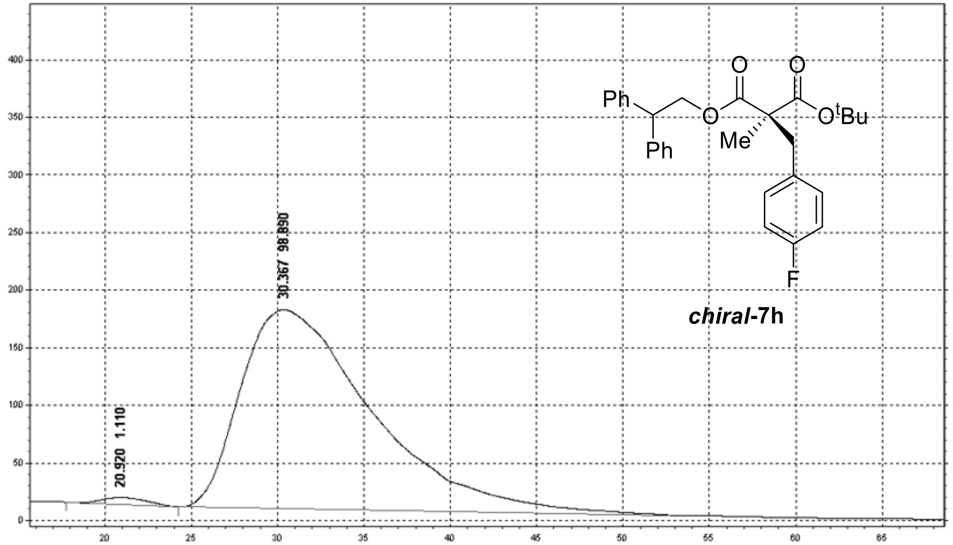

UV Results

Name	Retention Time	Area	Area Percent	Integration Codes
	20.920	4105410	1.110	mm
	30.367	365895950	98.890	mm
Totals		370001360	100.000	

Area Percent Report

Instrument Name: L-2000

Software Version: Version LaChrom 8908800-07

Acquisition Method: Diacel Chiralpak AD-H, Hexane: 2-Propanol = 200 : 1,

λ = 220nm, flow rate= 1 mL/min

Sample ID: ***rac*-10i**

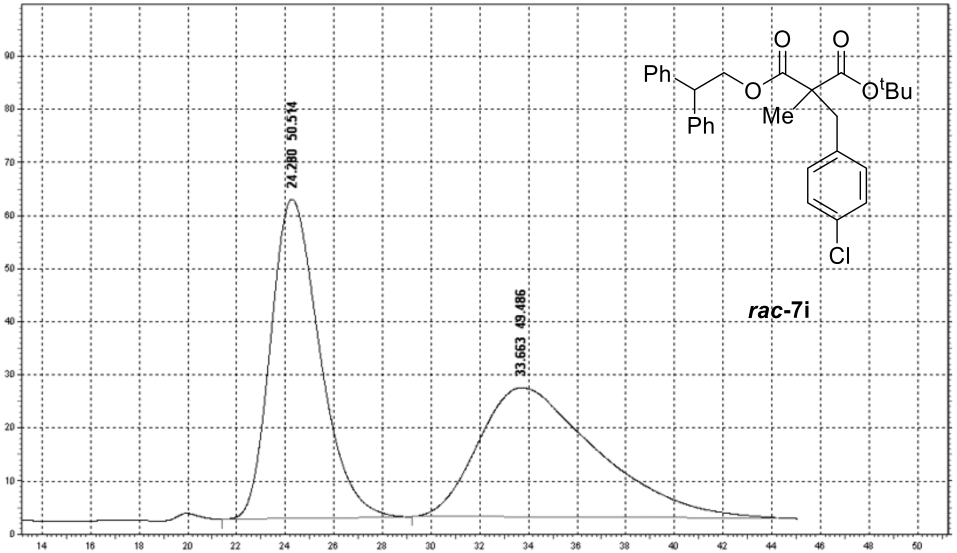

UV Results

Name	Retention Time	Area	Area Percent	Integration Codes
	24.280	33582728	50.514	MM
	33.663	32899664	49.486	mm
Totals		66482392	100.000	

Area Percent Report

Instrument Name: L-2000

Software Version: Version LaChrom 8908800-07

Acquisition Method: Diacel Chiralpak AD-H, Hexane: 2-Propanol = 200 : 1,

λ = 220nm, flow rate= 1 mL/min

Sample ID: ***chiral*-10i**

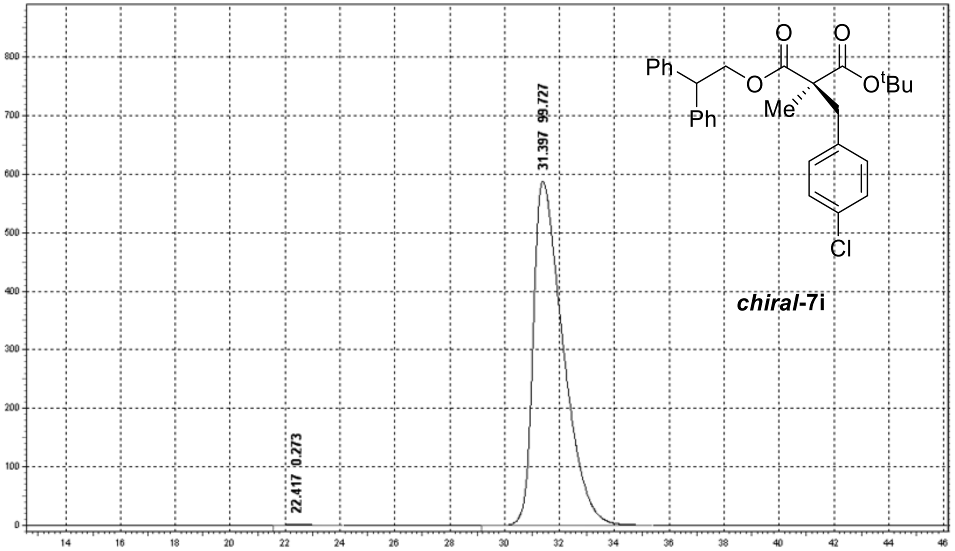

UV Results

Name	Retention Time	Area	Area Percent	Integration Codes
	22.417	485192	0.273	BI
	31.397	177109755	99.727	mm
Totals		177594947	100.000	

Area Percent Report

Instrument Name: L-2000

Software Version: Version LaChrom 8908800-07

Acquisition Method: Diacel Chiralcel OD-H, Hexane: 2-Propanol = 200 : 1,

λ = 220nm, flow rate= 1 mL/min

Sample ID: ***rac*-10j**

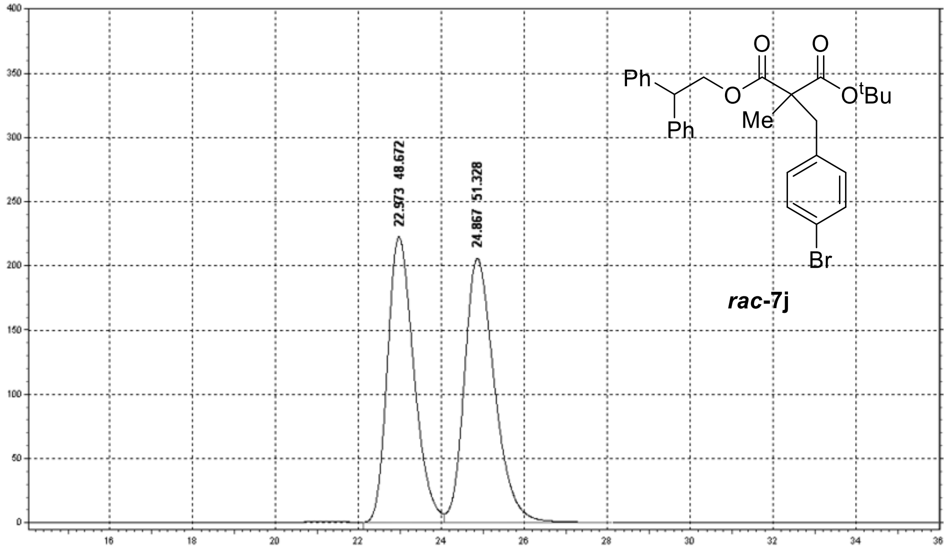

UV Results

Name	Retention Time	Area	Area Percent	Integration Codes
	22.973	39290527	48.672	IV
	24.867	41434527	51.328	VI
Totals		80725054	100.000	

Area Percent Report

Instrument Name: L-2000

Software Version: Version LaChrom 8908800-07

Acquisition Method: Diacel Chiralcel OD-H, Hexane: 2-Propanol = 200 : 1,

λ = 220nm, flow rate= 1 mL/min

Sample ID: ***chiral*-10j**

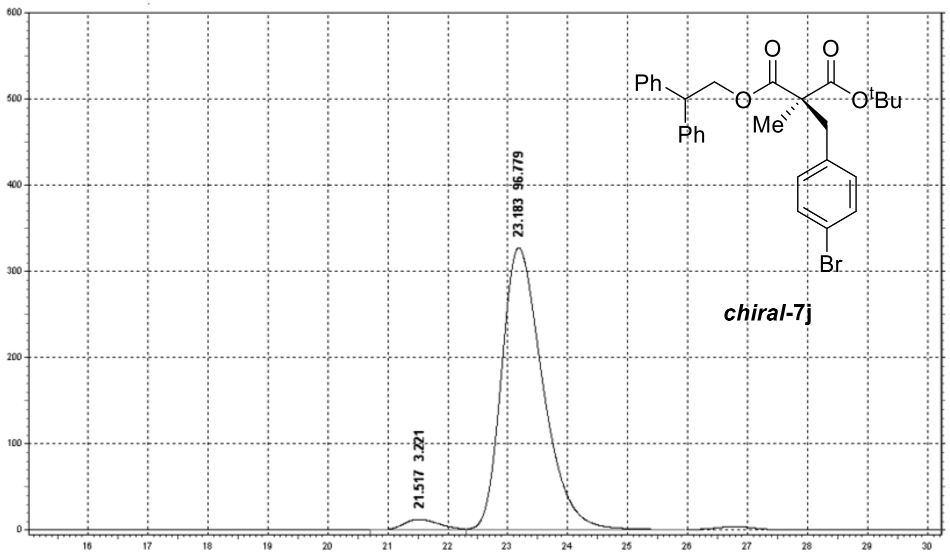

UV Results

Name	Retention Time	Area	Area Percent	Integration Codes
	21.517	2076868	3.221	BV
	23.183	62394964	96.779	VB
Totals		64471832	100.000	

Area Percent Report

Instrument Name: L-2000

Software Version: Version LaChrom 8908800-07

Acquisition Method: Diacel Chiralpak AD-H, Hexane: 2-Propanol = 99 : 1,

λ = 256nm, flow rate= 1 mL/min

Sample ID: ***rac*-10k**

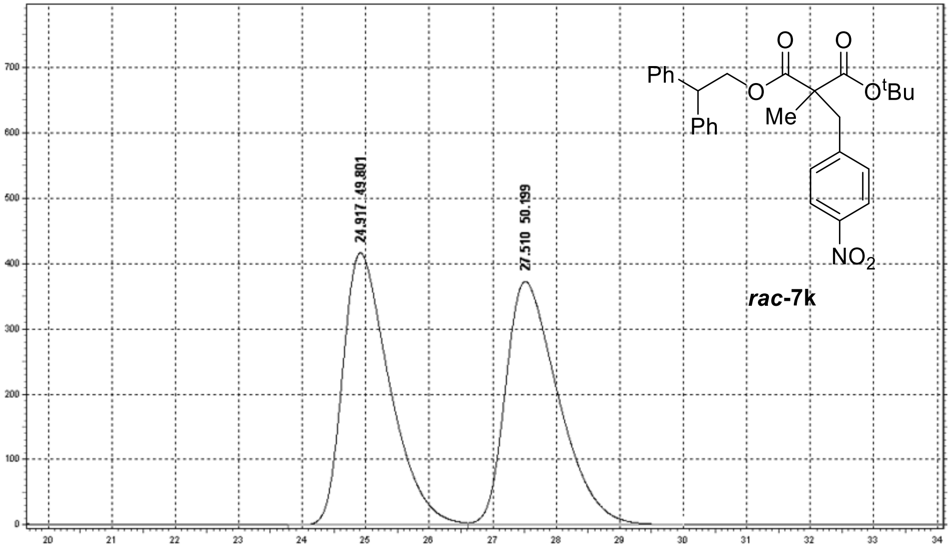

UV Results

Name	Retention Time	Area	Area Percent	Integration Codes
	24.917	85033310	49.801	BV
	27.510	85712677	50.199	VI

Totals		170745987	100.000	

Area Percent Report

Instrument Name: L-2000

Software Version: Version LaChrom 8908800-07

Acquisition Method: Diacel Chiralpak AD-H, Hexane: 2-Propanol = 99 : 1,

λ = 256nm, flow rate= 1 mL/min

Sample ID: ***chiral*-10k**

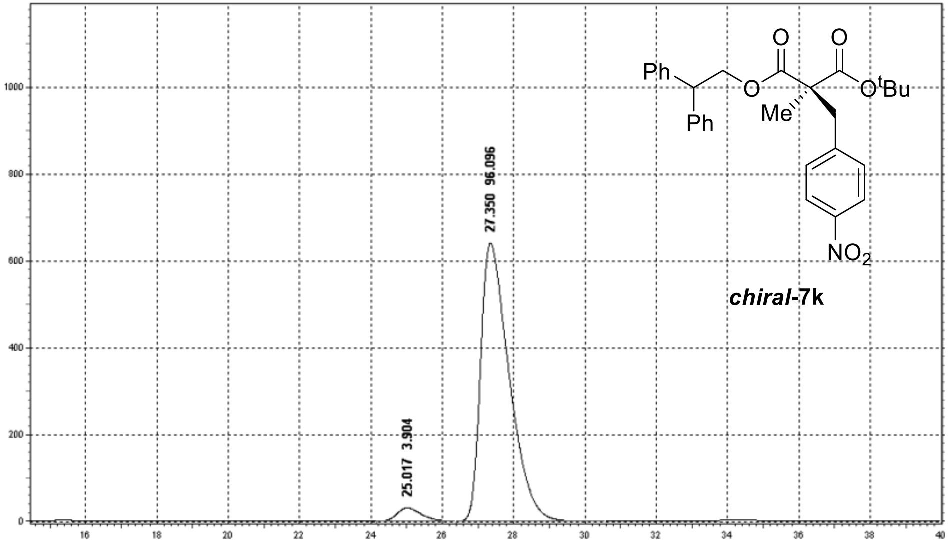

UV Results

Name	Retention Time	Area	Area Percent	Integration Codes
	25.017	5781267	3.904	MM
	27.350	142312386	96.096	MM

Totals		148093653	100.000	

Area Percent Report

Instrument Name: L-2000

Software Version: Version LaChrom 8908800-07

Acquisition Method: Diacel Chiralcel OD-H, Hexane: 2-Propanol = 99 : 1,

λ = 220nm, flow rate= 1 mL/min

Sample ID: ***rac*-10l**

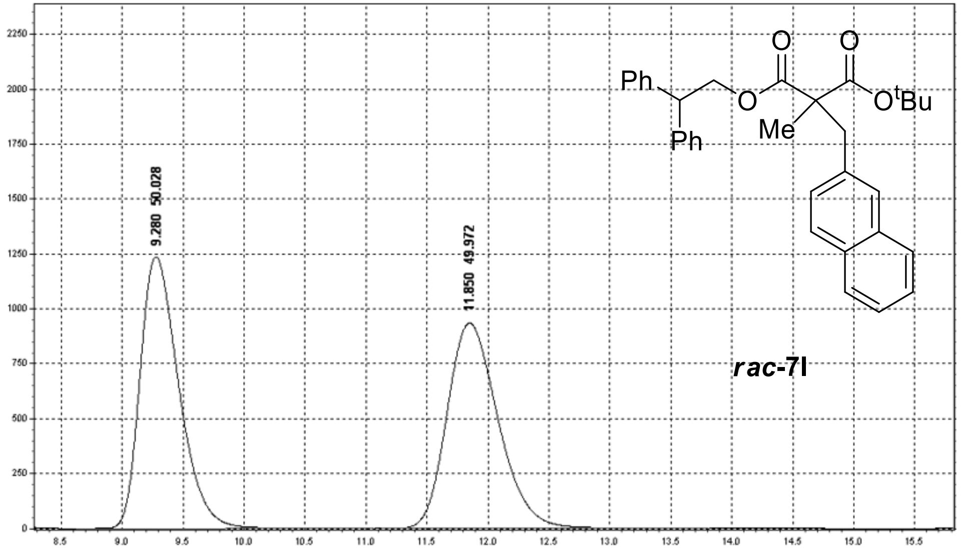

UV Results

Name	Retention Time	Area	Area Percent	Integration Codes
	9.280	109086859	50.028	BB
	11.850	108966250	49.972	BI
Totals		218053109	100.000	

Area Percent Report

Instrument Name: L-2000

Software Version: Version LaChrom 8908800-07

Acquisition Method: Diacel Chiralcel OD-H, Hexane: 2-Propanol = 99 : 1,

λ = 220nm, flow rate= 1 mL/min

Sample ID: ***chiral*-10l**

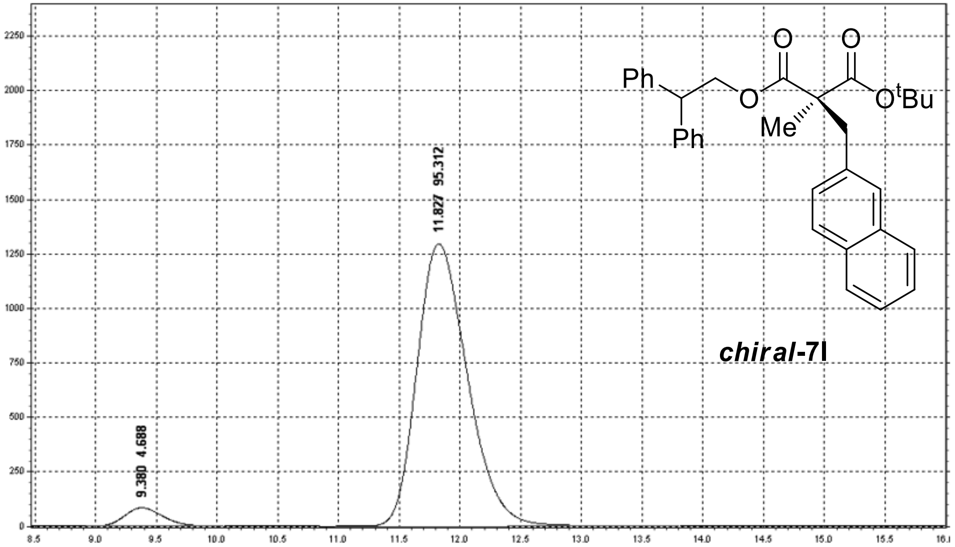

UV Results

Name	Retention Time	Area	Area Percent	Integration Codes
	9.380	7358871	4.688	BB
	11.827	149608847	95.312	BI
Totals		156967718	100.000	

(3) X-ray Crystallographic data

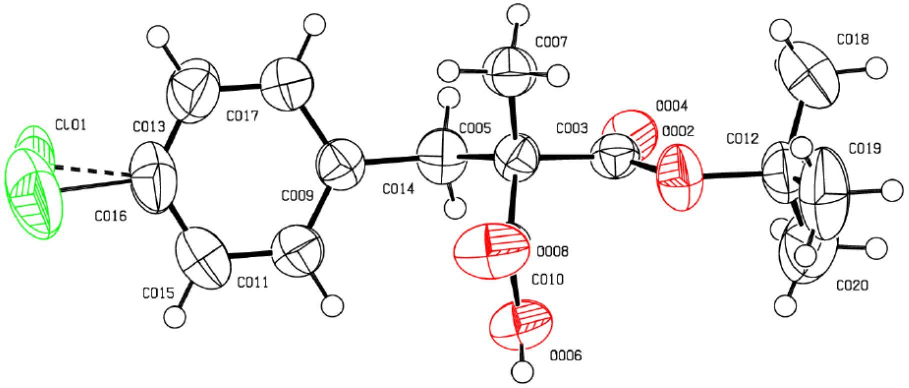

Figure S1. Molecular structure of compound 10i
Red = oxygen,
Black = carbon,
Green = chlorine

<u>Crystal data and structure refinement</u>

Identification code	exp_1342
Empirical formula	$C_{15}H_{19}ClO_4$
Formula weight	298.75
Temperature/K	292.97(15)
Crystal system	orthorhombic
Space group	$P2_12_12_1$
a/Å	6.0571(11)
b/Å	10.7989(16)
c/Å	24.741(4)
α/°	90
β/°	90
γ/°	90
Volume/Å^3	1618.3(4)
Z	4
ρ_{calc}g/cm^3	1.226
μ/mm^{-1}	0.245

F(000)	632.0
Crystal size/mm^3	0.481 × 0.204 × 0.146
Radiation	MoKα (λ = 0.71073)
2Θ range for data collection/°	5.008 to 59.178
Index ranges	$-7 \le h \le 6$, $-13 \le k \le 12$, $-33 \le l \le 23$
Reflections collected	10653
Independent reflections	3863 [R_{int} = 0.0511, R_{sigma} = 0.0656]
Data/restraints/parameters	3863/0/197
Goodness-of-fit on F^2	1.029
Final R indexes [I>=2σ (I)]	R_1 = 0.0575, wR_2 = 0.1050
Final R indexes [all data]	R_1 = 0.1104, wR_2 = 0.1275
Largest diff. peak/hole / e Å^{-3}	0.14/-0.13
Flack parameter	-0.10(7)
